# Deconvolution of synovial myeloid cell subsets across pathotypes and role of COL3A1+ macrophages in rheumatoid arthritis remission

**DOI:** 10.3389/fimmu.2024.1307748

**Published:** 2024-03-26

**Authors:** Xuantao Hu, Ziji Zhang, Lingli Long, Minghu Gu, Weishen Chen, Baiqi Pan, Xiaoyu Wu, Chao Wang, Chengxin Li, Linli Zheng, Puyi Sheng

**Affiliations:** ^1^ Department of Joint Surgery, The First Affiliated Hospital of Sun Yat-Sen University, Guangzhou, China; ^2^ Guangdong Provincial Key Laboratory of Orthopaedics and Traumatology, The First Affiliated Hospital of Sun Yat-sen University, Guangzhou, China; ^3^ Research Center of Translational Medicine, The First Affiliated Hospital of Sun Yat-sen University, Guangzhou, China

**Keywords:** rheumatoid arthritis, single-cell RNA sequencing, myeloid cell, gene deconvolution, macrophage-myofibroblast transition, immunosuppression, remission

## Abstract

**Background:**

Monocyte/macrophage (Mo/Mp) is a critical cell population involved in immune modulation of rheumatoid synovitis (RA) across different pathotypes. This study aims to investigate the contribution of Mo/Mp clusters to RA activity, and the biological function of particular subtypes in RA remission.

**Methods:**

We integrated single-cell RNA sequencing datasets from 4 published and 1 in-house studies using Liger selected by comparison. We estimated the abundance of Mo/Mp subtypes in bulk RNA-seq data from the 81 patients of the Pathobiology of Early Arthritis Cohort (PEAC) using deconvolution analysis. Correlations between Mo/Mp subtypes and RA clinical metrics were assessed. A particular cell type was identified using multicolor immunofluorescence and flow cytometry *in vivo* and successfully induced from a cell line *in vitro*. Potential immune modulation function of it was performed using immunohistochemical staining, adhesion assay, and RT-qPCR.

**Results:**

We identified 8 Mo/Mp clusters. As a particular subtype among them, COL3A1+ Mp (CD68+, COL3A1+, ACTA2-) enriched in myeloid pathotype and negatively correlated with RA severity metrics in all pathotypes. Flow cytometry and multicolor immunofluorescence evidenced the enrichment and M2-like phenotype of COL3A1+ Mp in the myeloid pathotype. Further assays suggested that COL3A1+ Mp potentially attenuates RA severity via expressing anti-inflammatory cytokines, enhancing Mp adhesion, and forming a physical barrier at the synovial lining.

**Conclusion:**

This study reported unexplored associations between different pathologies and myeloid cell subtypes. We also identified a fibroblast-and-M2-like cluster named COL3A1+ Mp, which potentially contributes to synovial immune homeostasis. Targeting the development of COL3A1+ Mp may hold promise for inducing RA remission.

## 1 Introduction

Rheumatoid arthritis (RA) is an autoimmune and systemic inflammatory disease with considerable complexity in pathogenesis (1, 2). It can be classified into three pathotypes based on the differentiated pathological and transcriptional characteristics of synovitis: lymphoid, myeloid, and fibroid. Each pathotype suggests varying levels of inflammation severity and treatment response (3, 4).

The lymphoid pathotype with infiltration, characterized by infiltration of B and myeloid cells, was discovered most in cases with higher-grade inflammation (5). The myeloid pathotype, predominated by myeloid cells solely, also developed severe inflammation but correlated with better response to particular targeted DMARDs (6, 7). Patients with pauci-immune fibroid pathology demonstrated moderate inflammation level but poor treatment responsiveness (5, 7).

Monocytes/macrophages (Mo/Mp), including circulating and synovial tissue-resident populations, play a vital function in the onset, development and maintenance of synovitis (8). However, a recent study defined an anti-inflammatory Mp subset marked by MerTK, which induced synovitis remission and repair response in the synovium of RA (9, 10). Therefore, we assumed that abundance and transcriptomic discrepancy of specific Mo/Mp subtypes could contribute to the diversity of RA synovial pathophysiology.

Although the knowledge about the role of Mp in RA has expanded, the relationship between specific Mp subsets and various synovial pathotypes remains elusive. Herein, we reworked the cluster determination based on data integrated from several published and our unpublished single-cell RNA sequencing (scRNA-seq) datasets. Based on the deconvolution analysis results of bulk RNA-seq data of synovium from untreated RA cohort, we investigated the relevance of characterized Mp subsets to synovial pathology and clinical features. Here, we identified a specific subtype named COL3A1+ Mp and unveiled its potential role in the remission of RA synovitis, which conformed with the differential histology of patients across pathotypes.

Then, we successfully induced THP-1-derived COL3A1+ Mp and briefly investigated its potential effect on RA remission from: 1) an anti-inflammatory secretion program, 2) enhanced cell-to-cell adhesion, and 3) formation of physical barrier at the synovial lining.

## 2 Materials and methods

### 2.1 ScRNA-seq of synovial tissue

The present study followed the Guidelines of the Declaration of Helsinki and approved by the institutional review board.

With the informed consent, we collected removed synovial tissue from 15 RA patients during arthroplasty operation for RA proved by the 2010 ACR/EULAR diagnostic criteria. Synovial tissue was washed using cold PBS, dissociated into 1-3 mm fragments, and sequentially digested with a double enzyme mixture (collagenase I, 200 U/ml; Sigma-Aldrich, USA; hyaluronidase, 50 U/ml, Absin, China) for 1 h and TrypLE (0.25%; BL512A, Biosharp, China) in PBS at 37°C for 15 min each in a shaker. By filtering the digest mixture using the 70-μm cell strainer after each round, supernatant enriched with cells was collected, combined, while the tissue fragments entered the next round. Red blood cell lysis buffer (CW0613S, Cowin Bio, China) and DNase (1 mg/ml; 10104159001, Roche, Switzerland) were used only if cell precipitation was obvious red or cell clumping formed. Then cells were washed with PBS and resuspended. Fluorescence-activated cell sorting (FACS) was used to isolate Mp for in-house scRNA-seq data (refer to the section 2.5). A total of 42,009 Mp were collected.

Single cells were barcoded and converted into single-cell RNA sequencing (scRNA-seq) libraries using the Chromium Single-Cell 5’ kit (V1, 10X Genomics). To ensure a sufficient number of cells (8,000-10,000) in each library and quality, they were quantified and controlled using the Bioanalyzer 2100 (G2939BA, Agilent, USA) and a high-sensitivity DNA kit (5067-4626, Agilent, USA). The libraries were then sequenced on an Illumina NovaSeq 6000 platform (Illumina, USA) with an average of 31,439 reads per cell. The data was mapped to the human genome (GRCh38) and finally separated into individual samples using the CellRanger pipelines (V.3.1.0, 10X Genomics).

The datasets utilized in the study are accessible to the public and described in **Supplementary Text 1**.

### 2.2 Integration of scRNA-seq datasets and clustering

To sum up, a total of 67,321 from RA synovium were included for further analysis.

The quality control of the scRNA-seq dataset was performed according to the canonical workflow of Seurat (V.4.0.5) package in R (V.4.2.2) (refer to the parameters in the Table attached in **Supplementary Text 1**) (11). We saved a group of pre-annotated endothelial cells from the datasets SDY1599_SCP469 as a reference object in the integration procedure reflecting the over-integration. We tried four approaches of data integration, including Seurat, Harmony (harmony, V.0.1.1), Liger (rliger, V.1.0.0), Atomic sketch (Seurat, branch feat/dictionary), which have been demonstrated to be superior compared to other protocols (12). Default arguments were used for each integration method if not otherwise stated. Further details about the integration process can be found in **Supplementary Text 1**.

To determine the differentially expressed genes (DEG) between cell subsets, we performed the Wilcoxon rank-sum test and used Bonferroni correction for *p* values adjustment. A minimum log2 FC of 0.25 and a maximum adjusted *p* value of 0.05 were adopted to filter the DEG. Kyoto Encyclopedia of Genes and Genomes (KEGG) pathways enrichment analysis of the DEG was conducted using the clusterProfiler (V.4.6.0).

Since endothelial cells only exist in dataset SDY1599_SCP469, the distribution correctness of this pre-labeled population across the datasets was used to evaluate the robustness and accuracy of integration.

### 2.3 Pseudotime analysis

We isolated annotated IL1B+ Mp and COL3A1+ Mp from the integrated data, and then combined it with pre-labeled fibroblast from dataset SDY1599_SCP469. Subsequently, the data comprising of these 3 cell types was used for pseudotime analysis.

Single-cell developmental process was investigated using the Monocle 2 R package (V.2.26.0) (13). The trajectory was inferred according to transcriptomic changes in each cell. Along this trajectory, cells were dropped with a scale that reflects the function of the trajectory, the “pseudotime”. The ordering of cells along the trajectory enables the identification of genes that change over pseudotime. The most significant genes that display similar trends over pseudotime were clustered. The analysis was carried out using the default argument provided by the package (13).

### 2.4 Bulk RNA-seq deconvolution of Mo/Mp subsets across RA pathotypes

The study utilized bulk RNA-seq data from synovial tissue of 81 early RA patients from PEAC with treatment-naive status and symptoms lasting less than 12 months (3). The related clinical characteristics of these patients were also obtained, indicating that 16 had a fibroid, 45 had a lymphoid, and 20 had a myeloid pathotype.

The FASTQ files were downloaded from the ArrayExpress genomics data collection in BioStudies database at the European Bioinformatics Institute with E-MTAB-6141 access code. Raw counts for each gene were obtained using featureCounts and STAR for reads-to-features mapping and hg19 genome annotation (14, 15).

The MCP-counter R package (V.1.2.0) was used for deconvolution analysis by quantifying the signature enrichment score of cell subsets in bulk transcriptome data (16).

We utilized pairwise-Wilcoxon test for the comparison of cell levels among pathotype groups. Correlation analysis of Spearman approach was performed to investigate disease activity measurements across the synovial pathotypes. The results were adjusted for multiple testing using false discovery rate (FDR) control.

### 2.5 Fluorescence-activated cell sorting and flow cytometry analysis

We employed FACS to isolate Mp for in-house scRNA-seq. Briefly, the single cell suspensions from the 15 samples underwent labeling with Fixable Viability Stain 700 (564219, BD Horizon) and antibody to CD68 (BV421, 564943, BD Horizon) for 15 min in stain buffer (554656, BD Pharmingen) according to manufacturer’s instruction. The viable CD68+ macrophages (Mp) were gated and sorted based on the negative Fixable Viability Stain 700 staining and positive CD68 expression using a FACSAria (BD Biosciences). The cells were collected into complete RPMI1640.

We employed flow cytometry to confirm the existence of specific Mo/Mp subtypes in the synovial tissue of RA patients (n = 5 for each pathotype). The tissue digestion workflow was similar to that for scRNA-seq but with an extended the time of the double enzyme mixture incubation for 2 h. Subsequently, we resuspended the cell pellets in PBS and stained them for flow cytometry. We blocked nonspecific Fc binding with Fc Block (564219, BD Pharmingen, USA) and labeled cell suspensions with Fixable Viability Stain 700 (564219, BD Horizon) to distinguish between living and dead cells. CD11b antibody (APC, 553312, BD Pharmingen, USA) and was used to identify Mo/Mp. COL3A1+ Mp were detected according to the expression of CD68 and COL3A1 in the Mo/Mp population using the antibodies (CD68-BV421, 564943, BD Horizon; COL3A1-FITC, sc-271249, Santa Cruz, USA). Cell samples were measured by a cell analyzer (LSRFortessa, BD). Flow cytometry data was analyzed using Flowjo (V.10.8.1, Flexera Software).

### 2.6 Multicolor immunofluorescence staining

Synovium from RA patients was collected and fixed with 4% paraformaldehyde and embedded in paraffin, and cut into 5 μm sections. Sections were deparaffinized using xylol and rehydrated using graded ethanol and TBS. For antigen retrieval, the sections were cooked in sodium citrate buffer (pH 6.0) in a microwave at full power for 5 min, then at reduced power (30%) for another 90 s in a pressure cooker. We left the slides cool for 1 h before washing them in double-distilled water for 3 min. Endogenous peroxidase in tissue was eliminated by treating it with 3% H_2_O_2_ for 10 minutes.

To minimize nonspecific binding, we incubated sections with 10% normal human serum and 10% serum from the species in which the secondary antibodies were raised at room temperature for 30 min. Sections were incubated with primary antibodies against CD68, COL3A1, CD206 (MRC1), CD45, α-SMA (ACTA2), TREM2, or PDPN overnight at 4 °C. The next day, sections were washed twice for 5 min in TBS/0.025% Triton X-100 (TBST) and then incubated with secondary antibodies diluted in TBS/1% BSA at room temperature for 1 h. The antibodies used were summarized in **Table 1**. After incubation, we stained sections with mounting media containing DAPI. We visualized sections with an Olympus BX53 confocal microscope (n = 3 for each pathotype). Cell detection and quantification of target cell populations was conducted using Qupath (0.4.3) according to official guidelines. For the CD68+COL3A1+ Mp quantification, at least 5 regions of interest (ROI) for each section were selected (No. ROI = 15 for each pathotype). For the CD68+COL3A1+CD163+ Mp quantification, at least 2 ROI for each section were selected (No. ROI = 6 for each pathotype).

**Table 1:** Antibodies and labels used for immunohistochemistry.

Antibody Name	Host	Dilution	Source
CD68	Mouse	NA	Kit-0026, Maixin, China
COL1A1	Rabbit	1:1000	BA0325, Boster, Chiina
α-SMA	Rabbit	1:1000	AF1032, Affinity, China
CD206	Rabbit	1:1000	24595, CST, USA
Goat Anti-Rabbit IgG (HRP)	Goat	1:4000	ab205718, Abcam, USA
Goat Anti-mouse IgG (HRP)	Goat	1:4000	ab205719, Abcam, USA
FITC	NA	1:300	11060, AAT Bioquest, USA
Cy3	NA	1:400	11065, AAT Bioquest, USA
Cy5	NA	1:400	11066, AAT Bioquest, USA
Cy7	NA	1:500	11064, AAT Bioquest, USA

NA, Not Applicable.

### 2.7 Cell culture, Mp differentiation and treatment

Human monocyte (THP-1) cell line (ATCC, USA) was cultured in RPMI 1640 (C11875500BT, Thermo Fisher, USA) supplemented with 10% fetal bovine serum (15140122, Thermo Fisher, USA) and antibiotics (A3160801, Gibco, USA) at 37°C with 5% CO2.

Vitamin C (Vit C) is essential for the production of hydroxyproline, which is demanded by the correct assembly of triple helix structure and secretion of procollagen (17). Evidence indicated that the solemnly addition of Vit C did not lead to a significant selection of cell types responsive to it. Therefore, we added 50 ng/ml Vit C into the culture medium used in experiments in which COL3A1+ Mp was induced.

We induced THP-1 monocyte differentiation into inactive (M0) and following polarization into M1 macrophages according to the protocol described before. In brief, THP-1 monocytes were incubated with 100 nM phorbol 12-myristate 13-acetate (PMA) (P8139, Sigma–Aldrich, US) for 48 h to yield M0 macrophage. Then M1 macrophages were obtained by treating M0 macrophages with 10 pg/mL of bacterial lipopolysaccharides (LPS) (L4391, Sigma-Aldrich, Germany) and 20 ng/mL of interferon-gamma (IFN-γ) (300-02, PeproTech, Australia) for 48 h and 96 h (18).

For the treatment, M1 macrophages were exposed to 10 ng/ml TGF-β1 (96-100-21-10, PeproTech, Australia).

### 2.8 Real-time qPCR

The total RNA from the culture was isolated with the SteadyPure Universal RNA Extraction Kit (AG21017, Accurate Biology, China). Evo M-MLV RT Master Mix (AG11706, Accurate Biology, China) was used for cDNA synthesis through reverse transcription. SYBR Green Pro Taq HS Premix (04707494001, Roche, Switzerland) was used for real-time quantitative PCR (RT-qPCR) according to the manufacturer’s instruction on CFX96 Touch (Bio-rad) (n = 3). The primer sequences are listed in **Supplementary Table 1**.

### 2.9 Masson’s trichrome staining

We seeded 2.5×10^5^ THP-1 cells on a glass slide per well in 24-well plates. M1 induction and TGF-β1 treatment were performed as above mentioned to obtain COL3A1+ Mp. Then, we deployed modified Masson’s staining (G1346, Solarbio, Switzerland) according to the manufacturer’s instructions (n = 3). The light microscope images were obtained and analyzed using ImageJ (V1.5.4, National Institutes of Health, USA).

### 2.10 M1-to-COL3A1+-Mp adhesion assay

First, 2.5×10^5^ THP-1 cells were seeded in 24-well plates and differentiated into M1 or COL3A1+ Mp as mentioned above. Subsequently, 2.5×10^5^ calcein-labeled THP-1-derived M1 were added on top of M1 or COL3A1+ Mp culture and incubated for 6 h. Then, the wells were rinsed with PBS and fixed with 4% PFA. The image of M1 Mp adhesion to M1 or COL3A1+ Mp were observed using a Leica DMI4000 microscope (n = 3).

### 2.11 Coculture of COL3A1+ Mp and M1 macrophage

The coculture system is similar to the adhesion assay above, but leaves added M1 Mp on the top and cocultured with pre-seeded COL3A1+ Mp for 48 h.

### 2.12 Statistical analysis

Unless otherwise stated, the data is presented as mean ± standard deviation (SD). Statistical analysis was conducted using the Student’s t-test after confirming the homogeneity of variances and normal distribution of data. A p-value < 0.05 was considered statistically significant.

## 3 Results

### 3.1 ScRNA-seq datasets integration

We obtained scRNA-seq data from 61 synovial tissues of RA patients from 3 previous studies and an in-house dataset (refer to **Supplementary Text 1**).

**Figure 1:**
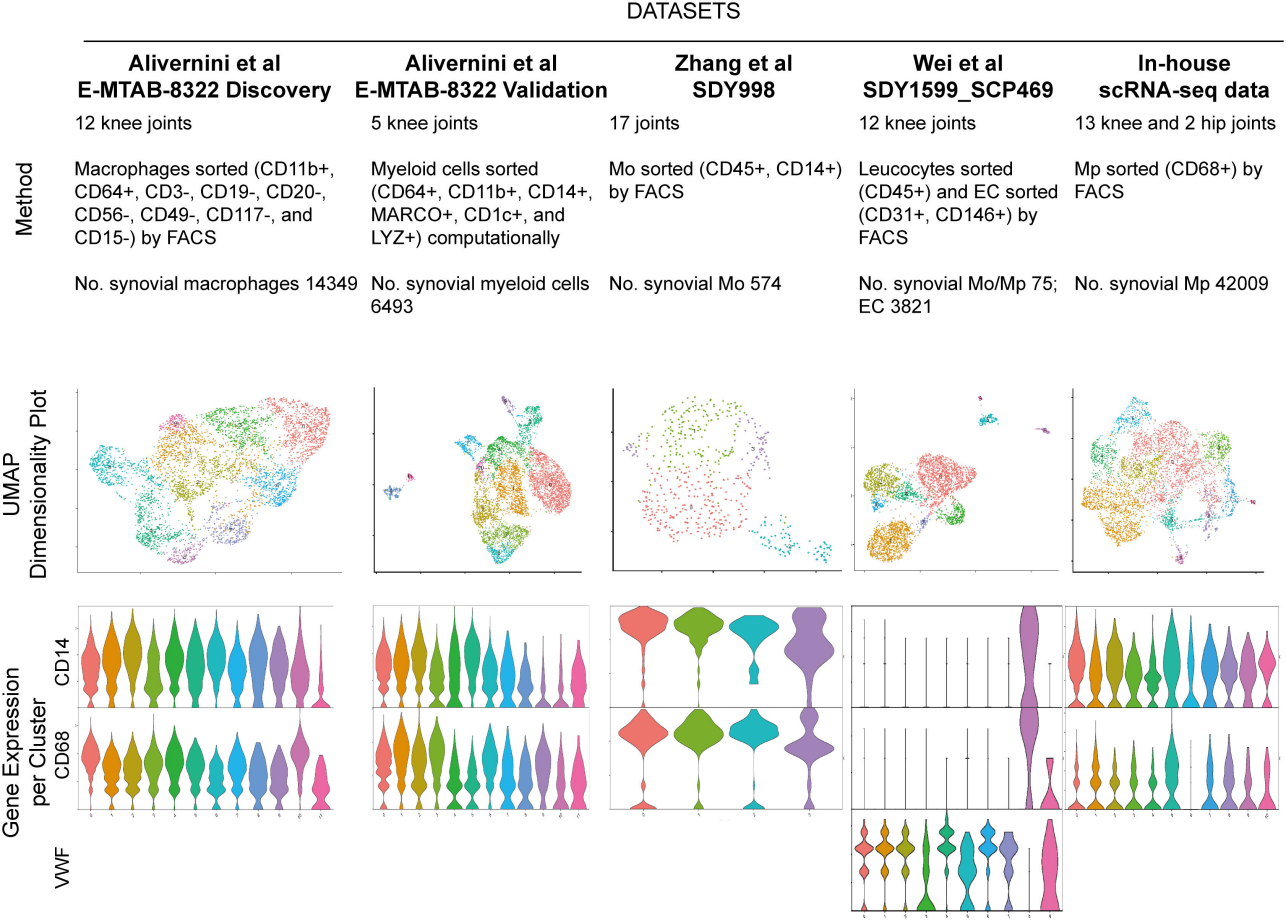
Five RA synovial myeloid cell datasets with respective description of study method, cell number, UMAP visualization and violin plot for marker of Mo (CD14), Mp (CD68) and endothelial cell (VWF). Endothelial cells in SDY1599_SCP469 were included for the following selection of integration method. UMAP: Uniform Manifold Approximation and Projection for Dimension Reduction.

Target cells (Mo, Mp and endothelial cells) from each dataset were isolated according to the metadata provided in the original studies (refer to **Figure 1**, row 1).

We employed Uniform Manifold Approximation and Projection for Dimension Reduction (UMAP) to visualize the Mp/Mo clusters (**Figure 1**, row 2). The number of Mo/Mp in E-MTAB-8322-Disc and in-house were much higher than those in other datasets (refer to **Figure 1**) (10). To balance the cell number discrepancy among datasets, we selected 5000 Mo/Mp from each of the two studies by random sampling. A total of 20963 target cells were used for further analysis.

### 3.2 Generation of Mo/Mp gene signature

**Figure 2:**
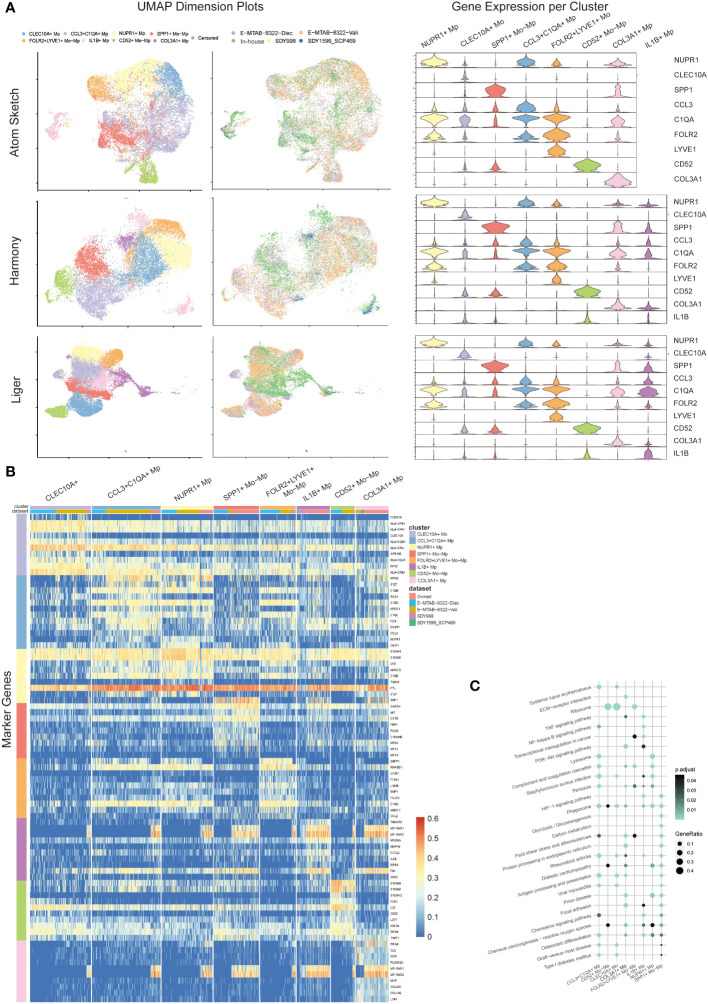
Mo/Mp subtypes identification. **(A)** UMAP visualization with cell clustering or dataset identity, and violin plots showing the expression makers of each subset in different integration methods (Atom sketch, Harmony and Liger). **(B)** Heatmap showing 10 markers expression for each Mo/Mp subtype. **(C)** Bubble plot of KEGG pathways enrichment analysis across Mo/Mp subtypes, depicting the top 20 pathways.

Datasets integration and clustering using Atom Sketch, Harmony and Liger approaches respectively detected similar Mo/Mp clusters characterized by previously described marker genes (CLEC10A, NUPR1, SPP1, CCL3, C1QA, FOLR2, LYVE, CD52, IL1B) and a particular cluster marked by COL3A1 (see **Figure 2**). The top 20 marker genes for each Mo/Mp cluster determined by Atom Sketch, Harmony and Liger are demonstrated in **Supplementary Tables 2**–**4**.

The CCL3+C1QA+ Mp subtype showed high expression of C1QB, C1QA and CCL3, which is involved in the chemotaxis of inflammatory cells (19, 20). The CD52+ Mo-Mp subtype expressed CD52, S100A9 and S100A8, which has been reported as a bi-directional regulator in inflammation of autoimmune diseases via T cell activation and Treg induction (21, 22). The CLEC10A+ Mo subtype was featured by high expression of CLEC10A and HLAs. This subtype represents monocyte-derived canonical dendrite cells which play a role in antigen presentation (10, 23). The FOLR2+LYVE+ Mo-Mp subtype was characterized by LYVE1 and FOLR2 expression, which was previously reported to be mainly localized in the lining layer of remitted RA and around blood vessels in active RA, playing a role in immunosuppression (10, 24). The IL1B+ Mp subtype represented the main population of M1 macrophages with high expression of TNFAIP3, MMP19 and IL1B (1, 20). The NUPR1+ Mp subtype was featured by the expression NUPR1. Previous studies suggested that NUPR1+ Mp negatively correlated to tissue inflammation, possibly via modulating cell death and tissue remodeling (1, 25). The SPP1+ Mo-Mp subtype was marked by SPP1, which has a pro-inflammatory phenotype with migration and bone resorption property (1, 10, 20). The COL3A1+ Mp subtype demonstrated high expression of collagen genes including (COL1A1, COL1A2 and COL3A1), which was uncharted in the source studies. Previous studies identified collagen-expressing myeloid cells as different subtypes, including fibrocyte and macrophage-myofibroblast cells (26, 27). An additional subtype of Mo with high expression of IL8, ERO1L and CD97 were identified by Liger. Since this cluster contains only 58 cells and not specified in previous studies, this subtype is annotated as “censored” and not included in further analysis.

We distinguished the data source of cells with color-coding UMAP visualization, confirming that the above-mentioned Mo/Mp subtypes were identified across all datasets (**Figure 2A**). However, the distribution of these subtypes varied across the datasets (**Supplementary Figure 1**). This variation may be attributed to differences in materials, methods, patient selection (e.g., pathotype and activity), joint location, etc.

Since Liger integration identified a precise proportion of endothelial cells in SDY1599_SCP469 rather than in other datasets (**Supplementary Figure 2**), we chose data from Liger integration for further analysis. Due to the small number of myeloid cells, we excluded the data of SDY1599_SCP469 dataset from further analysis.

We further conducted KEGG pathway analysis on the Mo/Mp subtypes (**Figure 2C**; **Supplementary Table 5**). Differentially expressed genes (DEG) of CCL3+C1QA+ Mp were enriched in terms such as ‘*staphylococcus aureus* infection’ and ‘antigen processing and presentation’, supporting their role in chemotaxis. Genes of CD52+ Mo-Mp subtype were significantly enriched in ‘ribosome’, supporting their function of active protein synthesis in immunity. CLEC10A+ Mo genes were enriched in pathways associated with active adaptive immune response, such as ‘ribosome’, ‘antigen processing and presentation’, and ‘graft-versus-host disease’. Enriched terms for FOLR2+LYVE+ Mo-Mp included ‘complement and coagulation cascades’ and ‘ribosome’. The most significant signaling pathways for IL1B+ Mp were ‘TNF signaling pathway’, ‘Osteoclast differentiation’ and ‘NF-kappa B signaling pathway’. The DEG of NUPR1+ Mp enriched in terms like ‘lysosome’, ‘complement and coagulation cascades’ and ‘phagosome’. Unique terms for SPP1+ Mo-Mp were ‘HIF-1 signaling pathway’, ‘glycolysis/gluconeogenesis’ and ‘carbon metabolism’, suggesting that this subtype has enhanced glycolysis. Interestingly, the DEG of COL3A1+ distinctively enriched in terms including ‘ECM-receptor interaction’, ‘Protein processing in endoplasmic reticulum’, and ‘Focal adhesion’, which conform with its highly expression of COLs genes.

### 3.3 Dynamic transition between IL1B+ Mp and COL3A1+ Mp

**Figure 3:**
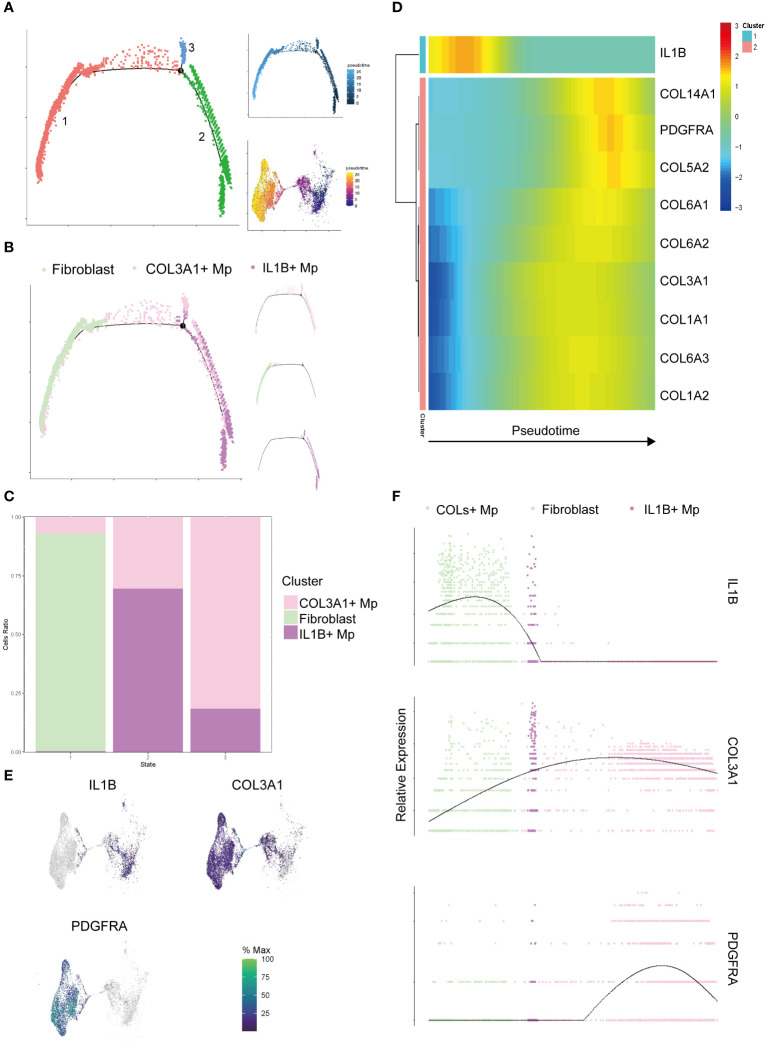
Pseudotime analysis of the target cell subtypes (IL1B+ Mp, COL3A1+ Mp, and fibroblasts). **(A)** The trajectory states of all the target cells were visualized, with the pseudotime trajectory and UMAP visualization displayed on the top and bottom right. **(B)** Distribution of cell subtypes mapping along the trajectory, with split-by-subtypes visualization on the right. **(C)** Stack histogram showing the distribution of the cell subtypes in trajectory states 1, 2, 3. **(D)** Heatmap visualizing the most significant genes that covary along the trajectory (state 2 on the left and state 1 on the right). **(E)** UMAP mapping the gene expression levels IL1B, COL3A1 and PDGFRA. **(F)** Jitter plot showing the gene expression levels IL1B, COL3A1 and PDGFRA along the trajectory.

Following the completion of clustering and annotation, we observed the presence of a special subtype, COL3A1+ Mp. When reviewing the UMAP plot of Liger integration, a continuum or gradient distribution between COL3A1+ Mp and IL1B+ Mp was found, which indicated a potential transitional relationship between them. COL3A1+ Mp expressed different types of collagens, which resembled extracellular matrix (ECM) secretion phenotype of fibroblasts. To elucidate the dynamics of cell transition, we included fibroblasts from SDY1599_SCP469 and used monocle 2 to examine the relationship among COL3A1+ Mp, IL1B+ Mp, and fibroblasts (**Figure 3**).

The cell ordering workflow identified a continuum of 3 distinct cell states along a trajectory (**Figure 3A**). When cell identities were mapped, cell subtypes appeared to be distributed along the trajectory starting from IL1B+ Mp, passing through COL3A1+ Mp and ending with fibroblasts (**Figures 3B, C**). A minor branch of cell state 3 was mainly formed by COL3A1+ Mp.

It has been reported that Mp has potential in fibroblast-like transition (28–30). Considering the plasticity and heterogeneity of canonical Mp, we *a priori* defined the State 1, which mainly consists of IL1B+ Mp, as the root (starting point) of the trajectory for further analysis. We conducted differential analysis and identified a list of genes with significantly altered expression over pseudotime (**Supplementary Table 6**).

The heatmap in **Figure 3D** illustrates the alteration of marker genes of the three subtypes (IL1B+ Mp: IL1B; COL3A1+ Mp: COL3A1; fibroblast: PDGFRA) along the pseudotime trajectory. Also, we investigated genes of various collagens, which are consistently expressed in fibroblasts and potentially in COL3A1+ Mp.

The expression of most collagen genes, such as COL3A1, COL6As and COL1As, gradually increased along the trajectory (**Figures 3D–F**).

Conclusively, we integrated 5 datasets and reaffirmed the existence of COL3A1+ Mp subtype. Pseudotime analysis suggested a potential plasticity of Mp from canonical M1 to a fibroblast-like subtype (though not fibroblast).

### 3.4 Discrepancy of Mo/Mp subtype abundance across synovial pathotypes

We conducted deconvolution analysis on bulk transcriptomics data of RA synovial tissue from PEAC. The result revealed a disparate abundance of Mo/Mp subtypes in different pathotypes.

**Figure 4:**
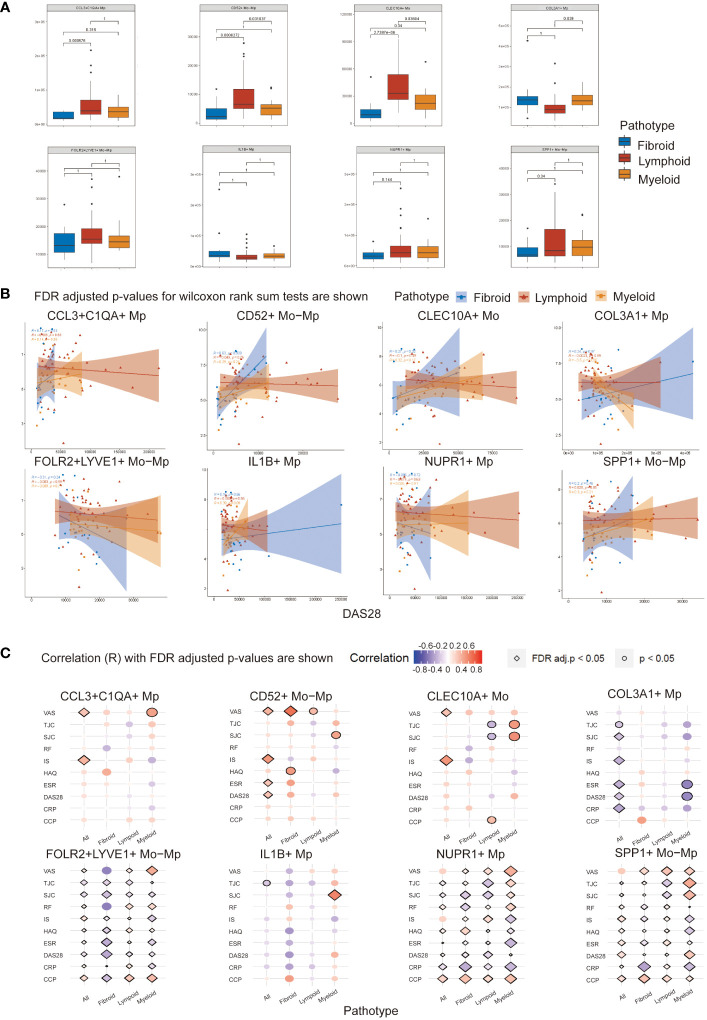
Mo/Mp subtypes abundance and Spearman correlation with disease activity indicators across RA pathotypes. **(A)** Abundance of different subtypes between pathotypes. **(B)** Correlation between abundance of cell subtypes and DAS28 score across synovial pathotypes. **(C)** Bubble plot displaying the correlation of various clinical measurements with Mo/Mp subtypes across pathotypes. VAS, visual analog scale; TJC, tender joint count; SJC, swollen joint count; RF, rheumatoid factors; IS, inflammatory score; HAQ, health assessment questionnaire; ESR, erythrocyte sedimentation rate; DAS, disease activity score; CRP, C-reactive protein; CCP, cyclic citrullinated peptide.

As shown in **Figure 4A**, CCL3+C1QA+ Mp, CLEC10A+ Mo and CD52+ Mo-Mp were most enriched in the lymphoid pathotype, while COL3A1+ Mp count expanded in the myeloid pathotype. The number of COL3A1+ Mp in the myeloid pathotype was not statistically higher than in fibroid RA.

### 3.5 Correlation analysis between Mo/Mp subtypes and clinical features

To investigate the connection of the Mo/Mp subtypes with pathological characteristics and activity of RA, we performed correlation analysis using the abundance of cell subtypes and clinical parameters (**Figures 4B, C**; **Supplementary Table 7**). The abundances of Mo/Mp subtypes showed no significant differences between men and women, seropositive and seronegative patients, or different age groups.

For all patients without distinguishing between pathotypes, CCL3+C1QA+ Mp (R = 0.37, adjusted *p* value < 0.05), CD52+ Mo-Mp (R = 0.48, adjusted *p* value < 0.01) and CLEC10A+ Mo (R = 0.52, adjusted *p* value < 0.01) Mp showed weak or medium positive correlation with inflammation score (IS). The Pain on Visual Analogue Scale (VAS) exhibited a similar pattern. The presence of COL3A1+ Mp had a weak or medium negative correlation with swollen joint count (SJC) (R = -0.26, adjusted *p* value = 0.04), IS (R = -0.33, adjusted *p* value = 0.01), ESR (R = -0.34, *p* value < 0.01), Disease Activity Score-28 (DAS28) (R = -0.26, adjusted *p* value = 0.04), and CRP (R = -0.37, *p* value ≤ 0.01).

When distinguishing pathotype, there was a medium or strong positive correlation between CD52+ Mo-Mp presence and VAS (R = 0.69, adjusted *p* value = 0.03) in fibroid pathotype.

Notably, consistent with its reported role as an ambiguous regulator in inflammation, SPP1+ Mp subtype showed a significant correlation with different disease activity indicators, but the direction of correlation is inconsistent (31, 32). Although previous studies have indicated their potential roles, FOLR2+LYVE1+ Mo-Mp and NUPR1+ Mp also demonstrated ambiguous results in the correlation analysis here (1, 10). The abundance of IL1B+ Mp appeared to have limited correlation with RA activity indicators.

These findings suggest that rarely reported COL3A1+ Mp may play a critical role in immune repression in RA synovium.

### 3.6 Identification of synovial pathotype

The pathotype of histological samples were assessed using previously described approach (33).

### 3.7 Validation of COL3A1+ Mp existence *in vivo* and correlation with RA pathotypes

**Figure 5:**
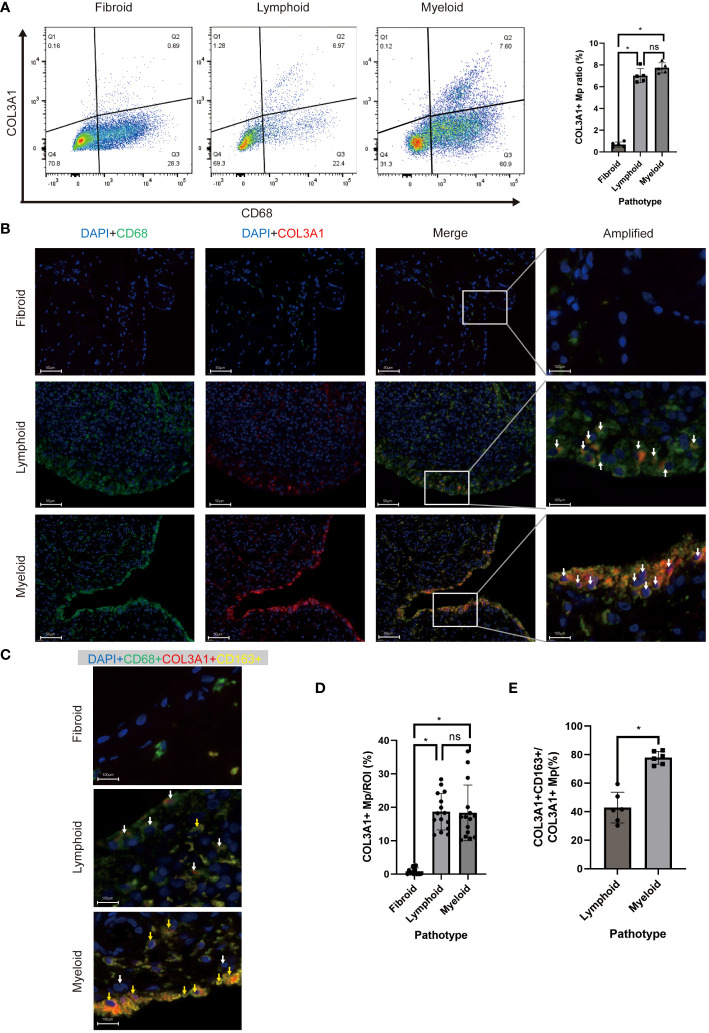
*In vivo* existence of COL3A1+ Mp across synovial pathotypes. **(A)** Representative scatter plot and percentage of CD68+COL3A1+ cells analyzed by flow cytometry using synovium-derived cell suspension. (n = 5; *P < 0.05) **(B, C)** are representative images of immunofluorescence showing CD68+COL3A1+ (white arrows) and CD68+COL3A1+CD163+ cells (yellow arrows). **(D, E)** present the comparison of CD68+COL3A1+ (No. ROI = 15) and CD68+COL3A1+CD163+ (No. ROI = 6) cell abundance across pathotypes. (*P < 0.05). ns, not significant.

To examine the existence of COL3A1+ Mp in RA synovium, we identified the COL3A1+ Mp cell population using flow cytometry (**Figure 5A**). In synovial cell suspension from patients of different pathotypes, lymphoid and myeloid types shared a similar ratio of COL3A1+ Mp (7.02 ± 0.65; 7.74 ± 0.48), which is significantly higher when compared to fibroid synovium (0.68 ± 0.23; *vs* lymphoid: *p* < 0.01; *vs* myeloid: *p* < 0.01). Additionally, osteoarthritis synovia contained a significantly lower ratio of COL3A1+ Mp (0.46 ± 0.07) when compared with RA synovia (lymphoid type) (*p* < 0.01) (**Supplementary Figures 3A, B**). Notably, the Mp clusters in the dotplot did not form entirely disjoint clusters, suggesting a transition relationship between COL3A1+ and COL3A1- Mp. This is further illustrated by the continuous distribution of COL3A1+ Mp and IL1B+ Mp in UMAP and pseudotime trajectory (**Figure 2A**; **Figure 3B**).

The presence of COL3A1+ Mp drew our attention to fibroblast-like myeloid cells.

To investigate the presence and distribution of the fibroblast-like myeloid cell populations across RA pathotypes, we deployed multicolor immunofluorescence in the synovium sections of patients to assess the expression of their markers, including CD45, CD68, α-SMA, COL3A1, and CD163 Sections of 9 patients (lymphoid: myeloid: fibroid = 3: 3: 3) were included.

COL3A1+ Mp (CD68+COL1A1+), Fibrocyte (CD45+COL1A1+), and MMT cells (CD68+ATCA2+) were detected in all sections but with different abundance across the pathotypes (**Figures 5B, D**; **Supplementary Figures 4A, B**). The abundance of COL3A1+ Mp (**Figures 5B, D**), fibrocyte and MMT cells (**Supplementary Figures 4A, B**) and in lymphoid and myeloid synovium section were significantly higher than those in fibroid type. Interestingly, even though the abundance of COL3A1+ Mp showed no significant difference between lymphoid and myeloid types, the M2-like COL3A1+ Mp (CD68+COL3A1+CD163+) abundance in myeloid type was significantly higher than that in the lymphoid type (**Figures 5D, E**). These findings are consistent with deconvolution and correlation analysis of COL3A1+ Mp, which indicate COL3A1+ Mp enrichment of COL3A1+ Mp in myeloid and lymphoid rather than fibroid pathotype. Moreover, it suggests a different abundance of M2-like COL3A1+ Mp among pathotypes, potentially contributing to variations in the immune microenvironment.

### 3.8 Induction and characterization of COL3A1+ Mp

In contrast, COL3A1+ Mp as we defined here shared a similar phenotype with MMT cells, except for differentially high-level expression of specific fibroblast markers (COL3A1) instead of α-SMA (**Supplementary Figure 5A**). We hypothesize that COL3A1+ Mp is a specific type of MMT cell present in RA synovium.

**Figure 6:**
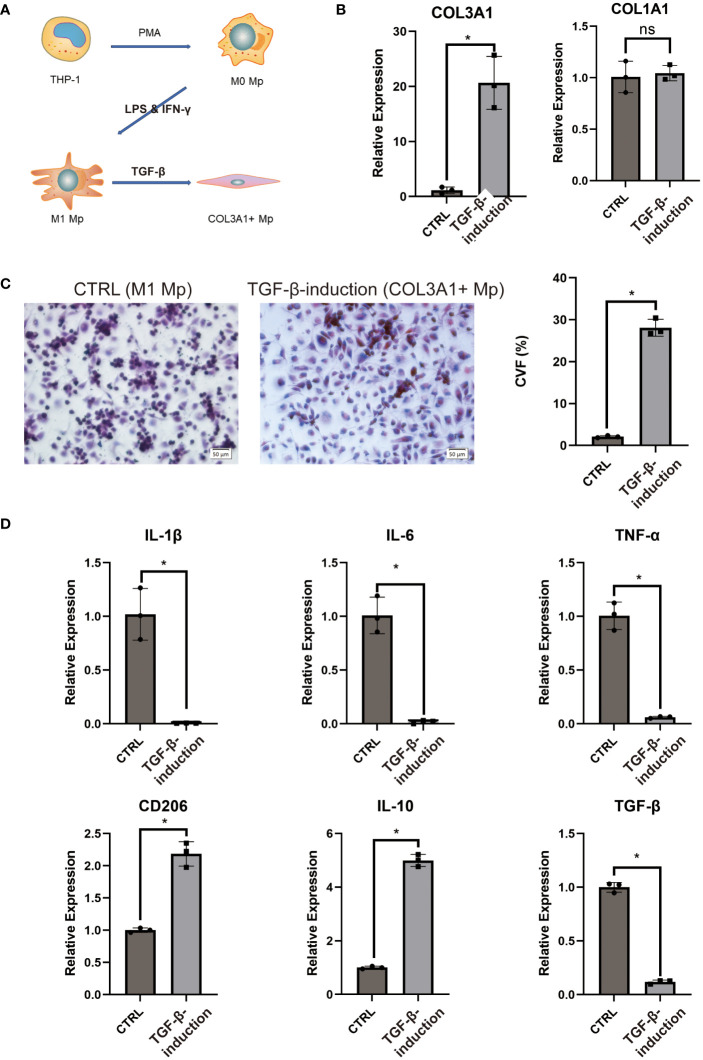
COL3A1+ Mp induction and characterization. **(A)** proposed model of COL3A1+ Mp induction. **(B)** RT-qPCR analysis of COL3A1 mRNA expression in the control (M1 Mp) and TGF-β1-induction group (COL3A1+ Mp). (n = 3; *P < 0.05) **(C)** Representative image and collagen volume fraction (CVF) quantification of Masson’s trichrome staining for cell culture on glass slide from the control and TGF-β1-induction group. **(D)** RT-qPCR analysis of M1-produced pro=inflammatory cytokines (IL-1β, IL-6 and TNF-α) and M2-produced anti-inflammatory cytokines and marker (IL-10, TGF-β1 and CD206). (n = 3; *P < 0.05). ns, not significant.

Emerging evidence has indicated that TGF-β1 stimulation could induce transdifferentiation from Mp to MMT cells (28, 29). This aligns with the KEGG enrichment analysis of COL3A1+ Mp indicating enrichment for “response to TGF” in the top biological process terms (**Supplementary Figure 5B**). Moreover, as the results of pseudotime analysis and RA pathophysiology suggest a potential transition from IL-1b-producing M1 Mp to anti-inflammatory COL3A1+ Mp (**Figure 3B**), we attempted to induce COL3A1+ Mp by stimulating THP-1-derived M1 Mp with TGF-β1 for 96 h (**Figure 6A**).

Firstly, we examined the transcriptional expression of COL1A1 and COL3A1 using RT-qPCR. It was observed that the mRNA expression of COL3A1 significantly increased after 96 h TGF-β1 treatment, whereas COL1A1 did not (**Figure 6B**). Next, we cultured TGF-β1-induced COL3A1+ Mp on a glass slide and performed Masson’s staining to evaluate collagens expression. Cultures treated with TGF-β1 for 96 h demonstrated significantly higher collagen volume fraction (CVF) compared to the control (**Figure 6C**).

Secondly, we assessed the mRNA expression level of pro- and anti-inflammatory cytokines in TGF-β1-induced COL3A1+ Mp. We observed a significant decrease in transcriptional levels of IL-1β, IL-6 and TNF-α and an increase in CD206 and IL-10 after exposure to TGF-β1 (**Figure 6D**). Here, we confirmed that TGF-β1 treatment induces a transition from M1 Mp to COL3A1+ Mp, which is accompanied by upregulated expression of COL3A1 and M2 Mp markers and reduced expression of pro-inflammatory cytokines.

### 3.9 COL3A1+ Mp induces adhesion and M2-polarization of Mp

**Figure 7:**
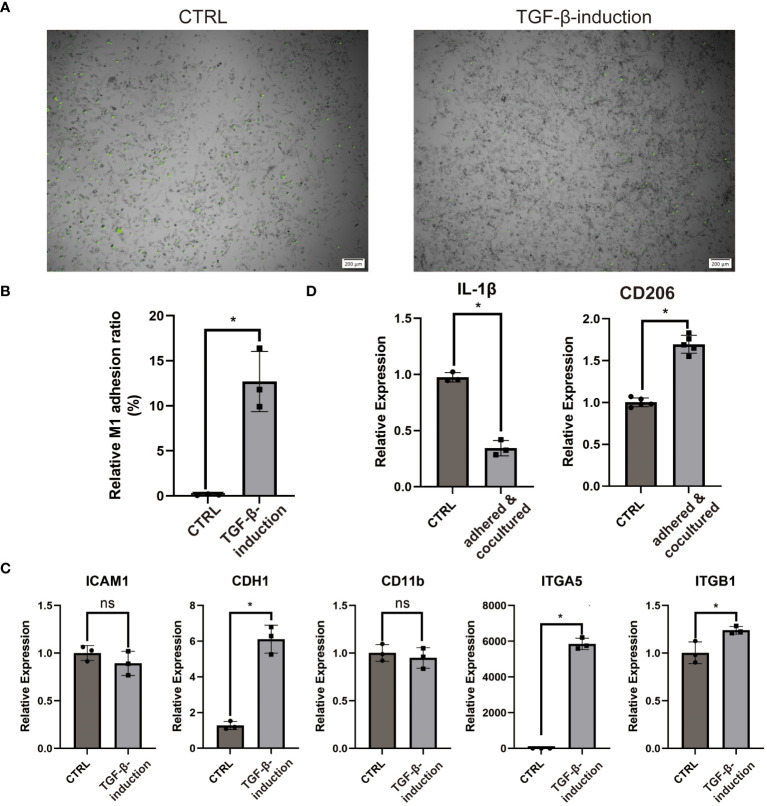
COL3A1+ Mp mediates enhanced adhesion and M2-transition of M1 Mp. **(A, B)** are representative images and quantification of M1 Mp adhesion to COL3A1+ Mp compared by the control (M1 Mp). (n = 3; *P < 0.05) **(C)** RT-qPCR analysis of adhesion molecules (ICAM1, CDH1, CD11b, ITGA5, ITGB1) in COL3A1+ Mp. (n = 3; *P < 0.05) **(D)** RT-qPCR analysis of M2 Mp marker (CD206) in M1 Mp adhered by and cocultured with COL3A1+ Mp for 48 h, with THP-1-derived M1 Mp as the control group. (n = 3; *P < 0.05). ns, not significant.

COL3A1 has been proven as an adhesive ligand for integrin α1β1 and α2β1 in fibroblasts (34, 35). Considering the expression of COL3A1 and the M2-like anti-inflammatory cytokine (IL-10), we wondered whether COL3A1+ Mp induce adhesion and M2-polarization in other Mp. Indeed, COL3A1+ Mp exhibited significantly enhanced Mp adhesion compared with M1 Mp (**Figures 7A, B**). The increased expression of adhesion molecules (CDH1, ITGA5 and ITGB1), which also conformed with KEGG analysis of scRNA-seq data, supported the upregulation of adhesion function (**Figure 7C**; **Supplementary Figure 5B**).

Next, the alternation of M1 and M2 markers expression suggested the occurrence of M2-polarization in fluorescently-labeled Mp co-cultured with COL3A1+ Mp (**Figure 7D**).

These data suggest that bone-marrow-derived COL3A1+ Mp developed enhanced Mp adhesion capability and immunosuppression effects on activated Mp.

### 3.10 COL3A1+ Mp participate in the barrier of the synovial lining

**Figure 8:**
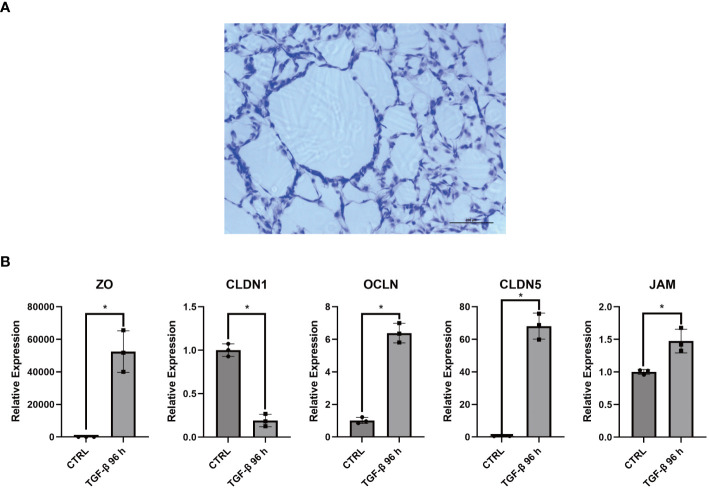
Barrier function related assays for COL3A1+ Mp. **(A)** Accidentally discovered epithelium-like structure in some replicates of COL3A1+ Mp Masson’s staining. **(B)** RNA expression of Tight junction proteins (ZO, CLDN1, OCLN, CLDN5, JAM) in COL3A1+ Mp using RT-qPCR analysis. (n = 3; *P < 0.05).

Interestingly, the multicolor immunofluorescence image reveals the membrane-like structure formed by COL3A1+ Mp in synovial sublining (**Figure 5C**). Additionally, we accidentally discovered an epithelial-like structure in some replicates of Masson’s staining (**Figure 8A**). These reminded us of the similar phenomenon in CX3CR1+ Mp reported by Culemann et al. (36). Therefore, we sought to determine the transcriptional expression level of tight junction (TJ) proteins. Here, RT-qPCR analysis showed a high level of ZO, CLDN1, OCLN, CLDN5 and JAM in COL3A1+ Mp relative to M1 Mp (**Figure 8B**). Thus, these data indicate the participation of COL3A1+ Mp in the synovial lining barrier.

## 4 Discussion

In this study, we successfully integrated synovial scRNA-seq data of Mo/Mp from different studies, identifying the existence of Mp subtypes in RA synovium. Additionally, we demonstrated the discrepant distribution of these Mo/Mp subtypes among pathotypes and their association with specific clinical disease features across the pathotypes. In addition to the previously reported Mo/Mp subtypes, we identified a specific cell type named COL3A1+ Mp, which highly expresses ECM-related and M2 Mp marker genes. Further functional characterization suggested potential roles in the formation of the physical and immunological synovial barrier of COL3A1+ Mp. As a result, our findings revealed 1) previously unreported relations between Mo/Mp subtypes and immune pathotypes, and 2) a preliminary insight into the immunological modulation function of the specific COL3A1+ Mp subtype in RA synovium.

Most subtypes of Mo/Mp identified in our integrated dataset are in accordance with previously identified Mo/Mp subtypes. CCL3+ and C1QA+ Mp reported by Wu et al. and Zhang et al. interact with and activate synovial T cells in autoimmune disease (1, 20, 37). CLEC10A+ Mp with high expression of HLA is featured by antigen-presenting function (10, 38). FOLR2+LYVE1+ Mo-Mp correlates with the remission of RA, which is in line with its known role in pro-tumor immunology via inducing immunosuppression (10, 39). IL1B+ was consistently described as a canonical pro-inflammatory M1 Mp (1, 20). NUPR1 was previously described as a participant in bone remodeling and as a marker of osteoclast (1, 25). SPP1+ Mo-Mp demonstrated a pro-inflammatory and bone-resorbing phenotype in RA synovia, but seemed to play an immunosuppression role by interacting with cancer-associated fibroblasts in particular tumor microenvironment (10, 40). The inconsistent correlation with different clinical metrics in the present study also suggests the ambiguous immunology property of SPP1+ Mo-Mp. Notably, our analysis indicated that CD52+ Mp and COL3A1+ Mp showed the most positive and negative correlations with metrics of RA activity. Interestingly, CD52+ Mp showed a significant correlation with clinical features only in the fibroid pathotype, suggesting that in the presence of fewer immune cells, the CD52+ Mp subset might exert a more pronounced role in driving RA activity.

The cell-surface CD52 molecule was previously reported to function as a promoter in T activation via cell-surface form (22). S100A9/12—another significant marker of CD52+ Mp reported here and by Alivernini et al.—has the potential to induce inflammation in fibroblast with the production of TNF-α and IL-6 and persist in remission RA, supporting our hypothesis above (10, 21).

The COL3A1+ Mp featuring a high expression of COL3A1 was not described in the studies of other public data sources (1, 10, 41). Herein, we retrieved literature related to fibroblast-like Mp and validated their existence *in vivo*.

Fibrocytes are bone marrow-derived cells that produce collagen in fibrotic tissue resulting from injury, inflammation, and aging (42). They are marked with CD34 (or CD45) and COL1 (or pro-COL1) and had been identified as promoters of auto-immune diseases, including RA (26, 42). Macrophage-myofibroblast transition (MMT) was described as the transdifferentiation process from Mp to myofibroblast-like cell (30). MMT cells also produce collagen but are characterized by the co-expression of CD68 and α-SMA (29, 30). Previous studies reported MMT cells as an inhibitor of immune responses in the contexts of cancer, tissue repair and renal fibrosis (29, 43, 44). Notably, MMT cells discovered in renal fibrosis demonstrated M2 phenotype (CD206+) (29).

However, the current study has identified COL3A1+ Mp a distinct myeloid cell subtype. It negatively correlates with RA activity and not expresses α-SMA, prompting further investigation into its the generation and biological function. Given the study’s results indicating a transition from IL1B+ Mp to COL3A1+ Mp, we hypothesized that COL3A1+ Mp represents a unique type of MMT cell. The low expression of α-SMA (ACTA2) could be contributed to the reduced mechanical stress in the lax synovial tissue compared to organs such as the heart or kidney (42).

Based on previously identified TGF-β1-induced MMT, we induced COL3A1+ Mp from THP-1-derived M1 Mp using TGF-β1 (27). The high expression of COL3A1, not COL1A1, in induced COL3A1+ Mp suggested its distinct phenotype from fibrocytes.

Similar to M2-like MMT cells reported by Haider et al., induced COL3A1+ Mp demonstrated an M1-to-M2 transition with decreased pro-inflammatory cytokines and increased anti-inflammatory cytokines production. Although rarely studied, Prieto et al. and Yin et al. still provided evidence that COL3A1 is an important mediator in the maintenance of immunosuppressive microenvironment (45, 46).

Besides, induced COL3A1+ Mp exhibited enhanced adhesion function, subsequently triggering M1-to-M2 transdifferentiation in adhered and cocultured M1 Mp. This finding could be supported by a potential combination of COL3A1-ITGA5/ITGB1 between fibroblast and Mp (35, 47).

Our study unexpectedly revealed that COL3A1 Mp contributes tissue barrier in synovial lining RA, especially in the myeloid pathotype. A similar structure in RA synovia has been described in studies by Culemann et al. and Alivernini et al., which is composed of CX3CR1+ or TREM2+ Mp (10, 36). Both studies reported physical barrier function of special Mp clusters, which contributes to the homeostasis of healthy synovium and the remission of RA synovitis. However, the DEG of the special Mp cluster in these studies didn’t include fibroblast-like markers (collagen genes or else).

Moreover, the clustering and annotation of the present study didn’t identify a cluster specifically marked by TREM2 (**Supplementary Figure 6A**). The high expression of TREM2 could be observed in multiple cell subsets (the left), though the expression in TREM2 could be found in other myeloid cell data from other datasets (the right).

Additional multicolor immunofluorescence confirmed the non-redundancy between COL3A1+ Mp, TREM2^+^ Mp and PDPN+ fibroblast *in vivo*. As shown in **Supplementary Figure 6B**, in the both myeloid and lymphoid RA synovia, COL3A1 and TREM2 marked different CD68+ Mp subtypes, neither of which was PDPN positive. In terms of barrier structure in lining layer, TREM2+ Mp structure appeared to undertake more severe disruption and dispersion compared with COL3A1+ Mp in lymphoid pathotypes. This is also aligned with the conclusion of Alivernini et al. (10).

The discrepancy of COL3A1+ Mp and TREM2+ Mp identification between the studies is probably due to the heterogeneity of RA patient recruitment and the differences in cell clustering workflows.

Based on these findings, we conclude that COL3A1 Mp mediates remission of RA via multiple mechanisms. Considering the ubiquity of myeloid cells across pathotypes, TGF-β1-induced COL3A1 Mp generation could be a potential target for treatment, promising responses in all pathotypes of RA.

The present study has potential limitations. To exploit the development and immune regulation function of COL3A1 Mp, we deployed pseudotime trajectory and deconvolution analysis. But like most bioinformatic analysis techniques, these methods are only computational models suggesting of biological meaning, necessitating further detailed biological validation. However, our functional analyses are relatively simple: 1) TGF-β1-induced COL3A1 Mp generation was not validated using knockdown-rescue experiments; 2) ScRNA-seq is required to elucidate the phenotype of induced COL3A1 Mp and compare it with COL3A1 Mp *in vivo*; 3) Animal model also could be a meaningful supplement to investigate the extent to which COL3A1 Mp affect M1 Mp and the synovial immune microenvironment entity; 4) The crosstalk between COL3A1 Mp and other Mp clusters or fibroblast, the dominant TGF-β1 producer in synovium, has not been evaluated. Nevertheless, our study provides valuable supplements for immune regulation of myeloid cells and insights into pathophysiological functions of COL3A1+ subset in RA pathotypes.

In summary, our study reveals an unexplored link between RA infiltration pathotypes and myeloid cell subsets. The identification of the particular COL3A1+ Mp subset *in vitro* and *in vivo* provides evidence for its development via macrophage-myofibroblast transition. Function assay data suggest a potential role of COL3A1+ Mp in RA remission by modulating myeloid cell infiltration and participating in lining barrier formation in the synovium. Targeting COL3A1+ Mp may hold promise for the treatment of RA with heterogeneous pathologies.

## Data availability statement

Publicly available datasets were analyzed in this study. This data can be found here: https://www.ebi.ac.uk/biostudies/arrayexpress/studies/E-MTAB-8322, EMBL-EBI, E-MTAB-8322; https://www.immport.org/shared/study/SDY998, Immport, SDY998; https://www.dev.immport.org/shared/study/SDY1599, Immport, SDY1599-SCP469.

## Ethics statement

The present study was performed in accordance with the Guidelines of the Declaration of Helsinki and approved by the institutional review board, reference number [2021]673. The studies were conducted in accordance with the local legislation and institutional requirements. The human samples used in this study were acquired from primarily isolated as part of your previous study for which ethical approval was obtained. Written informed consent for participation was not required from the participants or the participants’ legal guardians/next of kin in accordance with the national legislation and institutional requirements.

## Author contributions

XH: Conceptualization, Data curation, Formal analysis, Investigation, Methodology, Software, Validation, Visualization, Writing – original draft. ZZ: Conceptualization, Project administration, Supervision, Writing – review & editing. LL: Resources, Supervision, Writing – review & editing. MG: Writing – review & editing. WC: Supervision, Writing – review & editing. BP: Investigation, Software, Writing – review & editing. XW: Investigation, Methodology, Software, Writing – review & editing. CW: Investigation, Methodology, Writing – review & editing. CL: Investigation, Resources, Writing – review & editing. LZ: Conceptualization, Data curation, Funding acquisition, Methodology, Project administration, Software, Supervision, Writing – review & editing. PS: Conceptualization, Funding acquisition, Resources, Supervision, Writing – review & editing.

## Supplementary material

Supplementary Figure 1 Stack histogram demonstrating the ratio of Mo/Mp subtypes across datasets.

Supplementary Figure 2 Stack histograms showing the ratio of Mo/Mp subtypes and endothelial cells across datasets integrated using Atom Sketch, Harmony, or Liger.

Supplementary Figure 3 Comparison of COL3A1+ Mp/total Mp ratio between osteoarthritis and rheumatoid arthritis. **(A)** Representative dotplots; **(B)** Statistical histogram. (n = 5; *P < 0.05).

Supplementary Figure 4
*In vivo* investigation of fibrocyte and MMT cell across RA synovial pathotypes. **(A)** Representative images of immunofluorescence showing CD45+COL1A1+ and CD68+ACTA2+ cells (arrows); **(B)** Abundance comparison of CD45+COL1A1+ and CD68+ACTA2+ cells respectively across pathotypes. MMT: macrophage-myofibroblast transition.

Supplementary Figure 5 Association between COL3A1+ Mp and typical MMT. **(A)** Violin plot of scRNA-seq dataset showing the high expression of COL3A1 but not ACTA2 in COL3A1+ Mp. **(B)** KEGG analysis demonstrating top-ranked terms including “response to transforming growth factor”, “integrin binding” and “cadherin binding”.

Supplementary Figure 6 The investigation of non-redundancy between COL3A1 Mp and similar cell types. **(A)** Violin plots showing MERTK and TREM2 expression in different cell subtypes (left) or across scRNA-seq datasets (right). **(B)** Representative images of multicolor immunofluorescence showing COL3A1+ Mp (CD68+COL3A1+) (arrows), TREM2+ Mp (CD68+TREM2+) (arrows) and fibroblast (PDPN+) (arrows) in myeloid or lymphoid pathotype of RA synovia.
