# Supplementary figures and images for: Deconvolution of synovial myeloid cell subsets across pathotypes and role of COL3A1+ macrophages in rheumatoid arthritis remission
Source: Front Immunol. 2024 Mar 26;15:1307748. doi: 10.3389/fimmu.2024.1307748 (PMC11005452; doi:10.3389/fimmu.2024.1307748)

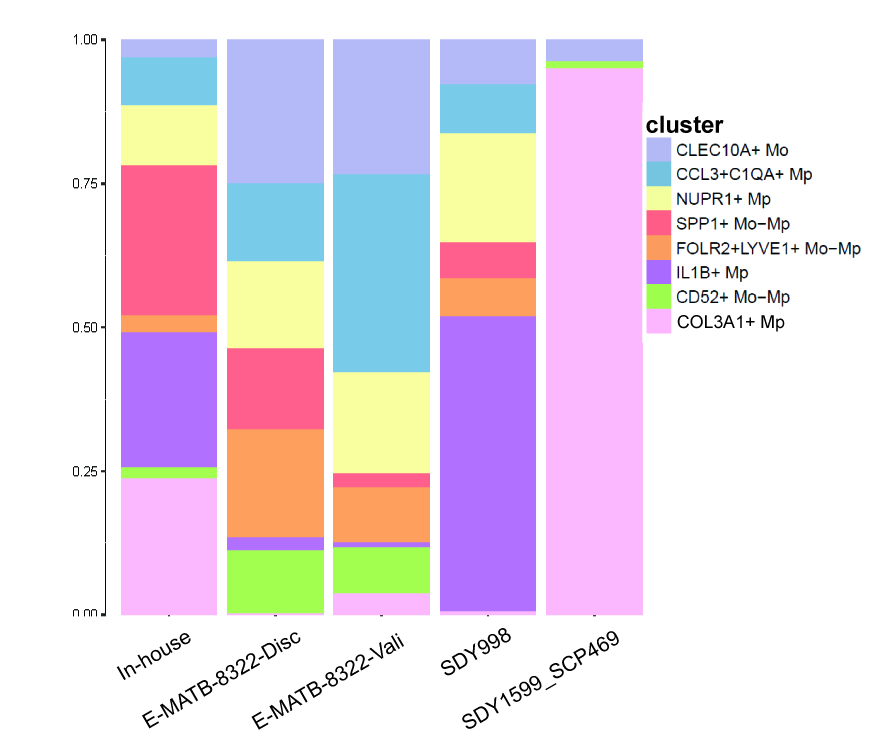

Supplement: Supplementary Figure 1 — Stack histogram demonstrating the ratio of Mo/Mp subtypes across datasets. [file Image_1.tif]

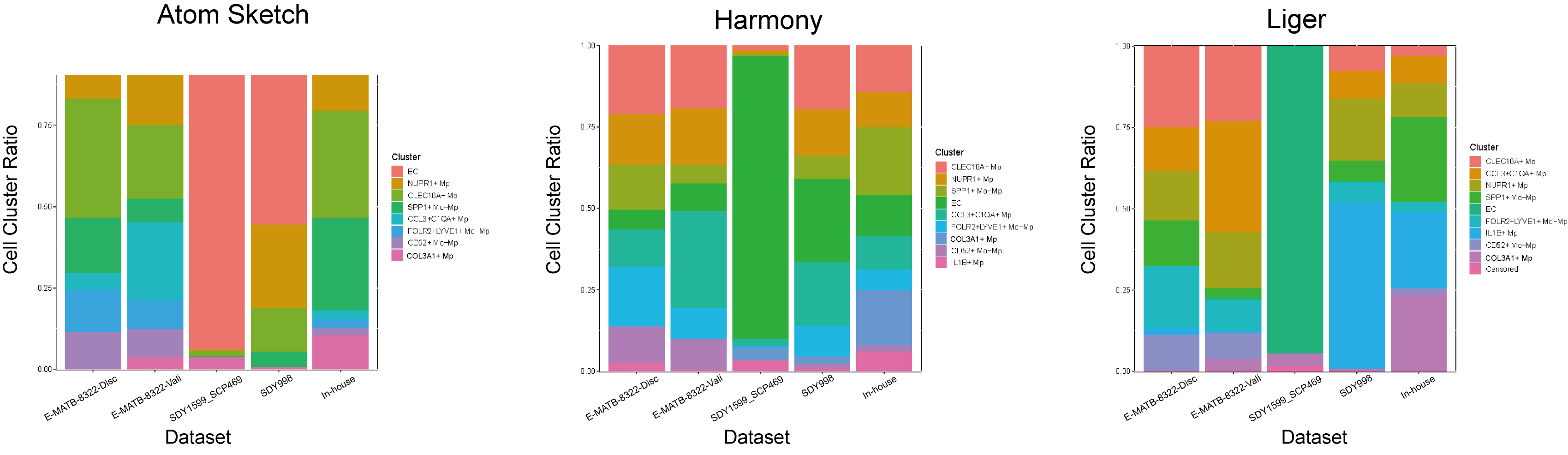

Supplement: Supplementary Figure 2 — Stack histograms showing the ratio of Mo/Mp subtypes and endothelial cells across datasets integrated using Atom Sketch, Harmony, or Liger. [file Image_2.tif]

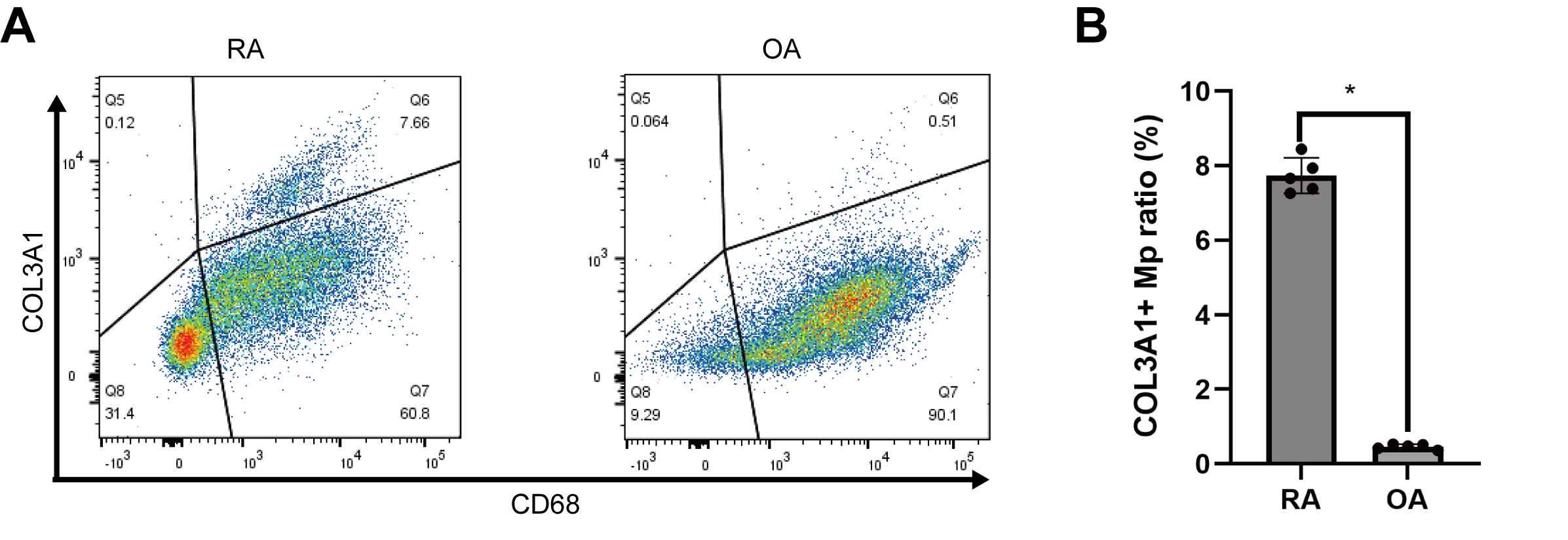

Supplement: Supplementary Figure 3 — Comparison of COL3A1+ Mp/total Mp ratio between osteoarthritis and rheumatoid arthritis. (A) Representative dotplots; (B) Statistical histogram. (n = 5; *P < 0.05). [file Image_3.tif]

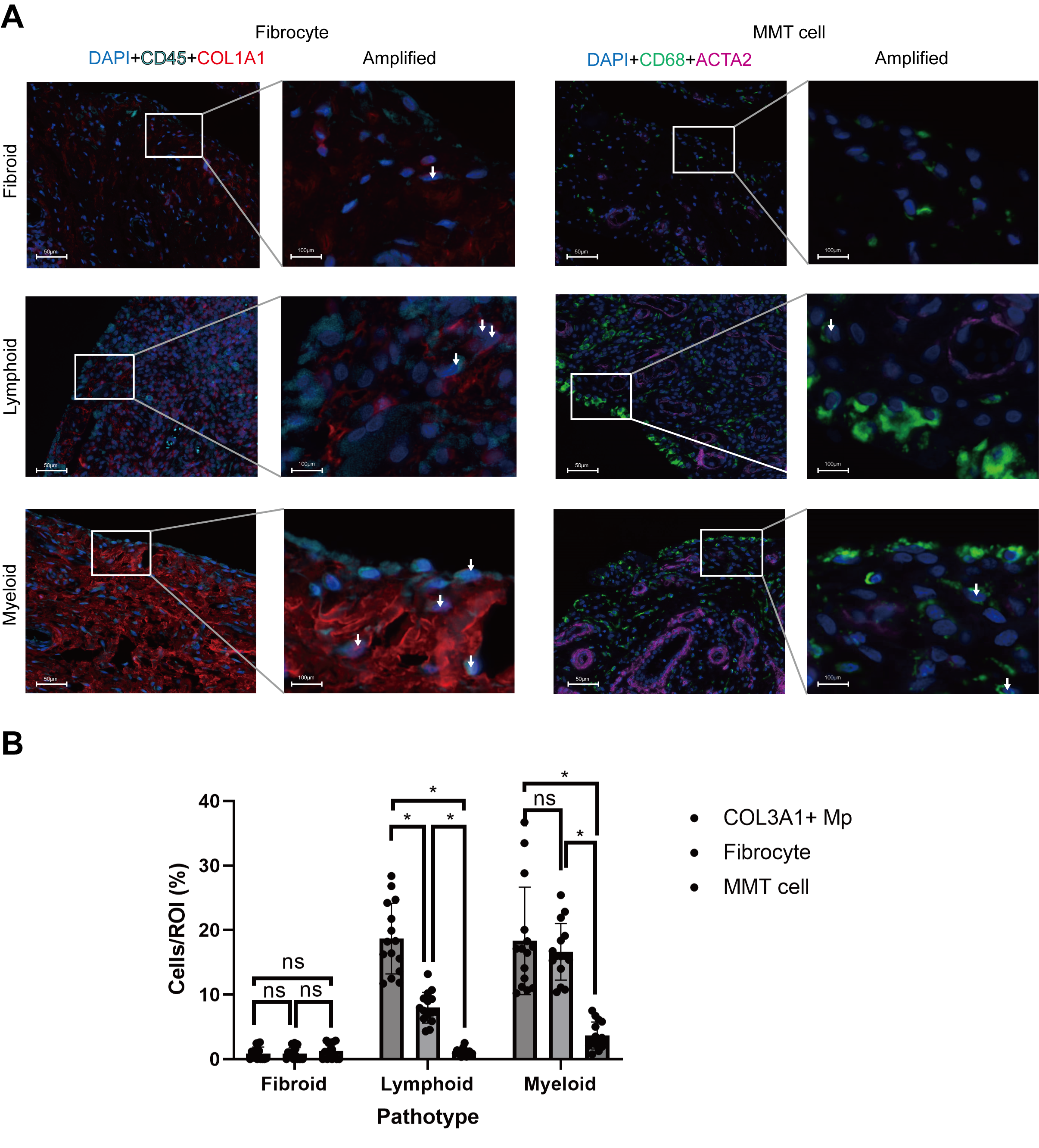

Supplement: Supplementary Figure 4 — In vivo investigation of fibrocyte and MMT cell across RA synovial pathotypes. (A) Representative images of immunofluorescence showing CD45+COL1A1+ and CD68+ACTA2+ cells (arrows); (B) Abundance comparison of CD45+COL1A1+ and CD68+ACTA2+ cells respectively across pathotypes. MMT: macrophage-myofibroblast transition. [file Image_4.tif]

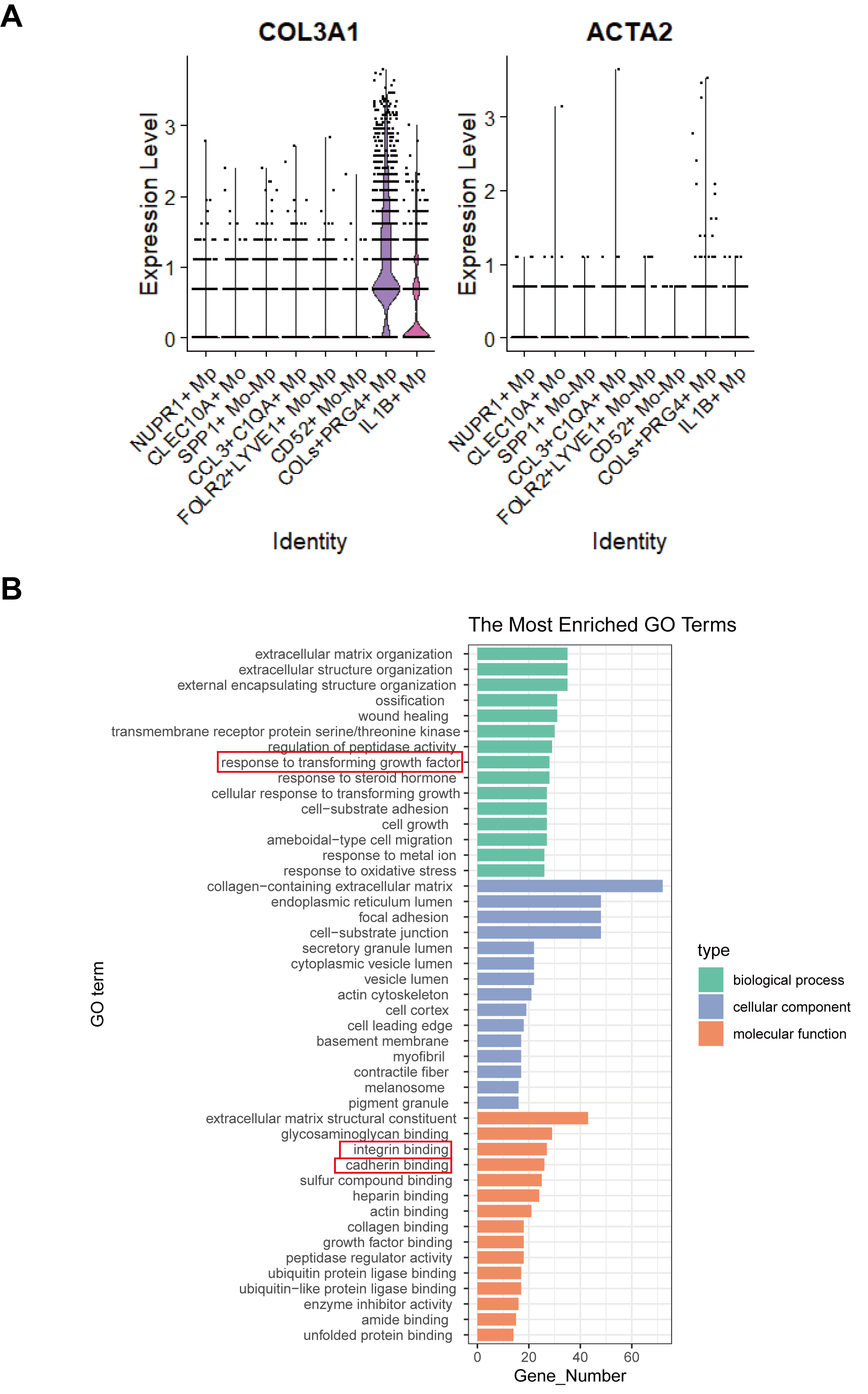

Supplement: Supplementary Figure 5 — Association between COL3A1+ Mp and typical MMT. (A) Violin plot of scRNA-seq dataset showing the high expression of COL3A1 but not ACTA2 in COL3A1+ Mp. (B) KEGG analysis demonstrating top-ranked terms including “response to transforming growth factor”, “integrin binding” and “cadherin binding”. [file Image_5.tif]

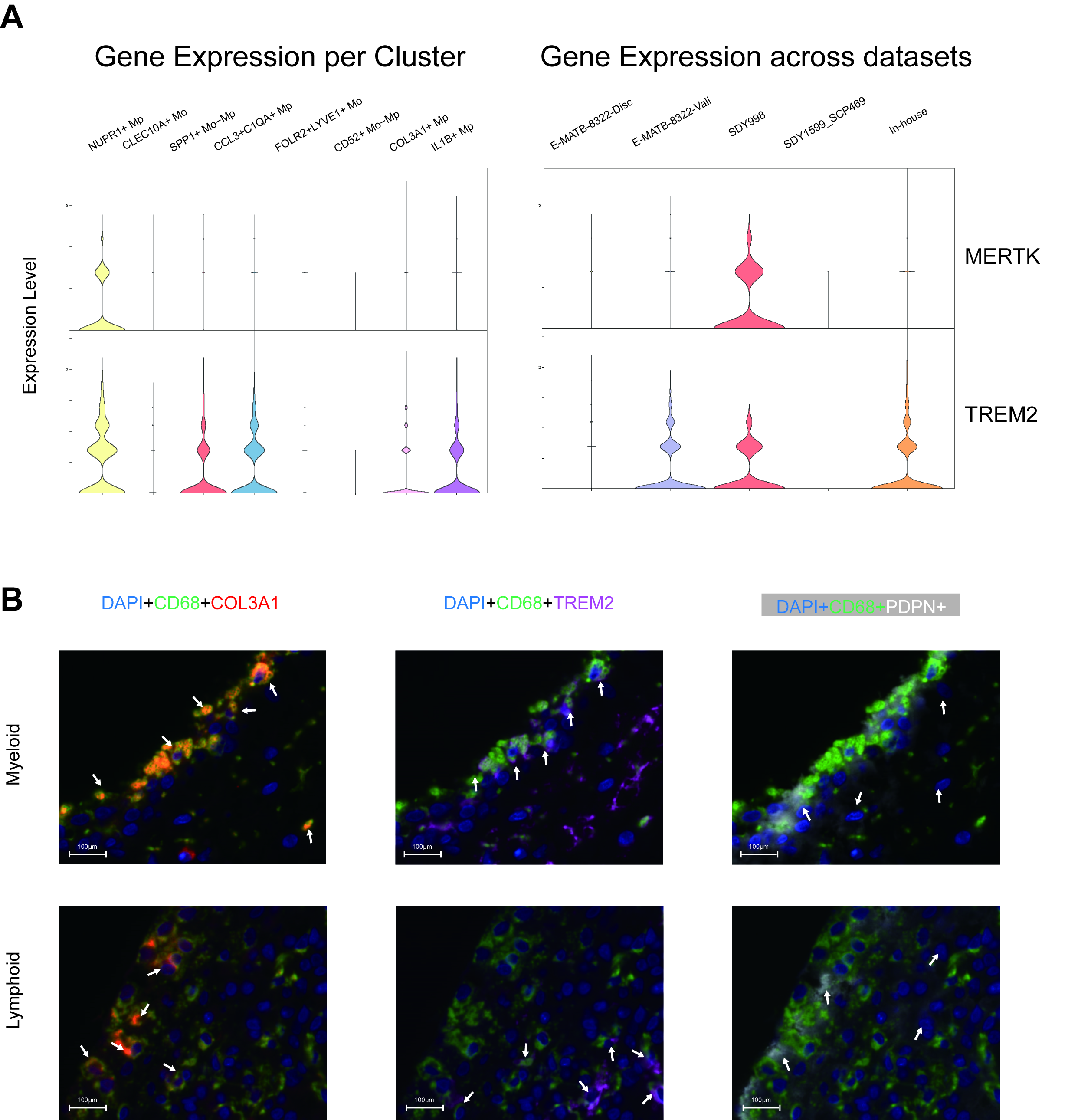

Supplement: Supplementary Figure 6 — The investigation of non-redundancy between COL3A1 Mp and similar cell types. (A) Violin plots showing MERTK and TREM2 expression in different cell subtypes (left) or across scRNA-seq datasets (right). (B) Representative images of multicolor immunofluorescence showing COL3A1+ Mp (CD68+COL3A1+) (arrows), TREM2+ Mp (CD68+TREM2+) (arrows) and fibroblast (PDPN+) (arrows) in myeloid or lymphoid pathotype of RA synovia. [file Image_6.tif]
