# Supplementary material for: Deconvolution of synovial myeloid cell subsets across pathotypes and role of COL3A1+ macrophages in rheumatoid arthritis remission
Source: Front Immunol. 2024 Mar 26;15:1307748. doi: 10.3389/fimmu.2024.1307748 (PMC11005452; doi:10.3389/fimmu.2024.1307748)
Supplement: Supplementary file 7 [file Table_1.docx]

**Table 1.** Antibodies and labels used for immunohistochemistry.

| Antibody Name | Host | Dilution | Source |
| --- | --- | --- | --- |
| CD68 | Mouse | NA | Kit-0026, Maixin, China |
| COL1A1 | Rabbit | 1:1000 | BA0325, Boster, Chiina |
| α-SMA | Rabbit | 1:1000 | AF1032, Affinity, China |
| CD206 | Rabbit | 1:1000 | 24595, CST, USA |
| Goat Anti-Rabbit IgG (HRP) | Goat | 1:4000 | ab205718, Abcam, USA |
| Goat Anti-mouse IgG (HRP) | Goat | 1:4000 | ab205719, Abcam, USA |
| FITC | NA | 1:300 | 11060, AAT Bioquest, USA |
| Cy3 | NA | 1:400 | 11065, AAT Bioquest, USA |
| Cy5 | NA | 1:400 | 11066, AAT Bioquest, USA |
| Cy7 | NA | 1:500 | 11064, AAT Bioquest, USA |

NA: Not Applicable.
