# Supplementary material for: Deconvolution of synovial myeloid cell subsets across pathotypes and role of COL3A1+ macrophages in rheumatoid arthritis remission
Source: Front Immunol. 2024 Mar 26;15:1307748. doi: 10.3389/fimmu.2024.1307748 (PMC11005452; doi:10.3389/fimmu.2024.1307748)
Supplement: Supplementary file 8 [file Table_2.docx]

**Supplementary Table 2.** Top 20 markers of each cluster in Atom Sketch integration

| cluster | gene | | | avg_log2FC | | pct.1 | | | | pct.2 | | | p_val | | | p_val_adj | |
| --- | --- | --- | --- | --- | --- | --- | --- | --- | --- | --- | --- | --- | --- | --- | --- | --- | --- |
| EC | IGFBP7 | | | 1.8480884 | | 0.8810000 | | | | 0.1490000 | | | 0.0000000 | | | 0.0000000 | |
| EC | SPARCL1 | | | 1.6108244 | | 0.8630000 | | | | 0.1450000 | | | 0.0000000 | | | 0.0000000 | |
| EC | DARC | | | 1.5538160 | | 0.6520000 | | | | 0.0060000 | | | 0.0000000 | | | 0.0000000 | |
| EC | GNG11 | | | 1.5177177 | | 0.8490000 | | | | 0.0980000 | | | 0.0000000 | | | 0.0000000 | |
| EC | PLVAP | | | 1.5062549 | | 0.7890000 | | | | 0.0170000 | | | 0.0000000 | | | 0.0000000 | |
| EC | TM4SF1 | | | 1.4780283 | | 0.8170000 | | | | 0.0440000 | | | 0.0000000 | | | 0.0000000 | |
| EC | AQP1 | | | 1.4588089 | | 0.7960000 | | | | 0.0400000 | | | 0.0000000 | | | 0.0000000 | |
| EC | RAMP2 | | | 1.4584268 | | 0.8090000 | | | | 0.0310000 | | | 0.0000000 | | | 0.0000000 | |
| EC | ECSCR | | | 1.4225961 | | 0.8090000 | | | | 0.0080000 | | | 0.0000000 | | | 0.0000000 | |
| EC | NPDC1 | | | 1.3706115 | | 0.8080000 | | | | 0.0380000 | | | 0.0000000 | | | 0.0000000 | |
| EC | CAV1 | | | 1.3485296 | | 0.8280000 | | | | 0.1250000 | | | 0.0000000 | | | 0.0000000 | |
| EC | VWF | | | 1.2885488 | | 0.7400000 | | | | 0.0200000 | | | 0.0000000 | | | 0.0000000 | |
| EC | ADIRF | | | 1.2784682 | | 0.7190000 | | | | 0.1260000 | | | 0.0000000 | | | 0.0000000 | |
| EC | CLEC14A | | | 1.2626421 | | 0.7450000 | | | | 0.0120000 | | | 0.0000000 | | | 0.0000000 | |
| EC | PTRF | | | 1.2343797 | | 0.7700000 | | | | 0.0370000 | | | 0.0000000 | | | 0.0000000 | |
| EC | CRIP2 | | | 1.2282142 | | 0.8180000 | | | | 0.1550000 | | | 0.0000000 | | | 0.0000000 | |
| EC | EGFL7 | | | 1.2100786 | | 0.7370000 | | | | 0.0560000 | | | 0.0000000 | | | 0.0000000 | |
| EC | ID1 | | | 1.1910438 | | 0.6400000 | | | | 0.0410000 | | | 0.0000000 | | | 0.0000000 | |
| EC | RAMP3 | | | 1.1738283 | | 0.6680000 | | | | 0.0170000 | | | 0.0000000 | | | 0.0000000 | |
| EC | SEPW1 | | | 1.1640139 | | 0.8330000 | | | | 0.1850000 | | | 0.0000000 | | | 0.0000000 | |
| CCL3+C1QA+ Mp | | | APOE | | 2.0040425 | | 0.8970000 | | 0.4220000 | | | 0.0000000 | | | | | 0.0000000 |
| CCL3+C1QA+ Mp | | | RGS1 | | 1.4762784 | | 0.7860000 | | 0.3300000 | | | 0.0000000 | | | | | 0.0000000 |
| CCL3+C1QA+ Mp | | | CCL3 | | 1.4097634 | | 0.7620000 | | 0.3120000 | | | 0.0000000 | | | | | 0.0000000 |
| CCL3+C1QA+ Mp | | | APOC1 | | 1.4051261 | | 0.7890000 | | 0.3120000 | | | 0.0000000 | | | | | 0.0000000 |
| CCL3+C1QA+ Mp | | | DUSP1 | | 1.2575126 | | 0.9230000 | | 0.7000000 | | | 0.0000000 | | | | | 0.0000000 |
| CCL3+C1QA+ Mp | | | FOS | | 1.2352028 | | 0.9450000 | | 0.7340000 | | | 0.0000000 | | | | | 0.0000000 |
| CCL3+C1QA+ Mp | | | RGS2 | | 1.2344158 | | 0.8030000 | | 0.3780000 | | | 0.0000000 | | | | | 0.0000000 |
| CCL3+C1QA+ Mp | | | HSPA1A | | 1.1184508 | | 0.9640000 | | 0.5860000 | | | 0.0000000 | | | | | 0.0000000 |
| CCL3+C1QA+ Mp | | | C1QB | | 1.1019911 | | 0.9490000 | | 0.5150000 | | | 0.0000000 | | | | | 0.0000000 |
| CCL3+C1QA+ Mp | | | HSPA1B | | 1.0960831 | | 0.9120000 | | 0.4420000 | | | 0.0000000 | | | | | 0.0000000 |
| CCL3+C1QA+ Mp | | | TMSB4X | | 1.0618839 | | 1.0000000 | | 0.9930000 | | | 0.0000000 | | | | | 0.0000000 |
| CCL3+C1QA+ Mp | | | C1QA | | 1.0549078 | | 0.9640000 | | 0.5450000 | | | 0.0000000 | | | | | 0.0000000 |
| CCL3+C1QA+ Mp | | | NR4A2 | | 0.9217990 | | 0.8550000 | | 0.4410000 | | | 0.0000000 | | | | | 0.0000000 |
| CCL3+C1QA+ Mp | | | JUN | | 0.9111311 | | 0.9200000 | | 0.6020000 | | | 0.0000000 | | | | | 0.0000000 |
| CCL3+C1QA+ Mp | | | GLUL | | 0.8992321 | | 0.9550000 | | 0.6600000 | | | 0.0000000 | | | | | 0.0000000 |
| CCL3+C1QA+ Mp | | | MARCO | | 0.8735229 | | 0.9170000 | | 0.4970000 | | | 0.0000000 | | | | | 0.0000000 |
| CCL3+C1QA+ Mp | | | FCGR3A | | 0.8659023 | | 0.8900000 | | 0.4360000 | | | 0.0000000 | | | | | 0.0000000 |
| CCL3+C1QA+ Mp | | | MT-CO3 | | 0.7720058 | | 0.9910000 | | 0.9430000 | | | 0.0000000 | | | | | 0.0000000 |
| CCL3+C1QA+ Mp | | | RNASE1 | | 0.7150263 | | 0.9900000 | | 0.7080000 | | | 0.0000000 | | | | | 0.0000000 |
| CCL3+C1QA+ Mp | | | NPC2 | | 0.7040548 | | 0.9970000 | | 0.9080000 | | | 0.0000000 | | | | | 0.0000000 |
| CLEC10A+ Mo | | | HLA-DPB1 | | 1.4118405 | | 0.9310000 | | 0.8290000 | | | 0.0000000 | | | | | 0.0000000 |
| CLEC10A+ Mo | | | HLA-DPA1 | | 1.2931430 | | 0.9290000 | | 0.8220000 | | | 0.0000000 | | | | | 0.0000000 |
| CLEC10A+ Mo | | | HLA-DQB1 | | 1.2318050 | | 0.8660000 | | 0.6560000 | | | 0.0000000 | | | | | 0.0000000 |
| CLEC10A+ Mo | | | HLA-DQA1 | | 1.2272495 | | 0.8550000 | | 0.5410000 | | | 0.0000000 | | | | | 0.0000000 |
| CLEC10A+ Mo | | | HLA-DRA | | 1.1850042 | | 0.9650000 | | 0.9200000 | | | 0.0000000 | | | | | 0.0000000 |
| CLEC10A+ Mo | | | GPR183 | | 1.1730427 | | 0.6640000 | | 0.2880000 | | | 0.0000000 | | | | | 0.0000000 |
| CLEC10A+ Mo | | | FCER1A | | 1.1590214 | | 0.1990000 | | 0.0120000 | | | 0.0000000 | | | | | 0.0000000 |
| CLEC10A+ Mo | | | HLA-DRB1 | | 1.1396536 | | 0.9600000 | | 0.9130000 | | | 0.0000000 | | | | | 0.0000000 |
| CLEC10A+ Mo | | | CD74 | | 0.8959862 | | 0.9950000 | | 0.9710000 | | | 0.0000000 | | | | | 0.0000000 |
| CLEC10A+ Mo | | | CLEC10A | | 0.8773136 | | 0.3990000 | | 0.0860000 | | | 0.0000000 | | | | | 0.0000000 |
| CLEC10A+ Mo | | | PLAUR | | 0.8695006 | | 0.7930000 | | 0.4970000 | | | 0.0000000 | | | | | 0.0000000 |
| CLEC10A+ Mo | | | MALAT1 | | 0.8618344 | | 0.9970000 | | 0.9890000 | | | 0.0000000 | | | | | 0.0000000 |
| CLEC10A+ Mo | | | SAT1 | | 0.7328375 | | 0.9590000 | | 0.8710000 | | | 0.0000000 | | | | | 0.0000000 |
| CLEC10A+ Mo | | | HLA-DMA | | 0.6954688 | | 0.8160000 | | 0.6350000 | | | 0.0000000 | | | | | 0.0000000 |
| CLEC10A+ Mo | | | SRGN | | 0.6250589 | | 0.9210000 | | 0.7740000 | | | 0.0000000 | | | | | 0.0000000 |
| CLEC10A+ Mo | | | UBC | | 0.6103531 | | 0.9620000 | | 0.9300000 | | | 0.0000000 | | | | | 0.0000000 |
| CLEC10A+ Mo | | | CD83 | | 0.6013813 | | 0.6830000 | | 0.3970000 | | | 0.0000000 | | | | | 0.0000000 |
| CLEC10A+ Mo | | | HLA-DMB | | 0.5361066 | | 0.6110000 | | 0.3820000 | | | 0.0000000 | | | | | 0.0000000 |
| CLEC10A+ Mo | | | CD1C | | 0.4885365 | | 0.1390000 | | 0.0060000 | | | 0.0000000 | | | | | 0.0000000 |
| CLEC10A+ Mo | | | CREM | | 0.6957682 | | 0.5190000 | | 0.2810000 | | | 4.22819148658702e-314 | | | | | 0.0000000 |
| CD52+ Mo-Mp | | | S100A9 | | 3.6823473 | | 0.9530000 | | 0.4770000 | | | 0.0000000 | | | | | 0.0000000 |
| CD52+ Mo-Mp | | | S100A8 | | 3.2997685 | | 0.9100000 | | 0.3810000 | | | 0.0000000 | | | | | 0.0000000 |
| CD52+ Mo-Mp | | | S100A12 | | 2.3069586 | | 0.6380000 | | 0.0200000 | | | 0.0000000 | | | | | 0.0000000 |
| CD52+ Mo-Mp | | | FCN1 | | 2.1805040 | | 0.9350000 | | 0.1530000 | | | 0.0000000 | | | | | 0.0000000 |
| CD52+ Mo-Mp | | | LYZ | | 1.9748043 | | 0.9430000 | | 0.6870000 | | | 0.0000000 | | | | | 0.0000000 |
| CD52+ Mo-Mp | | | CD52 | | 1.7036934 | | 0.7910000 | | 0.2040000 | | | 0.0000000 | | | | | 0.0000000 |
| CD52+ Mo-Mp | | | LST1 | | 1.4824872 | | 0.9280000 | | 0.4880000 | | | 0.0000000 | | | | | 0.0000000 |
| CD52+ Mo-Mp | | | SRGN | | 1.4272649 | | 0.9950000 | | 0.7980000 | | | 0.0000000 | | | | | 0.0000000 |
| CD52+ Mo-Mp | | | TIMP1 | | 1.3861907 | | 0.9350000 | | 0.7520000 | | | 0.0000000 | | | | | 0.0000000 |
| CD52+ Mo-Mp | | | H3F3A | | 1.2499480 | | 0.9740000 | | 0.8120000 | | | 0.0000000 | | | | | 0.0000000 |
| CD52+ Mo-Mp | | | CSTA | | 1.2196020 | | 0.7750000 | | 0.2250000 | | | 0.0000000 | | | | | 0.0000000 |
| CD52+ Mo-Mp | | | RPL39 | | 1.1190482 | | 0.9980000 | | 0.9530000 | | | 0.0000000 | | | | | 0.0000000 |
| CD52+ Mo-Mp | | | CTSS | | 1.0961746 | | 0.9590000 | | 0.7230000 | | | 0.0000000 | | | | | 0.0000000 |
| CD52+ Mo-Mp | | | H3F3B | | 1.0650463 | | 0.9980000 | | 0.9540000 | | | 0.0000000 | | | | | 0.0000000 |
| CD52+ Mo-Mp | | | RPL34 | | 1.0589888 | | 0.9990000 | | 0.9770000 | | | 0.0000000 | | | | | 0.0000000 |
| CD52+ Mo-Mp | | | NAMPT | | 1.0576342 | | 0.8790000 | | 0.4970000 | | | 0.0000000 | | | | | 0.0000000 |
| CD52+ Mo-Mp | | | RPL21 | | 1.0414706 | | 0.9860000 | | 0.9000000 | | | 0.0000000 | | | | | 0.0000000 |
| CD52+ Mo-Mp | | | MNDA | | 1.0277302 | | 0.7060000 | | 0.2810000 | | | 0.0000000 | | | | | 0.0000000 |
| CD52+ Mo-Mp | | | CORO1A | | 1.0102323 | | 0.7600000 | | 0.2310000 | | | 0.0000000 | | | | | 0.0000000 |
| CD52+ Mo-Mp | | | SERPINA1 | | 1.0098930 | | 0.7920000 | | 0.2860000 | | | 0.0000000 | | | | | 0.0000000 |
| COL3A1+ Mp | | | PRG4 | | 4.0556779 | | 0.8340000 | | 0.2660000 | | | 0.0000000 | | | | | 0.0000000 |
| COL3A1+ Mp | | | DCN | | 2.5951672 | | 0.8810000 | | 0.2070000 | | | 0.0000000 | | | | | 0.0000000 |
| COL3A1+ Mp | | | CLU | | 2.5163693 | | 0.8360000 | | 0.2850000 | | | 0.0000000 | | | | | 0.0000000 |
| COL3A1+ Mp | | | PLA2G2A | | 2.4794125 | | 0.8440000 | | 0.2300000 | | | 0.0000000 | | | | | 0.0000000 |
| COL3A1+ Mp | | | COL3A1 | | 2.2453813 | | 0.8820000 | | 0.1820000 | | | 0.0000000 | | | | | 0.0000000 |
| COL3A1+ Mp | | | COL1A2 | | 2.2442228 | | 0.9610000 | | 0.1780000 | | | 0.0000000 | | | | | 0.0000000 |
| COL3A1+ Mp | | | LUM | | 2.1524332 | | 0.9280000 | | 0.1920000 | | | 0.0000000 | | | | | 0.0000000 |
| COL3A1+ Mp | | | MGP | | 2.0594232 | | 0.9110000 | | 0.3100000 | | | 0.0000000 | | | | | 0.0000000 |
| COL3A1+ Mp | | | COL1A1 | | 1.9533328 | | 0.7960000 | | 0.1120000 | | | 0.0000000 | | | | | 0.0000000 |
| COL3A1+ Mp | | | GSN | | 1.5943793 | | 0.8920000 | | 0.5960000 | | | 0.0000000 | | | | | 0.0000000 |
| COL3A1+ Mp | | | CRTAC1 | | 1.5814060 | | 0.6550000 | | 0.1420000 | | | 0.0000000 | | | | | 0.0000000 |
| COL3A1+ Mp | | | C1S | | 1.5623041 | | 0.8750000 | | 0.1170000 | | | 0.0000000 | | | | | 0.0000000 |
| COL3A1+ Mp | | | PCOLCE | | 1.5298302 | | 0.8550000 | | 0.1110000 | | | 0.0000000 | | | | | 0.0000000 |
| COL3A1+ Mp | | | COL6A2 | | 1.5242857 | | 0.8950000 | | 0.1440000 | | | 0.0000000 | | | | | 0.0000000 |
| COL3A1+ Mp | | | HTRA1 | | 1.5121514 | | 0.8360000 | | 0.2690000 | | | 0.0000000 | | | | | 0.0000000 |
| COL3A1+ Mp | | | C1R | | 1.5055538 | | 0.7790000 | | 0.0950000 | | | 0.0000000 | | | | | 0.0000000 |
| COL3A1+ Mp | | | CCDC80 | | 1.4580744 | | 0.8140000 | | 0.0970000 | | | 0.0000000 | | | | | 0.0000000 |
| COL3A1+ Mp | | | TIMP3 | | 1.4332305 | | 0.8180000 | | 0.1980000 | | | 0.0000000 | | | | | 0.0000000 |
| COL3A1+ Mp | | | EFEMP1 | | 1.4236083 | | 0.7680000 | | 0.1070000 | | | 0.0000000 | | | | | 0.0000000 |
| COL3A1+ Mp | | | COL6A3 | | 1.4110000 | | 0.7670000 | | 0.0880000 | | | 0.0000000 | | | | | 0.0000000 |
| FOLR2+LYVE1+ Mo-Mp | | | SEPP1 | | 2.2987406 | | 0.8830000 | | 0.3410000 | | | 0.0000000 | | | | | 0.0000000 |
| FOLR2+LYVE1+ Mo-Mp | | | RNASE1 | | 1.9296308 | | 0.9900000 | | 0.7160000 | | | 0.0000000 | | | | | 0.0000000 |
| FOLR2+LYVE1+ Mo-Mp | | | HMOX1 | | 1.7268954 | | 0.8170000 | | 0.4050000 | | | 0.0000000 | | | | | 0.0000000 |
| FOLR2+LYVE1+ Mo-Mp | | | LYVE1 | | 1.6350778 | | 0.7100000 | | 0.0850000 | | | 0.0000000 | | | | | 0.0000000 |
| FOLR2+LYVE1+ Mo-Mp | | | LGMN | | 1.5617445 | | 0.9100000 | | 0.4240000 | | | 0.0000000 | | | | | 0.0000000 |
| FOLR2+LYVE1+ Mo-Mp | | | F13A1 | | 1.4734158 | | 0.7830000 | | 0.1660000 | | | 0.0000000 | | | | | 0.0000000 |
| FOLR2+LYVE1+ Mo-Mp | | | FOLR2 | | 1.2863996 | | 0.8160000 | | 0.3180000 | | | 0.0000000 | | | | | 0.0000000 |
| FOLR2+LYVE1+ Mo-Mp | | | EMP1 | | 1.2590675 | | 0.7980000 | | 0.4410000 | | | 0.0000000 | | | | | 0.0000000 |
| FOLR2+LYVE1+ Mo-Mp | | | C1QA | | 1.2334229 | | 0.9930000 | | 0.5550000 | | | 0.0000000 | | | | | 0.0000000 |
| FOLR2+LYVE1+ Mo-Mp | | | CST3 | | 1.1624018 | | 1.0000000 | | 0.9770000 | | | 0.0000000 | | | | | 0.0000000 |
| FOLR2+LYVE1+ Mo-Mp | | | SLC40A1 | | 0.9755790 | | 0.5910000 | | 0.1410000 | | | 0.0000000 | | | | | 0.0000000 |
| FOLR2+LYVE1+ Mo-Mp | | | CALM2 | | 0.9037780 | | 0.9440000 | | 0.8270000 | | | 0.0000000 | | | | | 0.0000000 |
| FOLR2+LYVE1+ Mo-Mp | | | FCGRT | | 0.8912121 | | 0.8450000 | | 0.6860000 | | | 0.0000000 | | | | | 0.0000000 |
| FOLR2+LYVE1+ Mo-Mp | | | TPT1 | | 0.8640223 | | 0.9990000 | | 0.9880000 | | | 0.0000000 | | | | | 0.0000000 |
| FOLR2+LYVE1+ Mo-Mp | | | BLVRB | | 0.8228355 | | 0.7110000 | | 0.3620000 | | | 0.0000000 | | | | | 0.0000000 |
| FOLR2+LYVE1+ Mo-Mp | | | MAMDC2 | | 0.7995625 | | 0.5030000 | | 0.0930000 | | | 0.0000000 | | | | | 0.0000000 |
| FOLR2+LYVE1+ Mo-Mp | | | MT-ND2 | | 0.9870533 | | 0.9900000 | | 0.9250000 | | | 0.0000000 | | | | | 0.0000000 |
| FOLR2+LYVE1+ Mo-Mp | | | STAB1 | | 0.9128608 | | 0.7110000 | | 0.2770000 | | | 0.0000000 | | | | | 0.0000000 |
| FOLR2+LYVE1+ Mo-Mp | | | EMB | | 0.7238449 | | 0.5610000 | | 0.1760000 | | | 0.0000000 | | | | | 0.0000000 |
| FOLR2+LYVE1+ Mo-Mp | | | DAB2 | | 0.9103816 | | 0.7840000 | | 0.4450000 | | | 0.0000000 | | | | | 0.0000000 |
| NUPR1+ Mp | | | C1QB | | 1.4171139 | | 0.9570000 | | 0.4780000 | | | 0.0000000 | | | | | 0.0000000 |
| NUPR1+ Mp | | IFI27 | | | 1.3361834 | | | 0.7170000 | | | 0.4820000 | | | 0.0000000 | 0.0000000 | | |
| NUPR1+ Mp | | C1QA | | | 1.2650926 | | | 0.9710000 | | | 0.5080000 | | | 0.0000000 | 0.0000000 | | |
| NUPR1+ Mp | | NUPR1 | | | 1.2505302 | | | 0.8320000 | | | 0.3270000 | | | 0.0000000 | 0.0000000 | | |
| NUPR1+ Mp | | FTL | | | 1.2493863 | | | 0.9930000 | | | 0.9910000 | | | 0.0000000 | 0.0000000 | | |
| NUPR1+ Mp | | S100A4 | | | 1.1862885 | | | 0.9820000 | | | 0.8470000 | | | 0.0000000 | 0.0000000 | | |
| NUPR1+ Mp | | RNASE1 | | | 1.1385492 | | | 0.9670000 | | | 0.6890000 | | | 0.0000000 | 0.0000000 | | |
| NUPR1+ Mp | | CFD | | | 1.1358287 | | | 0.9180000 | | | 0.5560000 | | | 0.0000000 | 0.0000000 | | |
| NUPR1+ Mp | | MARCO | | | 1.1283902 | | | 0.9030000 | | | 0.4650000 | | | 0.0000000 | 0.0000000 | | |
| NUPR1+ Mp | | C1QC | | | 1.1095011 | | | 0.9250000 | | | 0.4520000 | | | 0.0000000 | 0.0000000 | | |
| NUPR1+ Mp | | S100A6 | | | 1.0795624 | | | 0.9880000 | | | 0.9860000 | | | 0.0000000 | 0.0000000 | | |
| NUPR1+ Mp | | CRIP1 | | | 1.0443891 | | | 0.6950000 | | | 0.4430000 | | | 0.0000000 | 0.0000000 | | |
| NUPR1+ Mp | | APOE | | | 1.0241606 | | | 0.7710000 | | | 0.4070000 | | | 0.0000000 | 0.0000000 | | |
| NUPR1+ Mp | | EMP3 | | | 0.9564013 | | | 0.9550000 | | | 0.7250000 | | | 0.0000000 | 0.0000000 | | |
| NUPR1+ Mp | | CTSZ | | | 0.8525245 | | | 0.9120000 | | | 0.6220000 | | | 0.0000000 | 0.0000000 | | |
| NUPR1+ Mp | | TIMD4 | | | 0.8234970 | | | 0.4760000 | | | 0.0680000 | | | 0.0000000 | 0.0000000 | | |
| NUPR1+ Mp | | TMSB4X | | | 0.8131353 | | | 0.9920000 | | | 0.9940000 | | | 0.0000000 | 0.0000000 | | |
| NUPR1+ Mp | | GPNMB | | | 0.8032639 | | | 0.8220000 | | | 0.4140000 | | | 0.0000000 | 0.0000000 | | |
| NUPR1+ Mp | | NCF1 | | | 0.7368871 | | | 0.5790000 | | | 0.2570000 | | | 0.0000000 | 0.0000000 | | |
| NUPR1+ Mp | | S100A10 | | | 0.7192121 | | | 0.9800000 | | | 0.9580000 | | | 0.0000000 | 0.0000000 | | |
| SPP1+ Mo-Mp | | SPP1 | | | 2.4234018 | | | 0.7860000 | | | 0.1820000 | | | 0.0000000 | 0.0000000 | | |
| SPP1+ Mo-Mp | | MT2A | | | 1.9101305 | | | 0.7270000 | | | 0.4880000 | | | 0.0000000 | 0.0000000 | | |
| SPP1+ Mo-Mp | | GAPDH | | | 1.6790757 | | | 0.9910000 | | | 0.9330000 | | | 0.0000000 | 0.0000000 | | |
| SPP1+ Mo-Mp | | MT1X | | | 1.6588506 | | | 0.4660000 | | | 0.2570000 | | | 0.0000000 | 0.0000000 | | |
| SPP1+ Mo-Mp | | MIF | | | 1.5600684 | | | 0.8090000 | | | 0.4330000 | | | 0.0000000 | 0.0000000 | | |
| SPP1+ Mo-Mp | | C15orf48 | | | 1.3328171 | | | 0.6690000 | | | 0.2040000 | | | 0.0000000 | 0.0000000 | | |
| SPP1+ Mo-Mp | | FN1 | | | 1.3284166 | | | 0.8900000 | | | 0.6380000 | | | 0.0000000 | 0.0000000 | | |
| SPP1+ Mo-Mp | | CSTB | | | 1.3252927 | | | 0.9090000 | | | 0.7940000 | | | 0.0000000 | 0.0000000 | | |
| SPP1+ Mo-Mp | | VCAN | | | 1.2440159 | | | 0.6610000 | | | 0.2640000 | | | 0.0000000 | 0.0000000 | | |
| SPP1+ Mo-Mp | | PLIN2 | | | 1.2194273 | | | 0.6230000 | | | 0.2960000 | | | 0.0000000 | 0.0000000 | | |
| SPP1+ Mo-Mp | | TIMP1 | | | 1.1879282 | | | 0.8580000 | | | 0.7480000 | | | 0.0000000 | 0.0000000 | | |
| SPP1+ Mo-Mp | | FBP1 | | | 1.1523862 | | | 0.6460000 | | | 0.2340000 | | | 0.0000000 | 0.0000000 | | |
| SPP1+ Mo-Mp | | VIM | | | 1.0899996 | | | 0.9980000 | | | 0.9870000 | | | 0.0000000 | 0.0000000 | | |
| SPP1+ Mo-Mp | | ALDOA | | | 1.0260669 | | | 0.8120000 | | | 0.6330000 | | | 0.0000000 | 0.0000000 | | |
| SPP1+ Mo-Mp | | TPI1 | | | 1.0205411 | | | 0.8620000 | | | 0.6210000 | | | 0.0000000 | 0.0000000 | | |
| SPP1+ Mo-Mp | | ENO1 | | | 1.0172387 | | | 0.8960000 | | | 0.6630000 | | | 0.0000000 | 0.0000000 | | |
| SPP1+ Mo-Mp | | LGALS1 | | | 1.0069829 | | | 0.9710000 | | | 0.8510000 | | | 0.0000000 | 0.0000000 | | |
| SPP1+ Mo-Mp | | PKM | | | 0.9299899 | | | 0.8890000 | | | 0.6370000 | | | 0.0000000 | 0.0000000 | | |
| SPP1+ Mo-Mp | | LDHA | | | 0.9143125 | | | 0.8170000 | | | 0.6050000 | | | 0.0000000 | 0.0000000 | | |
| SPP1+ Mo-Mp | | SLC2A3 | | | 0.8752998 | | | 0.5220000 | | | 0.3190000 | | | 0.0000000 | 0.0000000 | | |

**EC:** endothelial cell

**cluster:** annotation corresponding to cluster

**gene:** gene symbol

**avg_logFC:** average log2 fold change. Positive values indicate that the gene is more highly expressed in the cluster.

**pct.1:** The percentage of cells where the gene is detected in the cluster

**pct.2:** The percentage of cells where the gene is detected on average in the other clusters

**p_val:** p-value not adjusted for multiple test correction

**p_val_adj:** Adjusted p-value, based on bonferroni correction using all genes in the dataset, used to determine significance
