# Supplementary material for: Deconvolution of synovial myeloid cell subsets across pathotypes and role of COL3A1+ macrophages in rheumatoid arthritis remission
Source: Front Immunol. 2024 Mar 26;15:1307748. doi: 10.3389/fimmu.2024.1307748 (PMC11005452; doi:10.3389/fimmu.2024.1307748)
Supplement: Supplementary file 9 [file Table_3.docx]

**Supplementary Table 3.** Top 20 markers of each cluster in Harmony integration

| cluster | gene | | | | avg_log2FC | | | | | pct.1 | | | | pct.2 | | | | p_val | | | | p_val_adj | | |
| --- | --- | --- | --- | --- | --- | --- | --- | --- | --- | --- | --- | --- | --- | --- | --- | --- | --- | --- | --- | --- | --- | --- | --- | --- |
| EC | IGFBP7 | | | | 1.5930013 | | | | | 0.7300000 | | | | 0.1590000 | | | | 0.0000000 | | | | 0.0000000 | | |
| EC | SPARCL1 | | | | 1.3834470 | | | | | 0.7150000 | | | | 0.1540000 | | | | 0.0000000 | | | | 0.0000000 | | |
| EC | DARC | | | | 1.3142094 | | | | | 0.5240000 | | | | 0.0170000 | | | | 0.0000000 | | | | 0.0000000 | | |
| EC | TM4SF1 | | | | 1.2718115 | | | | | 0.6600000 | | | | 0.0560000 | | | | 0.0000000 | | | | 0.0000000 | | |
| EC | GNG11 | | | | 1.2620691 | | | | | 0.6910000 | | | | 0.1110000 | | | | 0.0000000 | | | | 0.0000000 | | |
| EC | PLVAP | | | | 1.2503845 | | | | | 0.6340000 | | | | 0.0300000 | | | | 0.0000000 | | | | 0.0000000 | | |
| EC | AQP1 | | | | 1.2399257 | | | | | 0.6460000 | | | | 0.0480000 | | | | 0.0000000 | | | | 0.0000000 | | |
| EC | RAMP2 | | | | 1.2074980 | | | | | 0.6490000 | | | | 0.0430000 | | | | 0.0000000 | | | | 0.0000000 | | |
| EC | ECSCR | | | | 1.1612206 | | | | | 0.6380000 | | | | 0.0250000 | | | | 0.0000000 | | | | 0.0000000 | | |
| EC | ADIRF | | | | 1.1605381 | | | | | 0.6140000 | | | | 0.1250000 | | | | 0.0000000 | | | | 0.0000000 | | |
| EC | NPDC1 | | | | 1.1478775 | | | | | 0.6560000 | | | | 0.0490000 | | | | 0.0000000 | | | | 0.0000000 | | |
| EC | CAV1 | | | | 1.1181119 | | | | | 0.6760000 | | | | 0.1340000 | | | | 0.0000000 | | | | 0.0000000 | | |
| EC | VWF | | | | 1.0576546 | | | | | 0.5950000 | | | | 0.0320000 | | | | 0.0000000 | | | | 0.0000000 | | |
| EC | CLEC14A | | | | 1.0302471 | | | | | 0.5970000 | | | | 0.0240000 | | | | 0.0000000 | | | | 0.0000000 | | |
| EC | PTRF | | | | 1.0282010 | | | | | 0.6200000 | | | | 0.0430000 | | | | 0.0000000 | | | | 0.0000000 | | |
| EC | CRIP2 | | | | 1.0197486 | | | | | 0.6910000 | | | | 0.1620000 | | | | 0.0000000 | | | | 0.0000000 | | |
| EC | ID1 | | | | 1.0050965 | | | | | 0.5240000 | | | | 0.0460000 | | | | 0.0000000 | | | | 0.0000000 | | |
| EC | RAMP3 | | | | 0.9741685 | | | | | 0.5400000 | | | | 0.0260000 | | | | 0.0000000 | | | | 0.0000000 | | |
| EC | EGFL7 | | | | 0.9704970 | | | | | 0.5890000 | | | | 0.0720000 | | | | 0.0000000 | | | | 0.0000000 | | |
| EC | IGFBP4 | | | | 0.9452324 | | | | | 0.6660000 | | | | 0.1910000 | | | | 0.0000000 | | | | 0.0000000 | | |
| CCL3+C1QA+ Mp | | | APOE | | | | 1.7809613 | | | | 0.8500000 | | | | | | 0.3930000 | | | 0.0000000 | | | 0.0000000 | |
| CCL3+C1QA+ Mp | | | | C1QB | | | | 1.4162648 | | | | 0.9660000 | | | 0.4830000 | | | | 0.0000000 | | | | | 0.0000000 |
| CCL3+C1QA+ Mp | | | | C1QA | | | | 1.3814197 | | | | 0.9740000 | | | 0.5140000 | | | | 0.0000000 | | | | | 0.0000000 |
| CCL3+C1QA+ Mp | | | | IFI27 | | | | 1.2628400 | | | | 0.7060000 | | | 0.4840000 | | | | 0.0000000 | | | | | 0.0000000 |
| CCL3+C1QA+ Mp | | | | C1QC | | | | 1.1433889 | | | | 0.9410000 | | | 0.4560000 | | | | 0.0000000 | | | | | 0.0000000 |
| CCL3+C1QA+ Mp | | | | APOC1 | | | | 1.0159162 | | | | 0.6890000 | | | 0.2960000 | | | | 0.0000000 | | | | | 0.0000000 |
| CCL3+C1QA+ Mp | | | | CCL3 | | | | 1.0050798 | | | | 0.6530000 | | | 0.3010000 | | | | 0.0000000 | | | | | 0.0000000 |
| CCL3+C1QA+ Mp | | | | RGS1 | | | | 0.9983089 | | | | 0.6810000 | | | 0.3170000 | | | | 0.0000000 | | | | | 0.0000000 |
| CCL3+C1QA+ Mp | | | | FOS | | | | 0.9610600 | | | | 0.9210000 | | | 0.7220000 | | | | 0.0000000 | | | | | 0.0000000 |
| CCL3+C1QA+ Mp | | | | RNASE1 | | | | 0.8972304 | | | | 0.9670000 | | | 0.6960000 | | | | 0.0000000 | | | | | 0.0000000 |
| CCL3+C1QA+ Mp | | | | NUPR1 | | | | 0.8764016 | | | | 0.7230000 | | | 0.3490000 | | | | 0.0000000 | | | | | 0.0000000 |
| CCL3+C1QA+ Mp | | | | DUSP1 | | | | 0.8637054 | | | | 0.8670000 | | | 0.6940000 | | | | 0.0000000 | | | | | 0.0000000 |
| CCL3+C1QA+ Mp | | | | JUN | | | | 0.8187829 | | | | 0.8600000 | | | 0.5890000 | | | | 0.0000000 | | | | | 0.0000000 |
| CCL3+C1QA+ Mp | | | | CFD | | | | 0.8186459 | | | | 0.9040000 | | | 0.5600000 | | | | 0.0000000 | | | | | 0.0000000 |
| CCL3+C1QA+ Mp | | | | PLTP | | | | 0.8033946 | | | | 0.7850000 | | | 0.3740000 | | | | 0.0000000 | | | | | 0.0000000 |
| CCL3+C1QA+ Mp | | | | HLA-DQA1 | | | | 0.7863144 | | | | 0.9310000 | | | 0.5630000 | | | | 0.0000000 | | | | | 0.0000000 |
| CCL3+C1QA+ Mp | | | | GLUL | | | | 0.7470859 | | | | 0.9140000 | | | 0.6490000 | | | | 0.0000000 | | | | | 0.0000000 |
| CCL3+C1QA+ Mp | | | | HLA-DRA | | | | 0.7388863 | | | | 0.9960000 | | | 0.9210000 | | | | 0.0000000 | | | | | 0.0000000 |
| CCL3+C1QA+ Mp | | | | CD74 | | | | 0.7237158 | | | | 0.9990000 | | | 0.9750000 | | | | 0.0000000 | | | | | 0.0000000 |
| CCL3+C1QA+ Mp | | | | FCGR3A | | | | 0.7214197 | | | | 0.8010000 | | | 0.4230000 | | | | 0.0000000 | | | | | 0.0000000 |
| CD52+ Mo-Mp | | | | S100A9 | | | | 3.598623 | | | | 0.932 | | | 0.479 | | | | 0 | | | | | 0 |
| CD52+ Mo-Mp | | | | S100A8 | | | | 3.227757 | | | | 0.886 | | | 0.384 | | | | 0 | | | | | 0 |
| CD52+ Mo-Mp | | | | S100A12 | | | | 2.284383 | | | | 0.614 | | | 0.022 | | | | 0 | | | | | 0 |
| CD52+ Mo-Mp | | | | FCN1 | | | | 2.152613 | | | | 0.922 | | | 0.155 | | | | 0 | | | | | 0 |
| CD52+ Mo-Mp | | | | LYZ | | | | 1.933745 | | | | 0.939 | | | 0.689 | | | | 0 | | | | | 0 |
| CD52+ Mo-Mp | | | | CD52 | | | | 1.685973 | | | | 0.791 | | | 0.205 | | | | 0 | | | | | 0 |
| CD52+ Mo-Mp | | | | LST1 | | | | 1.507289 | | | | 0.933 | | | 0.489 | | | | 0 | | | | | 0 |
| CD52+ Mo-Mp | | | | SRGN | | | | 1.360673 | | | | 0.996 | | | 0.801 | | | | 0 | | | | | 0 |
| CD52+ Mo-Mp | | | | TIMP1 | | | | 1.352024 | | | | 0.926 | | | 0.752 | | | | 0 | | | | | 0 |
| CD52+ Mo-Mp | | | | H3F3A | | | | 1.246563 | | | | 0.975 | | | 0.811 | | | | 0 | | | | | 0 |
| CD52+ Mo-Mp | | | | CSTA | | | | 1.22605 | | | | 0.78 | | | 0.226 | | | | 0 | | | | | 0 |
| CD52+ Mo-Mp | | | | RPL39 | | | | 1.118756 | | | | 0.994 | | | 0.953 | | | | 0 | | | | | 0 |
| CD52+ Mo-Mp | | | | CTSS | | | | 1.102024 | | | | 0.964 | | | 0.725 | | | | 0 | | | | | 0 |
| CD52+ Mo-Mp | | | | RPL34 | | | | 1.088798 | | | | 0.999 | | | 0.977 | | | | 0 | | | | | 0 |
| CD52+ Mo-Mp | | | | RPL21 | | | | 1.08075 | | | | 0.983 | | | 0.899 | | | | 0 | | | | | 0 |
| CD52+ Mo-Mp | | | | H3F3B | | | | 1.052179 | | | | 0.998 | | | 0.953 | | | | 0 | | | | | 0 |
| CD52+ Mo-Mp | | | | NAMPT | | | | 1.042473 | | | | 0.885 | | | 0.498 | | | | 0 | | | | | 0 |
| CD52+ Mo-Mp | | | | MNDA | | | | 1.025197 | | | | 0.716 | | | 0.281 | | | | 0 | | | | | 0 |
| CD52+ Mo-Mp | | | | CORO1A | | | | 0.996103 | | | | 0.752 | | | 0.233 | | | | 0 | | | | | 0 |
| CD52+ Mo-Mp | | | | RPL26 | | | | 0.988897 | | | | 0.98 | | | 0.862 | | | | 0 | | | | | 0 |
| CLEC10A+ Mo | | | | FCER1A | | | | 1.5542778 | | | | 0.3020000 | | | 0.0130000 | | | | 0.0000000 | | | | | 0.0000000 |
| CLEC10A+ Mo | | | | HLA-DPB1 | | | | 1.5220055 | | | | 0.9830000 | | | 0.8330000 | | | | 0.0000000 | | | | | 0.0000000 |
| CLEC10A+ Mo | | | | HLA-DPA1 | | | | 1.3437114 | | | | 0.9780000 | | | 0.8270000 | | | | 0.0000000 | | | | | 0.0000000 |
| CLEC10A+ Mo | | | | GPR183 | | | | 1.2951263 | | | | 0.7670000 | | | 0.3110000 | | | | 0.0000000 | | | | | 0.0000000 |
| CLEC10A+ Mo | | | | HLA-DQA1 | | | | 1.2357120 | | | | 0.8970000 | | | 0.5690000 | | | | 0.0000000 | | | | | 0.0000000 |
| CLEC10A+ Mo | | | | HLA-DQB1 | | | | 1.2342233 | | | | 0.9220000 | | | 0.6720000 | | | | 0.0000000 | | | | | 0.0000000 |
| CLEC10A+ Mo | | | | HLA-DRA | | | | 1.2263336 | | | | 0.9920000 | | | 0.9220000 | | | | 0.0000000 | | | | | 0.0000000 |
| CLEC10A+ Mo | | | | HLA-DRB1 | | | | 1.1733398 | | | | 0.9860000 | | | 0.9150000 | | | | 0.0000000 | | | | | 0.0000000 |
| CLEC10A+ Mo | | | | CLEC10A | | | | 1.1658070 | | | | 0.5620000 | | | 0.0910000 | | | | 0.0000000 | | | | | 0.0000000 |
| CLEC10A+ Mo | | | | AREG | | | | 1.0546030 | | | | 0.3810000 | | | 0.0960000 | | | | 0.0000000 | | | | | 0.0000000 |
| CLEC10A+ Mo | | | | LYZ | | | | 1.0135950 | | | | 0.9270000 | | | 0.6630000 | | | | 0.0000000 | | | | | 0.0000000 |
| CLEC10A+ Mo | | | | CXCR4 | | | | 0.9842548 | | | | 0.6620000 | | | 0.2980000 | | | | 0.0000000 | | | | | 0.0000000 |
| CLEC10A+ Mo | | | | RPS2 | | | | 0.9396680 | | | | 0.9640000 | | | 0.9240000 | | | | 0.0000000 | | | | | 0.0000000 |
| CLEC10A+ Mo | | | | CREM | | | | 0.9394572 | | | | 0.6090000 | | | 0.2920000 | | | | 0.0000000 | | | | | 0.0000000 |
| CLEC10A+ Mo | | | | RGS2 | | | | 0.9230998 | | | | 0.7160000 | | | 0.3630000 | | | | 0.0000000 | | | | | 0.0000000 |
| CLEC10A+ Mo | | | | SRGN | | | | 0.9020465 | | | | 0.9740000 | | | 0.7830000 | | | | 0.0000000 | | | | | 0.0000000 |
| CLEC10A+ Mo | | | | CD74 | | | | 0.8371845 | | | | 0.9970000 | | | 0.9760000 | | | | 0.0000000 | | | | | 0.0000000 |
| CLEC10A+ Mo | | | | PLAUR | | | | 0.7972481 | | | | 0.8190000 | | | 0.5270000 | | | | 0.0000000 | | | | | 0.0000000 |
| CLEC10A+ Mo | | | | RPL10 | | | | 0.7746318 | | | | 0.9660000 | | | 0.9750000 | | | | 0.0000000 | | | | | 0.0000000 |
| CLEC10A+ Mo | | | | FCN1 | | | | 0.7620693 | | | | 0.4950000 | | | 0.1470000 | | | | 0.0000000 | | | | | 0.0000000 |
| COL3A1+ Mp | | | | PRG4 | | | | 3.8389177 | | | | 0.8790000 | | | 0.2570000 | | | | 0.0000000 | | | | | 0.0000000 |
| COL3A1+ Mp | | | | MT-RNR1 | | | | 2.3761390 | | | | 0.7940000 | | | 0.2200000 | | | | 0.0000000 | | | | | 0.0000000 |
| COL3A1+ Mp | | | | MT-RNR2 | | | | 2.3623987 | | | | 0.7960000 | | | 0.2290000 | | | | 0.0000000 | | | | | 0.0000000 |
| COL3A1+ Mp | | | | COL3A1 | | | | 1.9445645 | | | | 0.9090000 | | | 0.1690000 | | | | 0.0000000 | | | | | 0.0000000 |
| COL3A1+ Mp | | | | COL1A1 | | | | 1.8556845 | | | | 0.8730000 | | | 0.0960000 | | | | 0.0000000 | | | | | 0.0000000 |
| COL3A1+ Mp | | | | FN1 | | | | 1.8506344 | | | | 0.9810000 | | | 0.6520000 | | | | 0.0000000 | | | | | 0.0000000 |
| COL3A1+ Mp | | | | COL1A2 | | | | 1.8455165 | | | | 0.9540000 | | | 0.1660000 | | | | 0.0000000 | | | | | 0.0000000 |
| COL3A1+ Mp | | | | CLU | | | | 1.7300445 | | | | 0.8590000 | | | 0.2770000 | | | | 0.0000000 | | | | | 0.0000000 |
| COL3A1+ Mp | | | | PLA2G2A | | | | 1.5455850 | | | | 0.8850000 | | | 0.2200000 | | | | 0.0000000 | | | | | 0.0000000 |
| COL3A1+ Mp | | | | DCN | | | | 1.4217504 | | | | 0.8880000 | | | 0.1980000 | | | | 0.0000000 | | | | | 0.0000000 |
| COL3A1+ Mp | | | | MGP | | | | 1.3623906 | | | | 0.9100000 | | | 0.3020000 | | | | 0.0000000 | | | | | 0.0000000 |
| COL3A1+ Mp | | | | CLEC3B | | | | 1.3315135 | | | | 0.7570000 | | | 0.0890000 | | | | 0.0000000 | | | | | 0.0000000 |
| COL3A1+ Mp | | | | LUM | | | | 1.3139687 | | | | 0.9110000 | | | 0.1830000 | | | | 0.0000000 | | | | | 0.0000000 |
| COL3A1+ Mp | | | | CRTAC1 | | | | 1.3135179 | | | | 0.8050000 | | | 0.1280000 | | | | 0.0000000 | | | | | 0.0000000 |
| COL3A1+ Mp | | | | HTRA1 | | | | 1.2992664 | | | | 0.8830000 | | | 0.2590000 | | | | 0.0000000 | | | | | 0.0000000 |
| COL3A1+ Mp | | | | COL6A3 | | | | 1.2852802 | | | | 0.8340000 | | | 0.0730000 | | | | 0.0000000 | | | | | 0.0000000 |
| COL3A1+ Mp | | | | COL6A2 | | | | 1.2452869 | | | | 0.9030000 | | | 0.1300000 | | | | 0.0000000 | | | | | 0.0000000 |
| COL3A1+ Mp | | | | BGN | | | | 1.1778811 | | | | 0.7530000 | | | 0.0890000 | | | | 0.0000000 | | | | | 0.0000000 |
| COL3A1+ Mp | | | | PCOLCE | | | | 1.1749206 | | | | 0.8600000 | | | 0.0970000 | | | | 0.0000000 | | | | | 0.0000000 |
| COL3A1+ Mp | | | | TIMP3 | | | | 1.1571784 | | | | 0.8510000 | | | 0.1850000 | | | | 0.0000000 | | | | | 0.0000000 |
| FOLR2+LYVE1+ Mo-Mp | | SEPP1 | | | | 1.9873785 | | | 0.7940000 | | | | 0.3320000 | | | 0.0000000 | | | | | 0.0000000 | | | |
| FOLR2+LYVE1+ Mo-Mp | | HMOX1 | | | | 1.7785550 | | | 0.7930000 | | | | 0.3980000 | | | 0.0000000 | | | | | 0.0000000 | | | |
| FOLR2+LYVE1+ Mo-Mp | | RNASE1 | | | | 1.5113658 | | | 0.9710000 | | | | 0.7140000 | | | 0.0000000 | | | | | 0.0000000 | | | |
| FOLR2+LYVE1+ Mo-Mp | | LYVE1 | | | | 1.3450418 | | | 0.5600000 | | | | 0.0840000 | | | 0.0000000 | | | | | 0.0000000 | | | |
| FOLR2+LYVE1+ Mo-Mp | | LGMN | | | | 1.2560821 | | | 0.8240000 | | | | 0.4220000 | | | 0.0000000 | | | | | 0.0000000 | | | |
| FOLR2+LYVE1+ Mo-Mp | | F13A1 | | | | 1.1607881 | | | 0.6630000 | | | | 0.1620000 | | | 0.0000000 | | | | | 0.0000000 | | | |
| FOLR2+LYVE1+ Mo-Mp | | C1QA | | | | 1.0653714 | | | 0.9760000 | | | | 0.5460000 | | | 0.0000000 | | | | | 0.0000000 | | | |
| FOLR2+LYVE1+ Mo-Mp | | EMP1 | | | | 1.0363914 | | | 0.7260000 | | | | 0.4390000 | | | 0.0000000 | | | | | 0.0000000 | | | |
| FOLR2+LYVE1+ Mo-Mp | | FOLR2 | | | | 1.0242765 | | | 0.7350000 | | | | 0.3140000 | | | 0.0000000 | | | | | 0.0000000 | | | |
| FOLR2+LYVE1+ Mo-Mp | | MT-ND2 | | | | 0.9683860 | | | 0.9880000 | | | | 0.9230000 | | | 0.0000000 | | | | | 0.0000000 | | | |
| FOLR2+LYVE1+ Mo-Mp | | C1QC | | | | 0.9108392 | | | 0.9230000 | | | | 0.4910000 | | | 0.0000000 | | | | | 0.0000000 | | | |
| FOLR2+LYVE1+ Mo-Mp | | MT-ND1 | | | | 0.8829710 | | | 0.9880000 | | | | 0.9260000 | | | 0.0000000 | | | | | 0.0000000 | | | |
| FOLR2+LYVE1+ Mo-Mp | | C1QB | | | | 0.6796504 | | | 0.9420000 | | | | 0.5190000 | | | 0.0000000 | | | | | 0.0000000 | | | |
| FOLR2+LYVE1+ Mo-Mp | | TPT1 | | | | 0.7406547 | | | 0.9990000 | | | | 0.9870000 | | | 5.08479220553632e-317 | | | | | 1.24231643165663e-312 | | | |
| FOLR2+LYVE1+ Mo-Mp | | CST3 | | | | 0.9592101 | | | 0.9990000 | | | | 0.9770000 | | | 9.89516222410386e-315 | | | | | 2.41758603459305e-310 | | | |
| FOLR2+LYVE1+ Mo-Mp | | STAB1 | | | | 0.8061864 | | | 0.6530000 | | | | 0.2740000 | | | 0.0000000 | | | | | 0.0000000 | | | |
| FOLR2+LYVE1+ Mo-Mp | | BLVRB | | | | 0.6770753 | | | 0.6290000 | | | | 0.3620000 | | | 0.0000000 | | | | | 0.0000000 | | | |
| FOLR2+LYVE1+ Mo-Mp | | MAMDC2 | | | | 0.6287691 | | | 0.3970000 | | | | 0.0930000 | | | 0.0000000 | | | | | 0.0000000 | | | |
| FOLR2+LYVE1+ Mo-Mp | | MT-ND3 | | | | 0.9031167 | | | 0.9780000 | | | | 0.9060000 | | | 0.0000000 | | | | | 0.0000000 | | | |
| FOLR2+LYVE1+ Mo-Mp | | PLTP | | | | 0.9707517 | | | 0.7640000 | | | | 0.4040000 | | | 0.0000000 | | | | | 0.0000000 | | | |
| IL1B+ Mp | | NEAT1 | | | | 2.3033835 | | | 0.9930000 | | | | 0.9520000 | | | 0.0000000 | | | | | 0.0000000 | | | |
| IL1B+ Mp | | HNRNPA2B1 | | | | 1.0958905 | | | 0.7830000 | | | | 0.6690000 | | | 0.0000000 | | | | | 0.0000000 | | | |
| IL1B+ Mp | | XIST | | | | 1.0335826 | | | 0.5540000 | | | | 0.4090000 | | | 0.0000000 | | | | | 0.0000000 | | | |
| IL1B+ Mp | | RSRP1 | | | | 0.9255664 | | | 0.4630000 | | | | 0.2180000 | | | 0.0000000 | | | | | 0.0000000 | | | |
| IL1B+ Mp | | ZEB2 | | | | 1.0502574 | | | 0.6450000 | | | | 0.4310000 | | | 0.0000000 | | | | | 0.0000000 | | | |
| IL1B+ Mp | | CCNL1 | | | | 0.9118165 | | | 0.6540000 | | | | 0.4500000 | | | 0.0000000 | | | | | 0.0000000 | | | |
| IL1B+ Mp | | NFKB1 | | | | 0.9467288 | | | 0.3510000 | | | | 0.2190000 | | | 0.0000000 | | | | | 0.0000000 | | | |
| IL1B+ Mp | | MCL1 | | | | 1.1109812 | | | 0.7010000 | | | | 0.6160000 | | | 0.0000000 | | | | | 0.0000000 | | | |
| IL1B+ Mp | | HNRNPU | | | | 1.0167329 | | | 0.6700000 | | | | 0.4840000 | | | 0.0000000 | | | | | 0.0000000 | | | |
| IL1B+ Mp | | FCGR2C | | | | 1.0597087 | | | 0.3420000 | | | | 0.0470000 | | | 0.0000000 | | | | | 0.0000000 | | | |
| IL1B+ Mp | | QKI | | | | 0.6146755 | | | 0.4420000 | | | | 0.3020000 | | | 0.0000000 | | | | | 0.0000000 | | | |
| IL1B+ Mp | | NFKBIZ | | | | 1.2336330 | | | 0.5220000 | | | | 0.2650000 | | | 0.0000000 | | | | | 0.0000000 | | | |
| IL1B+ Mp | | CCN2 | | | | 0.4270670 | | | 0.4330000 | | | | 0.0570000 | | | 0.0000000 | | | | | 0.0000000 | | | |
| IL1B+ Mp | | PDE4DIP | | | | 0.7408814 | | | 0.3730000 | | | | 0.1970000 | | | 0.0000000 | | | | | 0.0000000 | | | |
| IL1B+ Mp | | HSPH1 | | | | 1.2048301 | | | 0.4280000 | | | | 0.3780000 | | | 0.0000000 | | | | | 0.0000000 | | | |
| IL1B+ Mp | | CFLAR | | | | 0.7114578 | | | 0.5290000 | | | | 0.3140000 | | | 0.0000000 | | | | | 0.0000000 | | | |
| IL1B+ Mp | | SELENOM | | | | 0.4200926 | | | 0.4170000 | | | | 0.0660000 | | | 0.0000000 | | | | | 0.0000000 | | | |
| IL1B+ Mp | | IGFBP5 | | | | 0.4081815 | | | 0.4780000 | | | | 0.1020000 | | | 0.0000000 | | | | | 0.0000000 | | | |
| IL1B+ Mp | | SPARCL1 | | | | 0.4669541 | | | 0.6950000 | | | | 0.2770000 | | | 0.0000000 | | | | | 0.0000000 | | | |
| IL1B+ Mp | | AC020916.1 | | | | 1.0759856 | | | 0.3350000 | | | | 0.0670000 | | | 0.0000000 | | | | | 0.0000000 | | | |
| NUPR1+ Mp | | CRIP1 | | | | 1.3444051 | | | 0.8110000 | | | | 0.4380000 | | | 0.0000000 | | | | | 0.0000000 | | | |
| NUPR1+ Mp | | CFD | | | | 1.3191655 | | | 0.9330000 | | | | 0.5680000 | | | 0.0000000 | | | | | 0.0000000 | | | |
| NUPR1+ Mp | | S100A4 | | | | 1.2793277 | | | 0.9840000 | | | | 0.8520000 | | | 0.0000000 | | | | | 0.0000000 | | | |
| NUPR1+ Mp | | NUPR1 | | | | 1.2457251 | | | 0.8450000 | | | | 0.3450000 | | | 0.0000000 | | | | | 0.0000000 | | | |
| NUPR1+ Mp | | S100A6 | | | | 1.2365644 | | | 0.9900000 | | | | 0.9860000 | | | 0.0000000 | | | | | 0.0000000 | | | |
| NUPR1+ Mp | | FTL | | | | 1.2171963 | | | 0.9920000 | | | | 0.9910000 | | | 0.0000000 | | | | | 0.0000000 | | | |
| NUPR1+ Mp | | MARCO | | | | 1.0640758 | | | 0.8950000 | | | | 0.4880000 | | | 0.0000000 | | | | | 0.0000000 | | | |
| NUPR1+ Mp | | C1QB | | | | 1.0413372 | | | 0.9610000 | | | | 0.5000000 | | | 0.0000000 | | | | | 0.0000000 | | | |
| NUPR1+ Mp | | RNASE1 | | | | 1.0393476 | | | 0.9830000 | | | | 0.7030000 | | | 0.0000000 | | | | | 0.0000000 | | | |
| NUPR1+ Mp | | MT-ND2 | | | | 0.9900219 | | | 0.9480000 | | | | 0.9260000 | | | 0.0000000 | | | | | 0.0000000 | | | |
| NUPR1+ Mp | | EMP3 | | | | 0.9729604 | | | 0.9640000 | | | | 0.7340000 | | | 0.0000000 | | | | | 0.0000000 | | | |
| NUPR1+ Mp | | C1QA | | | | 0.9637588 | | | 0.9760000 | | | | 0.5300000 | | | 0.0000000 | | | | | 0.0000000 | | | |
| NUPR1+ Mp | | GPNMB | | | | 0.8750704 | | | 0.8480000 | | | | 0.4280000 | | | 0.0000000 | | | | | 0.0000000 | | | |
| NUPR1+ Mp | | LGMN | | | | 0.8385073 | | | 0.8130000 | | | | 0.4090000 | | | 0.0000000 | | | | | 0.0000000 | | | |
| NUPR1+ Mp | | NCF1 | | | | 0.8066189 | | | 0.6380000 | | | | 0.2650000 | | | 0.0000000 | | | | | 0.0000000 | | | |
| NUPR1+ Mp | | MT-ND1 | | | | 0.7983931 | | | 0.9480000 | | | | 0.9290000 | | | 0.0000000 | | | | | 0.0000000 | | | |
| NUPR1+ Mp | | TIMD4 | | | | 0.7875099 | | | 0.4840000 | | | | 0.0870000 | | | 0.0000000 | | | | | 0.0000000 | | | |
| NUPR1+ Mp | | CTSZ | | | | 0.7278641 | | | 0.9300000 | | | | 0.6370000 | | | 0.0000000 | | | | | 0.0000000 | | | |
| NUPR1+ Mp | | LILRB5 | | | | 0.7276136 | | | 0.6220000 | | | | 0.1550000 | | | 0.0000000 | | | | | 0.0000000 | | | |
| NUPR1+ Mp | | FOLR2 | | | | 0.7183234 | | | 0.7170000 | | | | 0.3010000 | | | 0.0000000 | | | | | 0.0000000 | | | |
| SPP1+ Mo-Mp | | SPP1 | | | | 2.5400868 | | | 0.8300000 | | | | 0.1980000 | | | 0.0000000 | | | | | 0.0000000 | | | |
| SPP1+ Mo-Mp | | MT2A | | | | 1.8606991 | | | 0.7450000 | | | | 0.4910000 | | | 0.0000000 | | | | | 0.0000000 | | | |
| SPP1+ Mo-Mp | | GAPDH | | | | 1.8337090 | | | 0.9950000 | | | | 0.9350000 | | | 0.0000000 | | | | | 0.0000000 | | | |
| SPP1+ Mo-Mp | | MIF | | | | 1.7602135 | | | 0.8670000 | | | | 0.4390000 | | | 0.0000000 | | | | | 0.0000000 | | | |
| SPP1+ Mo-Mp | | MT1X | | | | 1.6499720 | | | 0.4560000 | | | | 0.2620000 | | | 0.0000000 | | | | | 0.0000000 | | | |
| SPP1+ Mo-Mp | | CSTB | | | | 1.6210797 | | | 0.9720000 | | | | 0.7930000 | | | 0.0000000 | | | | | 0.0000000 | | | |
| SPP1+ Mo-Mp | | C15orf48 | | | | 1.5097234 | | | 0.7370000 | | | | 0.2120000 | | | 0.0000000 | | | | | 0.0000000 | | | |
| SPP1+ Mo-Mp | | PLIN2 | | | | 1.4971725 | | | 0.7280000 | | | | 0.2970000 | | | 0.0000000 | | | | | 0.0000000 | | | |
| SPP1+ Mo-Mp | | TIMP1 | | | | 1.3712547 | | | 0.9080000 | | | | 0.7460000 | | | 0.0000000 | | | | | 0.0000000 | | | |
| SPP1+ Mo-Mp | | FBP1 | | | | 1.3591730 | | | 0.7300000 | | | | 0.2390000 | | | 0.0000000 | | | | | 0.0000000 | | | |
| SPP1+ Mo-Mp | | LGALS1 | | | | 1.2258491 | | | 0.9900000 | | | | 0.8510000 | | | 0.0000000 | | | | | 0.0000000 | | | |
| SPP1+ Mo-Mp | | ALDOA | | | | 1.2166515 | | | 0.8810000 | | | | 0.6300000 | | | 0.0000000 | | | | | 0.0000000 | | | |
| SPP1+ Mo-Mp | | VIM | | | | 1.1977967 | | | 1.0000000 | | | | 0.9870000 | | | 0.0000000 | | | | | 0.0000000 | | | |
| SPP1+ Mo-Mp | | ENO1 | | | | 1.1533263 | | | 0.9520000 | | | | 0.6630000 | | | 0.0000000 | | | | | 0.0000000 | | | |
| SPP1+ Mo-Mp | | TPI1 | | | | 1.1518639 | | | 0.9200000 | | | | 0.6220000 | | | 0.0000000 | | | | | 0.0000000 | | | |
| SPP1+ Mo-Mp | | LDHA | | | | 1.1137913 | | | 0.9030000 | | | | 0.5990000 | | | 0.0000000 | | | | | 0.0000000 | | | |
| SPP1+ Mo-Mp | | VCAN | | | | 1.0655310 | | | 0.6730000 | | | | 0.2720000 | | | 0.0000000 | | | | | 0.0000000 | | | |
| SPP1+ Mo-Mp | | S100A10 | | | | 1.0364066 | | | 0.9980000 | | | | 0.9570000 | | | 0.0000000 | | | | | 0.0000000 | | | |
| SPP1+ Mo-Mp | | SLC2A3 | | | | 0.9936634 | | | 0.5810000 | | | | 0.3200000 | | | 0.0000000 | | | | | 0.0000000 | | | |
| SPP1+ Mo-Mp | | PGK1 | | | | 0.9935661 | | | 0.8330000 | | | | 0.5150000 | | | 0.0000000 | | | | | 0.0000000 | | | |

**EC:** endothelial cell

**cluster:** annotation corresponding to cluster

**gene:** gene symbol

**avg_logFC:** average log2 fold change. Positive values indicate that the gene is more highly expressed in the cluster.

**pct.1:** The percentage of cells where the gene is detected in the cluster

**pct.2:** The percentage of cells where the gene is detected on average in the other clusters

**p_val:** p-value not adjusted for multiple test correction

**p_val_adj:** Adjusted p-value, based on bonferroni correction using all genes in the dataset, used to determine significance
