# Supplementary material for: Deconvolution of synovial myeloid cell subsets across pathotypes and role of COL3A1+ macrophages in rheumatoid arthritis remission
Source: Front Immunol. 2024 Mar 26;15:1307748. doi: 10.3389/fimmu.2024.1307748 (PMC11005452; doi:10.3389/fimmu.2024.1307748)
Supplement: Supplementary file 10 [file Table_4.docx]

**Supplementary Table 4.** Top 20 markers of each cluster in Liger integration

| cluster | gene | | | | avg_log2FC | | | | | pct.1 | | | | pct.2 | | | | p_val | | | | p_val_adj | | |
| --- | --- | --- | --- | --- | --- | --- | --- | --- | --- | --- | --- | --- | --- | --- | --- | --- | --- | --- | --- | --- | --- | --- | --- | --- |
| EC | IGFBP7 | | | | 2.0596120 | | | | | 0.9880000 | | | | 0.1460000 | | | | 0.0000000 | | | | 0.0000000 | | |
| EC | SPARCL1 | | | | 1.8311209 | | | | | 0.9810000 | | | | 0.1400000 | | | | 0.0000000 | | | | 0.0000000 | | |
| EC | TM4SF1 | | | | 1.7038775 | | | | | 0.9440000 | | | | 0.0380000 | | | | 0.0000000 | | | | 0.0000000 | | |
| EC | DARC | | | | 1.6816340 | | | | | 0.7400000 | | | | 0.0050000 | | | | 0.0000000 | | | | 0.0000000 | | |
| EC | GNG11 | | | | 1.6791786 | | | | | 0.9760000 | | | | 0.0920000 | | | | 0.0000000 | | | | 0.0000000 | | |
| EC | PLVAP | | | | 1.6504080 | | | | | 0.9100000 | | | | 0.0120000 | | | | 0.0000000 | | | | 0.0000000 | | |
| EC | AQP1 | | | | 1.6198096 | | | | | 0.9180000 | | | | 0.0350000 | | | | 0.0000000 | | | | 0.0000000 | | |
| EC | RAMP2 | | | | 1.6010380 | | | | | 0.9320000 | | | | 0.0260000 | | | | 0.0000000 | | | | 0.0000000 | | |
| EC | ECSCR | | | | 1.5429225 | | | | | 0.9170000 | | | | 0.0070000 | | | | 0.0000000 | | | | 0.0000000 | | |
| EC | CAV1 | | | | 1.5060031 | | | | | 0.9380000 | | | | 0.1210000 | | | | 0.0000000 | | | | 0.0000000 | | |
| EC | NPDC1 | | | | 1.5040389 | | | | | 0.9260000 | | | | 0.0340000 | | | | 0.0000000 | | | | 0.0000000 | | |
| EC | ADIRF | | | | 1.4774318 | | | | | 0.8240000 | | | | 0.1200000 | | | | 0.0000000 | | | | 0.0000000 | | |
| EC | VWF | | | | 1.4335862 | | | | | 0.8530000 | | | | 0.0160000 | | | | 0.0000000 | | | | 0.0000000 | | |
| EC | CLEC14A | | | | 1.3896415 | | | | | 0.8560000 | | | | 0.0080000 | | | | 0.0000000 | | | | 0.0000000 | | |
| EC | CRIP2 | | | | 1.3645794 | | | | | 0.9280000 | | | | 0.1500000 | | | | 0.0000000 | | | | 0.0000000 | | |
| EC | PTRF | | | | 1.3604764 | | | | | 0.8820000 | | | | 0.0330000 | | | | 0.0000000 | | | | 0.0000000 | | |
| EC | EGFL7 | | | | 1.3299925 | | | | | 0.8420000 | | | | 0.0530000 | | | | 0.0000000 | | | | 0.0000000 | | |
| EC | ID1 | | | | 1.3107488 | | | | | 0.7280000 | | | | 0.0380000 | | | | 0.0000000 | | | | 0.0000000 | | |
| EC | RAMP3 | | | | 1.3013091 | | | | | 0.7680000 | | | | 0.0130000 | | | | 0.0000000 | | | | 0.0000000 | | |
| EC | HSPG2 | | | | 1.2753944 | | | | | 0.8230000 | | | | 0.0330000 | | | | 0.0000000 | | | | 0.0000000 | | |
| CCL3+C1QA+ Mp | | | APOE | | | | 1.8106538 | | | | 0.8440000 | | | | | | 0.3940000 | | | 0.0000000 | | | 0.0000000 | |
| CCL3+C1QA+ Mp | | | | C1QB | | | | 1.2990137 | | | | 0.9660000 | | | 0.4770000 | | | | 0.0000000 | | | | | 0.0000000 |
| CCL3+C1QA+ Mp | | | | C1QA | | | | 1.2798183 | | | | 0.9790000 | | | 0.5080000 | | | | 0.0000000 | | | | | 0.0000000 |
| CCL3+C1QA+ Mp | | | | RGS1 | | | | 1.1476362 | | | | 0.6910000 | | | 0.3120000 | | | | 0.0000000 | | | | | 0.0000000 |
| CCL3+C1QA+ Mp | | | | IFI27 | | | | 1.0927498 | | | | 0.6550000 | | | 0.4940000 | | | | 0.0000000 | | | | | 0.0000000 |
| CCL3+C1QA+ Mp | | | | APOC1 | | | | 1.0890180 | | | | 0.7240000 | | | 0.2860000 | | | | 0.0000000 | | | | | 0.0000000 |
| CCL3+C1QA+ Mp | | | | CCL3 | | | | 1.0309646 | | | | 0.6480000 | | | 0.2980000 | | | | 0.0000000 | | | | | 0.0000000 |
| CCL3+C1QA+ Mp | | | | C1QC | | | | 1.0302382 | | | | 0.9370000 | | | 0.4510000 | | | | 0.0000000 | | | | | 0.0000000 |
| CCL3+C1QA+ Mp | | | | FTL | | | | 0.9674570 | | | | 0.9990000 | | | 0.9900000 | | | | 0.0000000 | | | | | 0.0000000 |
| CCL3+C1QA+ Mp | | | | FOS | | | | 0.9166165 | | | | 0.8740000 | | | 0.7300000 | | | | 0.0000000 | | | | | 0.0000000 |
| CCL3+C1QA+ Mp | | | | TMSB4X | | | | 0.8989227 | | | | 0.9990000 | | | 0.9930000 | | | | 0.0000000 | | | | | 0.0000000 |
| CCL3+C1QA+ Mp | | | | DUSP1 | | | | 0.8696826 | | | | 0.8330000 | | | 0.6990000 | | | | 0.0000000 | | | | | 0.0000000 |
| CCL3+C1QA+ Mp | | | | HLA-DRA | | | | 0.8242727 | | | | 0.9970000 | | | 0.9180000 | | | | 0.0000000 | | | | | 0.0000000 |
| CCL3+C1QA+ Mp | | | | CTSL | | | | 0.7702167 | | | | 0.8250000 | | | 0.5380000 | | | | 0.0000000 | | | | | 0.0000000 |
| CCL3+C1QA+ Mp | | | | RNASE1 | | | | 0.7634276 | | | | 0.9760000 | | | 0.6880000 | | | | 0.0000000 | | | | | 0.0000000 |
| CCL3+C1QA+ Mp | | | | MARCO | | | | 0.7587981 | | | | 0.8370000 | | | 0.4790000 | | | | 0.0000000 | | | | | 0.0000000 |
| CCL3+C1QA+ Mp | | | | HLA-DQA1 | | | | 0.7339793 | | | | 0.9290000 | | | 0.5580000 | | | | 0.0000000 | | | | | 0.0000000 |
| CCL3+C1QA+ Mp | | | | GLUL | | | | 0.7324557 | | | | 0.9060000 | | | 0.6460000 | | | | 0.0000000 | | | | | 0.0000000 |
| CCL3+C1QA+ Mp | | | | PLTP | | | | 0.7286301 | | | | 0.7720000 | | | 0.3750000 | | | | 0.0000000 | | | | | 0.0000000 |
| CCL3+C1QA+ Mp | | | | MT-CO3 | | | | 0.7264881 | | | | 0.9810000 | | | 0.9410000 | | | | 0.0000000 | | | | | 0.0000000 |
| CCL3+C1QA+ Mp | | | | APOE | | | | 1.8106538 | | | | 0.8440000 | | | 0.3940000 | | | | 0.0000000 | | | | | 0.0000000 |
| CD52+ Mo-Mp | | | | S100A9 | | | | 3.7076216 | | | | 0.9460000 | | | 0.4790000 | | | | 0.0000000 | | | | | 0.0000000 |
| CD52+ Mo-Mp | | | | S100A8 | | | | 3.3196352 | | | | 0.9010000 | | | 0.3840000 | | | | 0.0000000 | | | | | 0.0000000 |
| CD52+ Mo-Mp | | | | S100A12 | | | | 2.3679327 | | | | 0.6540000 | | | 0.0230000 | | | | 0.0000000 | | | | | 0.0000000 |
| CD52+ Mo-Mp | | | | FCN1 | | | | 2.2054445 | | | | 0.9450000 | | | 0.1560000 | | | | 0.0000000 | | | | | 0.0000000 |
| CD52+ Mo-Mp | | | | LYZ | | | | 1.9701413 | | | | 0.9380000 | | | 0.6880000 | | | | 0.0000000 | | | | | 0.0000000 |
| CD52+ Mo-Mp | | | | CD52 | | | | 1.7329584 | | | | 0.8060000 | | | 0.2060000 | | | | 0.0000000 | | | | | 0.0000000 |
| CD52+ Mo-Mp | | | | LST1 | | | | 1.5246650 | | | | 0.9380000 | | | 0.4890000 | | | | 0.0000000 | | | | | 0.0000000 |
| CD52+ Mo-Mp | | | | SRGN | | | | 1.4174061 | | | | 0.9970000 | | | 0.7990000 | | | | 0.0000000 | | | | | 0.0000000 |
| CD52+ Mo-Mp | | | | TIMP1 | | | | 1.3741945 | | | | 0.9340000 | | | 0.7530000 | | | | 0.0000000 | | | | | 0.0000000 |
| CD52+ Mo-Mp | | | | H3F3A | | | | 1.2624912 | | | | 0.9800000 | | | 0.8120000 | | | | 0.0000000 | | | | | 0.0000000 |
| CD52+ Mo-Mp | | | | CSTA | | | | 1.2555491 | | | | 0.7890000 | | | 0.2270000 | | | | 0.0000000 | | | | | 0.0000000 |
| CD52+ Mo-Mp | | | | CTSS | | | | 1.1636674 | | | | 0.9760000 | | | 0.7230000 | | | | 0.0000000 | | | | | 0.0000000 |
| CD52+ Mo-Mp | | | | RPL39 | | | | 1.1364750 | | | | 0.9980000 | | | 0.9530000 | | | | 0.0000000 | | | | | 0.0000000 |
| CD52+ Mo-Mp | | | | RPL34 | | | | 1.0872502 | | | | 0.9990000 | | | 0.9770000 | | | | 0.0000000 | | | | | 0.0000000 |
| CD52+ Mo-Mp | | | | H3F3B | | | | 1.0716900 | | | | 0.9970000 | | | 0.9540000 | | | | 0.0000000 | | | | | 0.0000000 |
| CD52+ Mo-Mp | | | | RPL21 | | | | 1.0616962 | | | | 0.9900000 | | | 0.9000000 | | | | 0.0000000 | | | | | 0.0000000 |
| CD52+ Mo-Mp | | | | NAMPT | | | | 1.0601719 | | | | 0.8850000 | | | 0.4990000 | | | | 0.0000000 | | | | | 0.0000000 |
| CD52+ Mo-Mp | | | | MNDA | | | | 1.0483028 | | | | 0.7160000 | | | 0.2820000 | | | | 0.0000000 | | | | | 0.0000000 |
| CD52+ Mo-Mp | | | | CORO1A | | | | 1.0385179 | | | | 0.7690000 | | | 0.2330000 | | | | 0.0000000 | | | | | 0.0000000 |
| CD52+ Mo-Mp | | | | EIF1 | | | | 0.9709877 | | | | 1.0000000 | | | 0.9730000 | | | | 0.0000000 | | | | | 0.0000000 |
| Censored | | | | IL8 | | | | 1.2936382 | | | | 0.5520000 | | | 0.0150000 | | | | 0.0000000 | | | | | 0.0000000 |
| Censored | | | | TMEM66 | | | | 1.1347204 | | | | 0.8450000 | | | 0.1210000 | | | | 0.0000000 | | | | | 0.0000000 |
| Censored | | | | ERO1L | | | | 0.7273350 | | | | 0.3970000 | | | 0.0270000 | | | | 0.0000000 | | | | | 0.0000000 |
| Censored | | | | CD97 | | | | 0.7035921 | | | | 0.4660000 | | | 0.0210000 | | | | 0.0000000 | | | | | 0.0000000 |
| Censored | | | | RPS4Y1 | | | | 0.9076193 | | | | 0.7070000 | | | 0.1000000 | | | | 0.0000000 | | | | | 0.0000000 |
| Censored | | | | RP11-290F20.3 | | | | 0.3960945 | | | | 0.2760000 | | | 0.0070000 | | | | 0.0000000 | | | | | 0.0000000 |
| Censored | | | | RAP1B | | | | 0.7523010 | | | | 0.7930000 | | | 0.3560000 | | | | 0.0000000 | | | | | 0.0000000 |
| Censored | | | | C19orf10 | | | | 0.6891166 | | | | 0.6030000 | | | 0.1090000 | | | | 0.0000000 | | | | | 0.0000000 |
| Censored | | | | LILRA3 | | | | 0.3487834 | | | | 0.1720000 | | | 0.0010000 | | | | 0.0000000 | | | | | 0.0000000 |
| Censored | | | | EIF4A1 | | | | 0.7112832 | | | | 0.8970000 | | | 0.4420000 | | | | 0.0000000 | | | | | 0.0000000 |
| Censored | | | | PRR13 | | | | 0.6261381 | | | | 0.7410000 | | | 0.3840000 | | | | 0.0000000 | | | | | 0.0000000 |
| Censored | | | | CD38 | | | | 0.4371730 | | | | 0.2760000 | | | 0.0410000 | | | | 0.0000000 | | | | | 0.0000000 |
| Censored | | | | FAM26F | | | | 0.6763589 | | | | 0.5690000 | | | 0.1250000 | | | | 0.0000000 | | | | | 0.0000000 |
| Censored | | | | AP1S2 | | | | 0.6900603 | | | | 0.8280000 | | | 0.4360000 | | | | 0.0000000 | | | | | 0.0000000 |
| Censored | | | | TNFSF13B | | | | 0.7264404 | | | | 0.7240000 | | | 0.2730000 | | | | 0.0000000 | | | | | 0.0000000 |
| Censored | | | | SLC39A8 | | | | 0.6611716 | | | | 0.4830000 | | | 0.1090000 | | | | 0.0000000 | | | | | 0.0000000 |
| Censored | | | | WASH4P | | | | 0.4142593 | | | | 0.3450000 | | | 0.0400000 | | | | 0.0000000 | | | | | 0.0000000 |
| Censored | | | | MRP63 | | | | 0.5065185 | | | | 0.4480000 | | | 0.0840000 | | | | 0.0000000 | | | | | 0.0000000 |
| Censored | | | | FYB | | | | 0.6629475 | | | | 0.7070000 | | | 0.2600000 | | | | 0.0000000 | | | | | 0.0000000 |
| Censored | | | | TMUB1 | | | | 0.2808804 | | | | 0.2240000 | | | 0.1010000 | | | | 0.0000000 | | | | | 0.0000001 |
| CLEC10A+ Mo | | HLA-DPB1 | | | | 1.7665141 | | | 0.9920000 | | | | 0.8310000 | | | 0.0000000 | | | | | 0.0000000 | | | |
| CLEC10A+ Mo | | FCER1A | | | | 1.6520648 | | | 0.3390000 | | | | 0.0110000 | | | 0.0000000 | | | | | 0.0000000 | | | |
| CLEC10A+ Mo | | HLA-DPA1 | | | | 1.5722909 | | | 0.9850000 | | | | 0.8250000 | | | 0.0000000 | | | | | 0.0000000 | | | |
| CLEC10A+ Mo | | HLA-DRA | | | | 1.5005447 | | | 0.9960000 | | | | 0.9200000 | | | 0.0000000 | | | | | 0.0000000 | | | |
| CLEC10A+ Mo | | HLA-DQB1 | | | | 1.4366176 | | | 0.9430000 | | | | 0.6680000 | | | 0.0000000 | | | | | 0.0000000 | | | |
| CLEC10A+ Mo | | HLA-DQA1 | | | | 1.4176351 | | | 0.9170000 | | | | 0.5680000 | | | 0.0000000 | | | | | 0.0000000 | | | |
| CLEC10A+ Mo | | HLA-DRB1 | | | | 1.4080803 | | | 0.9950000 | | | | 0.9130000 | | | 0.0000000 | | | | | 0.0000000 | | | |
| CLEC10A+ Mo | | GPR183 | | | | 1.3545909 | | | 0.7570000 | | | | 0.3170000 | | | 0.0000000 | | | | | 0.0000000 | | | |
| CLEC10A+ Mo | | CLEC10A | | | | 1.2746235 | | | 0.6000000 | | | | 0.0900000 | | | 0.0000000 | | | | | 0.0000000 | | | |
| CLEC10A+ Mo | | RPS2 | | | | 1.2071813 | | | 0.9820000 | | | | 0.9230000 | | | 0.0000000 | | | | | 0.0000000 | | | |
| CLEC10A+ Mo | | CXCR4 | | | | 1.0620079 | | | 0.6680000 | | | | 0.3020000 | | | 0.0000000 | | | | | 0.0000000 | | | |
| CLEC10A+ Mo | | RGS2 | | | | 1.0366542 | | | 0.7200000 | | | | 0.3670000 | | | 0.0000000 | | | | | 0.0000000 | | | |
| CLEC10A+ Mo | | LYZ | | | | 1.0235165 | | | 0.9440000 | | | | 0.6620000 | | | 0.0000000 | | | | | 0.0000000 | | | |
| CLEC10A+ Mo | | CREM | | | | 1.0204689 | | | 0.6450000 | | | | 0.2890000 | | | 0.0000000 | | | | | 0.0000000 | | | |
| CLEC10A+ Mo | | RPS17 | | | | 0.9976538 | | | 0.9260000 | | | | 0.5400000 | | | 0.0000000 | | | | | 0.0000000 | | | |
| CLEC10A+ Mo | | SRGN | | | | 0.9823667 | | | 0.9840000 | | | | 0.7810000 | | | 0.0000000 | | | | | 0.0000000 | | | |
| CLEC10A+ Mo | | RPL21 | | | | 0.9620061 | | | 0.9720000 | | | | 0.8940000 | | | 0.0000000 | | | | | 0.0000000 | | | |
| CLEC10A+ Mo | | RPL13A | | | | 0.9582800 | | | 0.9820000 | | | | 0.9000000 | | | 0.0000000 | | | | | 0.0000000 | | | |
| CLEC10A+ Mo | | CD74 | | | | 0.9478596 | | | 0.9980000 | | | | 0.9730000 | | | 0.0000000 | | | | | 0.0000000 | | | |
| CLEC10A+ Mo | | RPL10 | | | | 0.9310958 | | | 0.9860000 | | | | 0.9720000 | | | 0.0000000 | | | | | 0.0000000 | | | |
| COL3A1+ Mp | | PRG4 | | | | 3.6228436 | | | 0.8540000 | | | | 0.2450000 | | | 0.0000000 | | | | | 0.0000000 | | | |
| COL3A1+ Mp | | MT-RNR2 | | | | 2.3819930 | | | 0.7360000 | | | | 0.2170000 | | | 0.0000000 | | | | | 0.0000000 | | | |
| COL3A1+ Mp | | MT-RNR1 | | | | 2.3454452 | | | 0.7360000 | | | | 0.2070000 | | | 0.0000000 | | | | | 0.0000000 | | | |
| COL3A1+ Mp | | DCN | | | | 2.1068051 | | | 0.8830000 | | | | 0.1830000 | | | 0.0000000 | | | | | 0.0000000 | | | |
| COL3A1+ Mp | | PLA2G2A | | | | 2.0424871 | | | 0.8590000 | | | | 0.2070000 | | | 0.0000000 | | | | | 0.0000000 | | | |
| COL3A1+ Mp | | CLU | | | | 1.9822120 | | | 0.8450000 | | | | 0.2650000 | | | 0.0000000 | | | | | 0.0000000 | | | |
| COL3A1+ Mp | | COL3A1 | | | | 1.8993078 | | | 0.8880000 | | | | 0.1570000 | | | 0.0000000 | | | | | 0.0000000 | | | |
| COL3A1+ Mp | | COL1A2 | | | | 1.8765552 | | | 0.9260000 | | | | 0.1530000 | | | 0.0000000 | | | | | 0.0000000 | | | |
| COL3A1+ Mp | | FN1 | | | | 1.7447335 | | | 0.9760000 | | | | 0.6460000 | | | 0.0000000 | | | | | 0.0000000 | | | |
| COL3A1+ Mp | | LUM | | | | 1.7378403 | | | 0.9050000 | | | | 0.1680000 | | | 0.0000000 | | | | | 0.0000000 | | | |
| COL3A1+ Mp | | COL1A1 | | | | 1.6795312 | | | 0.8310000 | | | | 0.0850000 | | | 0.0000000 | | | | | 0.0000000 | | | |
| COL3A1+ Mp | | MGP | | | | 1.5967131 | | | 0.8960000 | | | | 0.2900000 | | | 0.0000000 | | | | | 0.0000000 | | | |
| COL3A1+ Mp | | C1S | | | | 1.3141884 | | | 0.8710000 | | | | 0.0910000 | | | 0.0000000 | | | | | 0.0000000 | | | |
| COL3A1+ Mp | | COL6A2 | | | | 1.2936088 | | | 0.8860000 | | | | 0.1180000 | | | 0.0000000 | | | | | 0.0000000 | | | |
| COL3A1+ Mp | | CRTAC1 | | | | 1.2929407 | | | 0.7410000 | | | | 0.1170000 | | | 0.0000000 | | | | | 0.0000000 | | | |
| COL3A1+ Mp | | PCOLCE | | | | 1.2830581 | | | 0.8520000 | | | | 0.0850000 | | | 0.0000000 | | | | | 0.0000000 | | | |
| COL3A1+ Mp | | SELENOP | | | | 1.2748786 | | | 0.4480000 | | | | 0.0630000 | | | 0.0000000 | | | | | 0.0000000 | | | |
| COL3A1+ Mp | | HTRA1 | | | | 1.2720474 | | | 0.8580000 | | | | 0.2480000 | | | 0.0000000 | | | | | 0.0000000 | | | |
| COL3A1+ Mp | | COL6A3 | | | | 1.2421089 | | | 0.7890000 | | | | 0.0620000 | | | 0.0000000 | | | | | 0.0000000 | | | |
| COL3A1+ Mp | | CCDC80 | | | | 1.2410396 | | | 0.8200000 | | | | 0.0710000 | | | 0.0000000 | | | | | 0.0000000 | | | |
| FOLR2+LYVE1+ Mo-Mp | | SEPP1 | | | | 2.2913889 | | | 0.8910000 | | | | 0.3290000 | | | 0.0000000 | | | | | 0.0000000 | | | |
| FOLR2+LYVE1+ Mo-Mp | | RNASE1 | | | | 1.7631149 | | | 0.9840000 | | | | 0.7120000 | | | 0.0000000 | | | | | 0.0000000 | | | |
| FOLR2+LYVE1+ Mo-Mp | | HMOX1 | | | | 1.5462357 | | | 0.7710000 | | | | 0.4010000 | | | 0.0000000 | | | | | 0.0000000 | | | |
| FOLR2+LYVE1+ Mo-Mp | | LYVE1 | | | | 1.4910208 | | | 0.6510000 | | | | 0.0780000 | | | 0.0000000 | | | | | 0.0000000 | | | |
| FOLR2+LYVE1+ Mo-Mp | | LGMN | | | | 1.4182644 | | | 0.8750000 | | | | 0.4180000 | | | 0.0000000 | | | | | 0.0000000 | | | |
| FOLR2+LYVE1+ Mo-Mp | | F13A1 | | | | 1.3593290 | | | 0.7320000 | | | | 0.1580000 | | | 0.0000000 | | | | | 0.0000000 | | | |
| FOLR2+LYVE1+ Mo-Mp | | FOLR2 | | | | 1.2790700 | | | 0.8190000 | | | | 0.3080000 | | | 0.0000000 | | | | | 0.0000000 | | | |
| FOLR2+LYVE1+ Mo-Mp | | C1QA | | | | 1.2268010 | | | 0.9930000 | | | | 0.5460000 | | | 0.0000000 | | | | | 0.0000000 | | | |
| FOLR2+LYVE1+ Mo-Mp | | EMP1 | | | | 1.0924366 | | | 0.7490000 | | | | 0.4380000 | | | 0.0000000 | | | | | 0.0000000 | | | |
| FOLR2+LYVE1+ Mo-Mp | | CST3 | | | | 1.0776347 | | | 0.9990000 | | | | 0.9770000 | | | 0.0000000 | | | | | 0.0000000 | | | |
| FOLR2+LYVE1+ Mo-Mp | | MT-ND2 | | | | 1.0765607 | | | 0.9910000 | | | | 0.9240000 | | | 0.0000000 | | | | | 0.0000000 | | | |
| FOLR2+LYVE1+ Mo-Mp | | C1QC | | | | 1.0642406 | | | 0.9450000 | | | | 0.4910000 | | | 0.0000000 | | | | | 0.0000000 | | | |
| FOLR2+LYVE1+ Mo-Mp | | MT-ND1 | | | | 1.0027279 | | | 0.9910000 | | | | 0.9270000 | | | 0.0000000 | | | | | 0.0000000 | | | |
| FOLR2+LYVE1+ Mo-Mp | | SLC40A1 | | | | 0.9203854 | | | 0.5700000 | | | | 0.1340000 | | | 0.0000000 | | | | | 0.0000000 | | | |
| FOLR2+LYVE1+ Mo-Mp | | STAB1 | | | | 0.8854270 | | | 0.6910000 | | | | 0.2700000 | | | 0.0000000 | | | | | 0.0000000 | | | |
| FOLR2+LYVE1+ Mo-Mp | | CALM2 | | | | 0.8064901 | | | 0.9390000 | | | | 0.8250000 | | | 0.0000000 | | | | | 0.0000000 | | | |
| FOLR2+LYVE1+ Mo-Mp | | FCGRT | | | | 0.7950058 | | | 0.8360000 | | | | 0.6830000 | | | 0.0000000 | | | | | 0.0000000 | | | |
| FOLR2+LYVE1+ Mo-Mp | | TPT1 | | | | 0.7949122 | | | 0.9980000 | | | | 0.9880000 | | | 0.0000000 | | | | | 0.0000000 | | | |
| FOLR2+LYVE1+ Mo-Mp | | BLVRB | | | | 0.7317202 | | | 0.6710000 | | | | 0.3590000 | | | 0.0000000 | | | | | 0.0000000 | | | |
| FOLR2+LYVE1+ Mo-Mp | | C1QB | | | | 0.7276860 | | | 0.9550000 | | | | 0.5190000 | | | 0.0000000 | | | | | 0.0000000 | | | |
| IL1B+ Mp | | MT-RNR1 | | | | 1.7684632 | | | 0.8600000 | | | | 0.1960000 | | | 0.0000000 | | | | | 0.0000000 | | | |
| IL1B+ Mp | | TNFAIP3 | | | | 1.7370078 | | | 0.7490000 | | | | 0.1750000 | | | 0.0000000 | | | | | 0.0000000 | | | |
| IL1B+ Mp | | MT-RNR2 | | | | 1.7172682 | | | 0.8740000 | | | | 0.2040000 | | | 0.0000000 | | | | | 0.0000000 | | | |
| IL1B+ Mp | | NFKBIA | | | | 1.4829806 | | | 0.8980000 | | | | 0.6050000 | | | 0.0000000 | | | | | 0.0000000 | | | |
| IL1B+ Mp | | CXCL2 | | | | 1.3924780 | | | 0.8200000 | | | | 0.3480000 | | | 0.0000000 | | | | | 0.0000000 | | | |
| IL1B+ Mp | | MMP19 | | | | 1.2361512 | | | 0.4860000 | | | | 0.1170000 | | | 0.0000000 | | | | | 0.0000000 | | | |
| IL1B+ Mp | | PNRC1 | | | | 1.0298397 | | | 0.9040000 | | | | 0.5540000 | | | 0.0000000 | | | | | 0.0000000 | | | |
| IL1B+ Mp | | NLRP3 | | | | 0.9795496 | | | 0.4310000 | | | | 0.0860000 | | | 0.0000000 | | | | | 0.0000000 | | | |
| IL1B+ Mp | | NFKBIZ | | | | 0.9675051 | | | 0.6440000 | | | | 0.2410000 | | | 0.0000000 | | | | | 0.0000000 | | | |
| IL1B+ Mp | | ZFAND5 | | | | 0.9210373 | | | 0.6980000 | | | | 0.3690000 | | | 0.0000000 | | | | | 0.0000000 | | | |
| IL1B+ Mp | | ATP5F1E | | | | 0.7837326 | | | 0.6190000 | | | | 0.1750000 | | | 0.0000000 | | | | | 0.0000000 | | | |
| IL1B+ Mp | | VEGFA | | | | 0.7919318 | | | 0.5220000 | | | | 0.1260000 | | | 5.22675444424901e-315 | | | | | 1.28102524674099e-310 | | | |
| IL1B+ Mp | | IL1B | | | | 1.6072415 | | | 0.5650000 | | | | 0.1690000 | | | 0.0000000 | | | | | 0.0000000 | | | |
| IL1B+ Mp | | FYB1 | | | | 0.6839578 | | | 0.4530000 | | | | 0.0840000 | | | 0.0000000 | | | | | 0.0000000 | | | |
| IL1B+ Mp | | RACK1 | | | | 0.8437018 | | | 0.5980000 | | | | 0.1680000 | | | 0.0000000 | | | | | 0.0000000 | | | |
| IL1B+ Mp | | ZNF331 | | | | 0.9080917 | | | 0.6200000 | | | | 0.2330000 | | | 0.0000000 | | | | | 0.0000000 | | | |
| IL1B+ Mp | | DMXL2 | | | | 0.6368097 | | | 0.5060000 | | | | 0.1410000 | | | 0.0000000 | | | | | 0.0000000 | | | |
| IL1B+ Mp | | FN1 | | | | 1.3720461 | | | 0.9200000 | | | | 0.6500000 | | | 0.0000000 | | | | | 0.0000000 | | | |
| IL1B+ Mp | | ADAM28 | | | | 0.4925226 | | | 0.4140000 | | | | 0.0810000 | | | 0.0000000 | | | | | 0.0000000 | | | |
| IL1B+ Mp | | ATP5MC2 | | | | 0.6245234 | | | 0.5230000 | | | | 0.1400000 | | | 0.0000000 | | | | | 0.0000000 | | | |
| NUPR1+ Mp | | CRIP1 | | | | 1.3704698 | | | 0.8180000 | | | | 0.4390000 | | | 0.0000000 | | | | | 0.0000000 | | | |
| NUPR1+ Mp | | S100A4 | | | | 1.3302870 | | | 0.9780000 | | | | 0.8540000 | | | 0.0000000 | | | | | 0.0000000 | | | |
| NUPR1+ Mp | | NUPR1 | | | | 1.3179500 | | | 0.8740000 | | | | 0.3460000 | | | 0.0000000 | | | | | 0.0000000 | | | |
| NUPR1+ Mp | | S100A6 | | | | 1.2738065 | | | 0.9800000 | | | | 0.9870000 | | | 0.0000000 | | | | | 0.0000000 | | | |
| NUPR1+ Mp | | FTL | | | | 1.2530304 | | | 0.9780000 | | | | 0.9930000 | | | 0.0000000 | | | | | 0.0000000 | | | |
| NUPR1+ Mp | | CFD | | | | 1.2453439 | | | 0.9300000 | | | | 0.5720000 | | | 0.0000000 | | | | | 0.0000000 | | | |
| NUPR1+ Mp | | MARCO | | | | 1.1669538 | | | 0.9350000 | | | | 0.4820000 | | | 0.0000000 | | | | | 0.0000000 | | | |
| NUPR1+ Mp | | C1QB | | | | 1.1290800 | | | 0.9730000 | | | | 0.4990000 | | | 0.0000000 | | | | | 0.0000000 | | | |
| NUPR1+ Mp | | RNASE1 | | | | 1.0367689 | | | 0.9660000 | | | | 0.7030000 | | | 0.0000000 | | | | | 0.0000000 | | | |
| NUPR1+ Mp | | EMP3 | | | | 1.0345978 | | | 0.9790000 | | | | 0.7320000 | | | 0.0000000 | | | | | 0.0000000 | | | |
| NUPR1+ Mp | | C1QA | | | | 1.0180571 | | | 0.9870000 | | | | 0.5290000 | | | 0.0000000 | | | | | 0.0000000 | | | |
| NUPR1+ Mp | | MT-ND2 | | | | 0.9768083 | | | 0.9350000 | | | | 0.9290000 | | | 0.0000000 | | | | | 0.0000000 | | | |
| NUPR1+ Mp | | GPNMB | | | | 0.9096925 | | | 0.8820000 | | | | 0.4260000 | | | 0.0000000 | | | | | 0.0000000 | | | |
| NUPR1+ Mp | | TMSB4X | | | | 0.8760611 | | | 0.9760000 | | | | 0.9960000 | | | 0.0000000 | | | | | 0.0000000 | | | |
| NUPR1+ Mp | | TIMD4 | | | | 0.8680462 | | | 0.5230000 | | | | 0.0820000 | | | 0.0000000 | | | | | 0.0000000 | | | |
| NUPR1+ Mp | | NCF1 | | | | 0.8611844 | | | 0.6570000 | | | | 0.2620000 | | | 0.0000000 | | | | | 0.0000000 | | | |
| NUPR1+ Mp | | LGMN | | | | 0.8048491 | | | 0.8120000 | | | | 0.4080000 | | | 0.0000000 | | | | | 0.0000000 | | | |
| NUPR1+ Mp | | S100A10 | | | | 0.7963540 | | | 0.9760000 | | | | 0.9600000 | | | 0.0000000 | | | | | 0.0000000 | | | |
| NUPR1+ Mp | | MT-ND1 | | | | 0.7618841 | | | 0.9330000 | | | | 0.9320000 | | | 0.0000000 | | | | | 0.0000000 | | | |
| NUPR1+ Mp | | C1QC | | | | 0.7413595 | | | 0.9310000 | | | | 0.4740000 | | | 0.0000000 | | | | | 0.0000000 | | | |
| SPP1+ Mo-Mp | | SPP1 | | | | 3.1365811 | | | 0.8780000 | | | | 0.1890000 | | | 0.0000000 | | | | | 0.0000000 | | | |
| SPP1+ Mo-Mp | | GAPDH | | | | 1.9123195 | | | 0.9950000 | | | | 0.9340000 | | | 0.0000000 | | | | | 0.0000000 | | | |
| SPP1+ Mo-Mp | | MT2A | | | | 1.8798351 | | | 0.7480000 | | | | 0.4930000 | | | 0.0000000 | | | | | 0.0000000 | | | |
| SPP1+ Mo-Mp | | CSTB | | | | 1.8102228 | | | 0.9820000 | | | | 0.7890000 | | | 0.0000000 | | | | | 0.0000000 | | | |
| SPP1+ Mo-Mp | | MIF | | | | 1.7577400 | | | 0.8780000 | | | | 0.4360000 | | | 0.0000000 | | | | | 0.0000000 | | | |
| SPP1+ Mo-Mp | | MT1X | | | | 1.6466508 | | | 0.4500000 | | | | 0.2650000 | | | 0.0000000 | | | | | 0.0000000 | | | |
| SPP1+ Mo-Mp | | FBP1 | | | | 1.5333374 | | | 0.7570000 | | | | 0.2330000 | | | 0.0000000 | | | | | 0.0000000 | | | |
| SPP1+ Mo-Mp | | PLIN2 | | | | 1.4815889 | | | 0.7300000 | | | | 0.2930000 | | | 0.0000000 | | | | | 0.0000000 | | | |
| SPP1+ Mo-Mp | | C15orf48 | | | | 1.4106666 | | | 0.7230000 | | | | 0.2120000 | | | 0.0000000 | | | | | 0.0000000 | | | |
| SPP1+ Mo-Mp | | VIM | | | | 1.3646995 | | | 0.9990000 | | | | 0.9870000 | | | 0.0000000 | | | | | 0.0000000 | | | |
| SPP1+ Mo-Mp | | TIMP1 | | | | 1.2722972 | | | 0.8610000 | | | | 0.7510000 | | | 0.0000000 | | | | | 0.0000000 | | | |
| SPP1+ Mo-Mp | | FN1 | | | | 1.2313442 | | | 0.8980000 | | | | 0.6450000 | | | 0.0000000 | | | | | 0.0000000 | | | |
| SPP1+ Mo-Mp | | LGALS1 | | | | 1.1708780 | | | 0.9910000 | | | | 0.8520000 | | | 0.0000000 | | | | | 0.0000000 | | | |
| SPP1+ Mo-Mp | | ALDOA | | | | 1.1566526 | | | 0.8510000 | | | | 0.6340000 | | | 0.0000000 | | | | | 0.0000000 | | | |
| SPP1+ Mo-Mp | | ENO1 | | | | 1.1413811 | | | 0.9540000 | | | | 0.6630000 | | | 0.0000000 | | | | | 0.0000000 | | | |
| SPP1+ Mo-Mp | | ATP5F1E | | | | 1.1290855 | | | 0.5600000 | | | | 0.1680000 | | | 0.0000000 | | | | | 0.0000000 | | | |
| SPP1+ Mo-Mp | | S100A10 | | | | 1.1157637 | | | 0.9960000 | | | | 0.9570000 | | | 0.0000000 | | | | | 0.0000000 | | | |
| SPP1+ Mo-Mp | | TPI1 | | | | 1.1067301 | | | 0.9060000 | | | | 0.6230000 | | | 0.0000000 | | | | | 0.0000000 | | | |
| SPP1+ Mo-Mp | | LDHA | | | | 1.0674245 | | | 0.8880000 | | | | 0.6020000 | | | 0.0000000 | | | | | 0.0000000 | | | |
| SPP1+ Mo-Mp | | PKM | | | | 1.0540682 | | | 0.9330000 | | | | 0.6400000 | | | 0.0000000 | | | | | 0.0000000 | | | |

**EC:** endothelial cell

**cluster:** annotation corresponding to cluster

**gene:** gene symbol

**avg_logFC:** average log2 fold change. Positive values indicate that the gene is more highly expressed in the cluster.

**pct.1:** The percentage of cells where the gene is detected in the cluster

**pct.2:** The percentage of cells where the gene is detected on average in the other clusters

**p_val:** p-value not adjusted for multiple test correction

**p_val_adj:** Adjusted p-value, based on bonferroni correction using all genes in the dataset, used to determine significance
