# Supplementary material for: Deconvolution of synovial myeloid cell subsets across pathotypes and role of COL3A1+ macrophages in rheumatoid arthritis remission
Source: Front Immunol. 2024 Mar 26;15:1307748. doi: 10.3389/fimmu.2024.1307748 (PMC11005452; doi:10.3389/fimmu.2024.1307748)
Supplement: Supplementary file 11 [file Table_5.docx]

**Supplementary Table 5.** KEGG pathway enrichment analysis of Mo/Mp subtypes

| Cluster | Description | p.adjust | q value | GeneRatio |
| --- | --- | --- | --- | --- |
| CCL3+C1QA+ Mp | Staphylococcus aureus infection | 7.72E-08 | 5.46E-08 | 11/71 |
| CCL3+C1QA+ Mp | Antigen processing and presentation | 8.28E-08 | 5.86E-08 | 10/71 |
| CCL3+C1QA+ Mp | Rheumatoid arthritis | 3.2E-07 | 2.27E-07 | 10/71 |
| CCL3+C1QA+ Mp | Lysosome | 7.11E-06 | 5.03E-06 | 10/71 |
| CCL3+C1QA+ Mp | Systemic lupus erythematosus | 7.54E-06 | 5.34E-06 | 10/71 |
| CCL3+C1QA+ Mp | Leishmaniasis | 7.88E-06 | 5.58E-06 | 8/71 |
| CCL3+C1QA+ Mp | Phagosome | 1.4E-05 | 9.91E-06 | 10/71 |
| CCL3+C1QA+ Mp | Complement and coagulation cascades | 1.4E-05 | 9.91E-06 | 8/71 |
| CCL3+C1QA+ Mp | Pertussis | 7E-05 | 4.96E-05 | 7/71 |
| CCL3+C1QA+ Mp | Epstein-Barr virus infection | 0.00014 | 9.94E-05 | 10/71 |
| CCL3+C1QA+ Mp | Chagas disease | 0.000405 | 0.000287 | 7/71 |
| CCL3+C1QA+ Mp | Toll-like receptor signaling pathway | 0.000421 | 0.000298 | 7/71 |
| CCL3+C1QA+ Mp | Toxoplasmosis | 0.000628 | 0.000445 | 7/71 |
| CCL3+C1QA+ Mp | Lipid and atherosclerosis | 0.001101 | 0.000779 | 9/71 |
| CCL3+C1QA+ Mp | Osteoclast differentiation | 0.001273 | 0.000901 | 7/71 |
| CCL3+C1QA+ Mp | Th1 and Th2 cell differentiation | 0.001382 | 0.000978 | 6/71 |
| CCL3+C1QA+ Mp | Asthma | 0.001382 | 0.000978 | 4/71 |
| CCL3+C1QA+ Mp | Tuberculosis | 0.001396 | 0.000988 | 8/71 |
| CCL3+C1QA+ Mp | Apoptosis | 0.001396 | 0.000988 | 7/71 |
| CCL3+C1QA+ Mp | Viral myocarditis | 0.001396 | 0.000988 | 5/71 |
| CD52+ Mo-Mp | Ribosome | 1.16E-75 | 1.06E-75 | 74/204 |
| CD52+ Mo-Mp | Coronavirus disease - COVID-19 | 1.37E-70 | 1.25E-70 | 79/204 |
| CLEC10A+ Mo | Ribosome | 1.85E-86 | 1.63E-86 | 76/171 |
| CLEC10A+ Mo | Coronavirus disease - COVID-19 | 1.24E-76 | 1.1E-76 | 78/171 |
| CLEC10A+ Mo | Antigen processing and presentation | 1.41E-11 | 1.25E-11 | 17/171 |
| CLEC10A+ Mo | Allograft rejection | 3.32E-10 | 2.94E-10 | 12/171 |
| CLEC10A+ Mo | Viral myocarditis | 4.43E-10 | 3.92E-10 | 14/171 |
| CLEC10A+ Mo | Graft-versus-host disease | 8.41E-10 | 7.44E-10 | 12/171 |
| CLEC10A+ Mo | Type I diabetes mellitus | 9.82E-10 | 8.68E-10 | 12/171 |
| CLEC10A+ Mo | Intestinal immune network for IgA production | 4.64E-09 | 4.1E-09 | 12/171 |
| CLEC10A+ Mo | Asthma | 6.92E-09 | 6.12E-09 | 10/171 |
| CLEC10A+ Mo | Autoimmune thyroid disease | 9.98E-09 | 8.83E-09 | 12/171 |
| CLEC10A+ Mo | Phagosome | 1.43E-06 | 1.27E-06 | 16/171 |
| CLEC10A+ Mo | Rheumatoid arthritis | 6.31E-06 | 5.58E-06 | 12/171 |
| CLEC10A+ Mo | Hematopoietic cell lineage | 1.16E-05 | 1.03E-05 | 12/171 |
| CLEC10A+ Mo | Leishmaniasis | 4.82E-05 | 4.26E-05 | 10/171 |
| CLEC10A+ Mo | Inflammatory bowel disease | 8.13E-05 | 7.19E-05 | 9/171 |
| CLEC10A+ Mo | Influenza A | 0.00013 | 0.000115 | 14/171 |
| CLEC10A+ Mo | Epstein-Barr virus infection | 0.00019 | 0.000168 | 15/171 |
| CLEC10A+ Mo | Toxoplasmosis | 0.00019 | 0.000168 | 11/171 |
| CLEC10A+ Mo | Tuberculosis | 0.000192 | 0.000169 | 14/171 |
| CLEC10A+ Mo | Cell adhesion molecules | 0.000192 | 0.000169 | 13/171 |
| COL3A1+ Mp | ECM-receptor interaction | 2.75E-09 | 2E-09 | 17/190 |
| COL3A1+ Mp | Focal adhesion | 3.4E-09 | 2.48E-09 | 24/190 |
| COL3A1+ Mp | Protein processing in endoplasmic reticulum | 2.54E-08 | 1.85E-08 | 21/190 |
| COL3A1+ Mp | Fluid shear stress and atherosclerosis | 1.1E-06 | 7.98E-07 | 17/190 |
| COL3A1+ Mp | PI3K-Akt signaling pathway | 7.07E-06 | 5.15E-06 | 26/190 |
| COL3A1+ Mp | Proteoglycans in cancer | 9.56E-06 | 6.96E-06 | 19/190 |
| COL3A1+ Mp | Antigen processing and presentation | 5.45E-05 | 3.97E-05 | 11/190 |
| COL3A1+ Mp | AGE-RAGE signaling pathway in diabetic complications | 9.16E-05 | 6.67E-05 | 12/190 |
| COL3A1+ Mp | Relaxin signaling pathway | 0.000224 | 0.000163 | 13/190 |
| COL3A1+ Mp | Prion disease | 0.000383 | 0.000279 | 19/190 |
| COL3A1+ Mp | Estrogen signaling pathway | 0.000383 | 0.000279 | 13/190 |
| COL3A1+ Mp | Protein digestion and absorption | 0.000493 | 0.000359 | 11/190 |
| COL3A1+ Mp | Lipid and atherosclerosis | 0.000683 | 0.000497 | 16/190 |
| COL3A1+ Mp | IL-17 signaling pathway | 0.000975 | 0.00071 | 10/190 |
| COL3A1+ Mp | Pertussis | 0.000975 | 0.00071 | 9/190 |
| COL3A1+ Mp | Human papillomavirus infection | 0.001178 | 0.000858 | 20/190 |
| COL3A1+ Mp | Amoebiasis | 0.001707 | 0.001243 | 10/190 |
| COL3A1+ Mp | Malaria | 0.001892 | 0.001378 | 7/190 |
| COL3A1+ Mp | Salmonella infection | 0.00266 | 0.001938 | 16/190 |
| COL3A1+ Mp | Oxidative phosphorylation | 0.003269 | 0.002381 | 11/190 |
| FOLR2+LYVE1+ Mo-Mp | Coronavirus disease - COVID-19 | 1.49E-12 | 1.33E-12 | 20/75 |
| FOLR2+LYVE1+ Mo-Mp | Ribosome | 2.15E-07 | 1.92E-07 | 13/75 |
| FOLR2+LYVE1+ Mo-Mp | Complement and coagulation cascades | 0.00064 | 0.000572 | 7/75 |
| FOLR2+LYVE1+ Mo-Mp | Pertussis | 0.024708 | 0.022107 | 5/75 |
| FOLR2+LYVE1+ Mo-Mp | Transcriptional misregulation in cancer | 0.048569 | 0.043456 | 7/75 |
| FOLR2+LYVE1+ Mo-Mp | Fluid shear stress and atherosclerosis | 0.048569 | 0.043456 | 6/75 |
| FOLR2+LYVE1+ Mo-Mp | Viral protein interaction with cytokine and cytokine receptor | 0.048569 | 0.043456 | 5/75 |
| IL1B+ Mp | TNF signaling pathway | 6.62E-08 | 4.51E-08 | 20/261 |
| IL1B+ Mp | Osteoclast differentiation | 6.62E-08 | 4.51E-08 | 21/261 |
| IL1B+ Mp | NF-kappa B signaling pathway | 3.34E-07 | 2.27E-07 | 18/261 |
| IL1B+ Mp | HIF-1 signaling pathway | 3.48E-06 | 2.37E-06 | 17/261 |
| IL1B+ Mp | Chemokine signaling pathway | 9.15E-06 | 6.23E-06 | 22/261 |
| IL1B+ Mp | Yersinia infection | 6.73E-05 | 4.59E-05 | 17/261 |
| IL1B+ Mp | FoxO signaling pathway | 0.000147 | 9.98E-05 | 16/261 |
| IL1B+ Mp | Leishmaniasis | 0.000173 | 0.000118 | 12/261 |
| IL1B+ Mp | NOD-like receptor signaling pathway | 0.000207 | 0.000141 | 19/261 |
| IL1B+ Mp | Human cytomegalovirus infection | 0.000251 | 0.000171 | 21/261 |
| IL1B+ Mp | Tuberculosis | 0.0004 | 0.000272 | 18/261 |
| IL1B+ Mp | Chagas disease | 0.000449 | 0.000306 | 13/261 |
| IL1B+ Mp | PD-L1 expression and PD-1 checkpoint pathway in cancer | 0.000475 | 0.000324 | 12/261 |
| IL1B+ Mp | C-type lectin receptor signaling pathway | 0.000475 | 0.000324 | 13/261 |
| IL1B+ Mp | Parathyroid hormone synthesis, secretion and action | 0.000545 | 0.000372 | 13/261 |
| IL1B+ Mp | IL-17 signaling pathway | 0.000666 | 0.000453 | 12/261 |
| IL1B+ Mp | Transcriptional misregulation in cancer | 0.000666 | 0.000453 | 18/261 |
| IL1B+ Mp | Kaposi sarcoma-associated herpesvirus infection | 0.000673 | 0.000459 | 18/261 |
| IL1B+ Mp | Lipid and atherosclerosis | 0.000737 | 0.000502 | 19/261 |
| IL1B+ Mp | Toxoplasmosis | 0.000737 | 0.000502 | 13/261 |
| NUPR1+ Mp | Lysosome | 1.51E-08 | 1.34E-08 | 13/73 |
| NUPR1+ Mp | Complement and coagulation cascades | 6.08E-05 | 5.39E-05 | 8/73 |
| NUPR1+ Mp | Staphylococcus aureus infection | 9.44E-05 | 8.37E-05 | 8/73 |
| NUPR1+ Mp | Phagosome | 0.000269 | 0.000238 | 9/73 |
| NUPR1+ Mp | Prion disease | 0.004129 | 0.00366 | 10/73 |
| NUPR1+ Mp | Diabetic cardiomyopathy | 0.009833 | 0.008717 | 8/73 |
| NUPR1+ Mp | Ferroptosis | 0.009833 | 0.008717 | 4/73 |
| NUPR1+ Mp | Pertussis | 0.010361 | 0.009185 | 5/73 |
| NUPR1+ Mp | Osteoclast differentiation | 0.015561 | 0.013794 | 6/73 |
| NUPR1+ Mp | Cholesterol metabolism | 0.015887 | 0.014082 | 4/73 |
| NUPR1+ Mp | Oxidative phosphorylation | 0.01616 | 0.014325 | 6/73 |
| NUPR1+ Mp | Chemical carcinogenesis - reactive oxygen species | 0.044541 | 0.039482 | 7/73 |
| SPP1+ Mo-Mp | HIF-1 signaling pathway | 8.83E-10 | 6.32E-10 | 18/164 |
| SPP1+ Mo-Mp | Phagosome | 1.68E-08 | 1.2E-08 | 19/164 |
| SPP1+ Mo-Mp | Glycolysis / Gluconeogenesis | 4.21E-08 | 3.02E-08 | 13/164 |
| SPP1+ Mo-Mp | Carbon metabolism | 3.63E-06 | 2.6E-06 | 14/164 |
| SPP1+ Mo-Mp | Fluid shear stress and atherosclerosis | 4.91E-06 | 3.52E-06 | 15/164 |
| SPP1+ Mo-Mp | Biosynthesis of amino acids | 9.92E-06 | 7.1E-06 | 11/164 |
| SPP1+ Mo-Mp | Vibrio cholerae infection | 1.83E-05 | 1.31E-05 | 9/164 |
| SPP1+ Mo-Mp | Protein processing in endoplasmic reticulum | 4.6E-05 | 3.29E-05 | 15/164 |
| SPP1+ Mo-Mp | Rheumatoid arthritis | 6.04E-05 | 4.32E-05 | 11/164 |
| SPP1+ Mo-Mp | Oxidative phosphorylation | 6.1E-05 | 4.37E-05 | 13/164 |
| SPP1+ Mo-Mp | Diabetic cardiomyopathy | 6.1E-05 | 4.37E-05 | 16/164 |
| SPP1+ Mo-Mp | Antigen processing and presentation | 6.36E-05 | 4.55E-05 | 10/164 |
| SPP1+ Mo-Mp | Salmonella infection | 0.000174 | 0.000125 | 17/164 |
| SPP1+ Mo-Mp | NOD-like receptor signaling pathway | 0.000347 | 0.000248 | 14/164 |
| SPP1+ Mo-Mp | Parkinson disease | 0.000347 | 0.000248 | 17/164 |
| SPP1+ Mo-Mp | Viral myocarditis | 0.000347 | 0.000248 | 8/164 |
| SPP1+ Mo-Mp | Pentose phosphate pathway | 0.000347 | 0.000248 | 6/164 |
| SPP1+ Mo-Mp | Natural killer cell mediated cytotoxicity | 0.000818 | 0.000585 | 11/164 |
| SPP1+ Mo-Mp | Central carbon metabolism in cancer | 0.000943 | 0.000675 | 8/164 |
| SPP1+ Mo-Mp | Prion disease | 0.001343 | 0.000961 | 16/164 |
