# Supplementary material for: Deconvolution of synovial myeloid cell subsets across pathotypes and role of COL3A1+ macrophages in rheumatoid arthritis remission
Source: Front Immunol. 2024 Mar 26;15:1307748. doi: 10.3389/fimmu.2024.1307748 (PMC11005452; doi:10.3389/fimmu.2024.1307748)
Supplement: Supplementary file 12 [file Table_6.docx]

**Supplementary Table 6.** Top 50 differential expressed genes over the pseudotime trajectory

| Gene | p value | q value |
| --- | --- | --- |
| CTGF | 0 | 0 |
| COL1A2 | 0 | 0 |
| RPL7 | 0 | 0 |
| PLA2G2A | 0 | 0 |
| DCN | 0 | 0 |
| LUM | 0 | 0 |
| EFEMP1 | 0 | 0 |
| PCOLCE | 0 | 0 |
| RPL23A | 0 | 0 |
| CCDC80 | 0 | 0 |
| C1S | 0 | 0 |
| CALD1 | 0 | 0 |
| PLAC9 | 0 | 0 |
| COL6A2 | 0 | 0 |
| BGN | 0 | 0 |
| FSTL1 | 0 | 0 |
| NNMT | 0 | 0 |
| C19orf10 | 0 | 0 |
| PPAP2A | 0 | 0 |
| MTRNR2L10 | 0 | 0 |
| MTRNR2L12 | 0 | 0 |
| MTRNR2L8 | 0 | 0 |
| 45184 | 0 | 0 |
| SEPW1 | 0 | 0 |
| NBEAL1 | 0 | 0 |
| COMP | 0 | 0 |
| LHFP | 0 | 0 |
| NGFRAP1 | 0 | 0 |
| ATP5J | 0 | 0 |
| SCRG1 | 0 | 0 |
| MDK | 0 | 0 |
| WDR83OS | 0 | 0 |
| GLTSCR2 | 0 | 0 |
| LOXL1 | 0 | 0 |
| VKORC1 | 0 | 0 |
| ATP5D | 0 | 0 |
| ANGPTL2 | 0 | 0 |
| EFEMP2 | 0 | 0 |
| THY1 | 0 | 0 |
| AK1 | 0 | 0 |
| PRKCDBP | 0 | 0 |
| ATP5E | 0 | 0 |
| RPL22 | 0 | 0 |
| S100A13 | 0 | 0 |
| RPS10 | 0 | 0 |
| TPPP3 | 0 | 0 |
| IGFBP4 | 0 | 0 |
| PTGDS | 0 | 0 |
| CXCL12 | 0 | 0 |
| ADIRF | 0 | 0 |
