# Supplementary material for: Deconvolution of synovial myeloid cell subsets across pathotypes and role of COL3A1+ macrophages in rheumatoid arthritis remission
Source: Front Immunol. 2024 Mar 26;15:1307748. doi: 10.3389/fimmu.2024.1307748 (PMC11005452; doi:10.3389/fimmu.2024.1307748)
Supplement: Supplementary file 13 [file Table_7.docx]

**Supplementary Table 7.** Pearson correlation result of Mo/Mp subtypes with clinical indicators across pathotypes

| Mo/Mp subtype | Pathotype | Clinical Indicators | Correlation (R) | p_value | FDR adjusted p_value |
| --- | --- | --- | --- | --- | --- |
| CCL3+C1QA+ Mp | All | ESR | 0.034429 | 0.760263 | 0.844736 |
|  |  | CRP | -0.07084 | 0.532363 | 0.823357 |
|  |  | CCP | 0.193811 | 0.082977 | 0.414885 |
|  |  | RF | 0.041396 | 0.713673 | 0.844736 |
|  |  | VAS | 0.110174 | 0.327511 | 0.797294 |
|  |  | TJC | -0.10778 | 0.338201 | 0.797294 |
|  |  | SJC | -0.009 | 0.93641 | 0.93641 |
|  |  | HAQ | 0.095655 | 0.398647 | 0.797294 |
|  |  | DAS28. | 0.062998 | 0.57635 | 0.823357 |
|  |  | IS | 0.370973 | 0.000896 | 0.008957 |
|  | Fibroid | ESR | 0.045989 | 0.865702 | 0.922843 |
|  |  | CRP | 0.103497 | 0.702881 | 0.922843 |
|  |  | CCP | -0.04319 | 0.873819 | 0.922843 |
|  |  | RF | -0.33315 | 0.207351 | 0.922843 |
|  |  | VAS | 0.288358 | 0.278771 | 0.922843 |
|  |  | TJC | 0.163418 | 0.545355 | 0.922843 |
|  |  | SJC | 0.058667 | 0.829129 | 0.922843 |
|  |  | HAQ | 0.344284 | 0.191627 | 0.922843 |
|  |  | DAS28. | 0.168755 | 0.532123 | 0.922843 |
|  |  | IS | -0.02854 | 0.922843 | 0.922843 |
|  | Lymphoid | ESR | -0.08294 | 0.588066 | 0.811458 |
|  |  | CRP | -0.15526 | 0.314233 | 0.785583 |
|  |  | CCP | 0.235567 | 0.119286 | 0.397621 |
|  |  | RF | -0.03895 | 0.799493 | 0.811458 |
|  |  | VAS | -0.03658 | 0.811458 | 0.811458 |
|  |  | TJC | -0.25489 | 0.091072 | 0.397621 |
|  |  | SJC | -0.11839 | 0.43858 | 0.811458 |
|  |  | HAQ | 0.041109 | 0.788607 | 0.811458 |
|  |  | DAS28. | -0.08543 | 0.576847 | 0.811458 |
|  |  | IS | 0.24193 | 0.109348 | 0.397621 |
|  | Myeloid | ESR | -0.16024 | 0.499769 | 0.693972 |
|  |  | CRP | -0.14995 | 0.528033 | 0.693972 |
|  |  | CCP | 0.154904 | 0.514337 | 0.693972 |
|  |  | RF | 0.170105 | 0.473373 | 0.693972 |
|  |  | VAS | 0.515612 | 0.01997 | 0.199698 |
|  |  | TJC | 0.068114 | 0.775389 | 0.861543 |
|  |  | SJC | 0.027411 | 0.908672 | 0.908672 |
|  |  | HAQ | 0.222422 | 0.360068 | 0.693972 |
|  |  | DAS28. | 0.140312 | 0.555177 | 0.693972 |
|  |  | IS | -0.14939 | 0.554097 | 0.693972 |
| CD52+ Mo-Mp | All | ESR | 0.156064 | 0.164138 | 0.273563 |
|  |  | CRP | 0.017884 | 0.874884 | 0.874884 |
|  |  | CCP | 0.171496 | 0.12581 | 0.260795 |
|  |  | RF | 0.122643 | 0.27538 | 0.3934 |
|  |  | VAS | 0.25659 | 0.020762 | 0.103812 |
|  |  | TJC | 0.060889 | 0.589206 | 0.654674 |
|  |  | SJC | 0.096606 | 0.39092 | 0.48865 |
|  |  | HAQ | 0.170549 | 0.130398 | 0.260795 |
|  |  | DAS28. | 0.192938 | 0.084401 | 0.260795 |
|  |  | IS | 0.502024 | 3.29E-06 | 3.29E-05 |
|  | Fibroid | ESR | 0.33601 | 0.20323 | 0.406459 |
|  |  | CRP | 0.163283 | 0.545692 | 0.682115 |
|  |  | CCP | 0.124216 | 0.646707 | 0.71184 |
|  |  | RF | 0.100245 | 0.71184 | 0.71184 |
|  |  | VAS | 0.591111 | 0.01589 | 0.039726 |
|  |  | TJC | 0.640585 | 0.007507 | 0.029972 |
|  |  | SJC | 0.30289 | 0.254151 | 0.423586 |
|  |  | HAQ | 0.632132 | 0.008608 | 0.029972 |
|  |  | DAS28. | 0.629388 | 0.008992 | 0.029972 |
|  |  | IS | -0.19444 | 0.505348 | 0.682115 |
|  | Lymphoid | ESR | -0.02945 | 0.847707 | 0.847707 |
|  |  | CRP | -0.07282 | 0.638554 | 0.798193 |
|  |  | CCP | 0.167348 | 0.271861 | 0.679653 |
|  |  | RF | 0.113717 | 0.457004 | 0.761674 |
|  |  | VAS | 0.185721 | 0.221914 | 0.679653 |
|  |  | TJC | -0.16842 | 0.268759 | 0.679653 |
|  |  | SJC | -0.08769 | 0.566788 | 0.798193 |
|  |  | HAQ | 0.118841 | 0.436842 | 0.761674 |
|  |  | DAS28. | -0.04858 | 0.751337 | 0.834819 |
|  |  | IS | 0.293582 | 0.050305 | 0.503049 |
|  | Myeloid | ESR | 0.048541 | 0.838962 | 0.966147 |
|  |  | CRP | -0.03433 | 0.885735 | 0.966147 |
|  |  | CCP | 0.010143 | 0.966147 | 0.966147 |
|  |  | RF | -0.16628 | 0.483524 | 0.690748 |
|  |  | VAS | 0.172585 | 0.466851 | 0.690748 |
|  |  | TJC | 0.251227 | 0.285324 | 0.690748 |
|  |  | SJC | 0.37028 | 0.108045 | 0.690748 |
|  |  | HAQ | -0.18588 | 0.44613 | 0.690748 |
|  |  | DAS28. | 0.29437 | 0.207728 | 0.690748 |
|  |  | IS | 0.178982 | 0.477326 | 0.690748 |
| CLEC10A+ Mo | All | ESR | 0.167349 | 0.135363 | 0.376359 |
|  |  | CRP | 0.002332 | 0.983618 | 0.983618 |
|  |  | CCP | 0.129464 | 0.249352 | 0.498704 |
|  |  | RF | 0.093058 | 0.408636 | 0.583766 |
|  |  | VAS | 0.227729 | 0.040889 | 0.204446 |
|  |  | TJC | 0.009147 | 0.935408 | 0.983618 |
|  |  | SJC | 0.013872 | 0.902179 | 0.983618 |
|  |  | HAQ | 0.110713 | 0.328236 | 0.547061 |
|  |  | DAS28. | 0.161194 | 0.150544 | 0.376359 |
|  |  | IS | 0.481114 | 9.46E-06 | 9.46E-05 |
|  | Fibroid | ESR | 0.071829 | 0.79151 | 0.855325 |
|  |  | CRP | 0.049576 | 0.855325 | 0.855325 |
|  |  | CCP | -0.23301 | 0.385138 | 0.550196 |
|  |  | RF | -0.40782 | 0.11687 | 0.474636 |
|  |  | VAS | 0.36878 | 0.159846 | 0.474636 |
|  |  | TJC | 0.345576 | 0.189854 | 0.474636 |
|  |  | SJC | -0.05248 | 0.846932 | 0.855325 |
|  |  | HAQ | 0.43905 | 0.088867 | 0.474636 |
|  |  | DAS28. | 0.265651 | 0.320015 | 0.543529 |
|  |  | IS | -0.28343 | 0.326117 | 0.543529 |
|  | Lymphoid | ESR | 0.018689 | 0.903018 | 0.903018 |
|  |  | CRP | -0.04949 | 0.749718 | 0.83302 |
|  |  | CCP | 0.242228 | 0.108897 | 0.404213 |
|  |  | RF | 0.161792 | 0.288322 | 0.576645 |
|  |  | VAS | 0.142076 | 0.351859 | 0.586431 |
|  |  | TJC | -0.27236 | 0.070293 | 0.404213 |
|  |  | SJC | -0.23435 | 0.121264 | 0.404213 |
|  |  | HAQ | 0.059843 | 0.696173 | 0.83302 |
|  |  | DAS28. | -0.10491 | 0.492823 | 0.704033 |
|  |  | IS | 0.176079 | 0.247271 | 0.576645 |
|  | Myeloid | ESR | -0.076 | 0.750133 | 0.833481 |
|  |  | CRP | -0.10727 | 0.652599 | 0.815748 |
|  |  | CCP | -0.11037 | 0.643199 | 0.815748 |
|  |  | RF | -0.12432 | 0.601533 | 0.815748 |
|  |  | VAS | 0.236757 | 0.314889 | 0.787224 |
|  |  | TJC | 0.341624 | 0.140429 | 0.573938 |
|  |  | SJC | 0.421603 | 0.064103 | 0.573938 |
|  |  | HAQ | -0.11703 | 0.633265 | 0.815748 |
|  |  | DAS28. | 0.317753 | 0.172181 | 0.573938 |
|  |  | IS | 0.029269 | 0.908217 | 0.908217 |
| COL3A1+ Mp | All | ESR | -0.25028 | 0.024227 | 0.121135 |
|  |  | CRP | -0.26401 | 0.017965 | 0.121135 |
|  |  | CCP | 0.014824 | 0.895501 | 0.909809 |
|  |  | RF | -0.13018 | 0.246719 | 0.616798 |
|  |  | VAS | -0.1059 | 0.346725 | 0.693451 |
|  |  | TJC | 0.012785 | 0.909809 | 0.909809 |
|  |  | SJC | -0.06699 | 0.552386 | 0.789122 |
|  |  | HAQ | 0.044448 | 0.695432 | 0.869289 |
|  |  | DAS28. | -0.07472 | 0.507376 | 0.789122 |
|  |  | IS | -0.21302 | 0.062872 | 0.209572 |
|  | Fibroid | ESR | 0.032867 | 0.903822 | 0.903822 |
|  |  | CRP | -0.21812 | 0.417051 | 0.764353 |
|  |  | CCP | 0.319456 | 0.227781 | 0.764353 |
|  |  | RF | 0.137614 | 0.611291 | 0.764353 |
|  |  | VAS | 0.12028 | 0.657253 | 0.764353 |
|  |  | TJC | 0.298618 | 0.261245 | 0.764353 |
|  |  | SJC | 0.108959 | 0.687918 | 0.764353 |
|  |  | HAQ | 0.205945 | 0.444143 | 0.764353 |
|  |  | DAS28. | 0.239786 | 0.371059 | 0.764353 |
|  |  | IS | 0.174124 | 0.551612 | 0.764353 |
|  | Lymphoid | ESR | -0.19664 | 0.19544 | 0.488601 |
|  |  | CRP | -0.25694 | 0.092238 | 0.461191 |
|  |  | CCP | -0.07526 | 0.623167 | 0.988026 |
|  |  | RF | -0.3309 | 0.026404 | 0.264042 |
|  |  | VAS | -0.2035 | 0.179982 | 0.488601 |
|  |  | TJC | 0.032788 | 0.830692 | 0.988026 |
|  |  | SJC | 0.037286 | 0.807874 | 0.988026 |
|  |  | HAQ | -0.00994 | 0.948318 | 0.988026 |
|  |  | DAS28. | -0.0023 | 0.988026 | 0.988026 |
|  |  | IS | 0.096231 | 0.529458 | 0.988026 |
|  | Myeloid | ESR | -0.47843 | 0.032852 | 0.109508 |
|  |  | CRP | -0.435 | 0.055263 | 0.110527 |
|  |  | CCP | -0.01221 | 0.95924 | 0.993828 |
|  |  | RF | 0.170993 | 0.471032 | 0.672903 |
|  |  | VAS | -0.00185 | 0.993828 | 0.993828 |
|  |  | TJC | -0.44444 | 0.049609 | 0.110527 |
|  |  | SJC | -0.50157 | 0.024251 | 0.109508 |
|  |  | HAQ | 0.193186 | 0.428118 | 0.672903 |
|  |  | DAS28. | -0.49766 | 0.025561 | 0.109508 |
|  |  | IS | -0.15313 | 0.54409 | 0.680113 |
| FOLR2LYVE1+ Mo-Mp | All | ESR | -0.06708 | 0.55181 | 0.689762 |
|  |  | CRP | -0.08829 | 0.436083 | 0.622975 |
|  |  | CCP | 0.144927 | 0.196738 | 0.622975 |
|  |  | RF | 0.0231 | 0.83781 | 0.83781 |
|  |  | VAS | -0.05479 | 0.627129 | 0.696809 |
|  |  | TJC | -0.16811 | 0.13356 | 0.622975 |
|  |  | SJC | -0.14338 | 0.201616 | 0.622975 |
|  |  | HAQ | 0.094463 | 0.404579 | 0.622975 |
|  |  | DAS28. | -0.08975 | 0.42558 | 0.622975 |
|  |  | IS | 0.130631 | 0.257469 | 0.622975 |
|  | Fibroid | ESR | -0.35084 | 0.182746 | 0.48655 |
|  |  | CRP | -0.24539 | 0.359644 | 0.599406 |
|  |  | CCP | -0.30959 | 0.243275 | 0.48655 |
|  |  | RF | -0.51069 | 0.043236 | 0.216178 |
|  |  | VAS | -0.70318 | 0.002375 | 0.023754 |
|  |  | TJC | -0.06394 | 0.814024 | 0.896169 |
|  |  | SJC | -0.14876 | 0.582427 | 0.728034 |
|  |  | HAQ | -0.19246 | 0.475159 | 0.678798 |
|  |  | DAS28. | -0.31276 | 0.238228 | 0.48655 |
|  |  | IS | 0.038451 | 0.896169 | 0.896169 |
|  | Lymphoid | ESR | -0.05829 | 0.703704 | 0.87963 |
|  |  | CRP | 0.011472 | 0.941085 | 0.95088 |
|  |  | CCP | 0.260268 | 0.084221 | 0.655991 |
|  |  | RF | 0.068673 | 0.653982 | 0.87963 |
|  |  | VAS | -0.00945 | 0.95088 | 0.95088 |
|  |  | TJC | -0.22845 | 0.131198 | 0.655991 |
|  |  | SJC | -0.176 | 0.247486 | 0.824953 |
|  |  | HAQ | 0.112641 | 0.461303 | 0.87963 |
|  |  | DAS28. | -0.08264 | 0.589432 | 0.87963 |
|  |  | IS | 0.100578 | 0.510934 | 0.87963 |
|  | Myeloid | ESR | -0.08298 | 0.727974 | 0.80886 |
|  |  | CRP | -0.17729 | 0.454602 | 0.649431 |
|  |  | CCP | 0.261726 | 0.264988 | 0.649431 |
|  |  | RF | 0.223183 | 0.344229 | 0.649431 |
|  |  | VAS | 0.247319 | 0.293135 | 0.649431 |
|  |  | TJC | -0.19691 | 0.405365 | 0.649431 |
|  |  | SJC | -0.20991 | 0.374402 | 0.649431 |
|  |  | HAQ | 0.321565 | 0.179435 | 0.649431 |
|  |  | DAS28. | -0.08856 | 0.710412 | 0.80886 |
|  |  | IS | 0.048126 | 0.849596 | 0.849596 |
| IL1B+ Mp | All | ESR | -0.07787 | 0.489579 | 0.945134 |
|  |  | CRP | -0.15596 | 0.167143 | 0.835716 |
|  |  | CCP | 0.212978 | 0.056266 | 0.562655 |
|  |  | RF | 0.07047 | 0.531874 | 0.945134 |
|  |  | VAS | 0.007767 | 0.945134 | 0.945134 |
|  |  | TJC | 0.03657 | 0.745846 | 0.945134 |
|  |  | SJC | -0.01673 | 0.882182 | 0.945134 |
|  |  | HAQ | 0.015932 | 0.88845 | 0.945134 |
|  |  | DAS28. | 0.036391 | 0.747044 | 0.945134 |
|  |  | IS | -0.04112 | 0.722508 | 0.945134 |
|  | Fibroid | ESR | 0.003676 | 0.989219 | 0.989219 |
|  |  | CRP | -0.20261 | 0.451718 | 0.889389 |
|  |  | CCP | 0.309924 | 0.242734 | 0.889389 |
|  |  | RF | 0.295457 | 0.266571 | 0.889389 |
|  |  | VAS | 0.102663 | 0.705176 | 0.889389 |
|  |  | TJC | 0.208009 | 0.439488 | 0.889389 |
|  |  | SJC | 0.068689 | 0.80045 | 0.889389 |
|  |  | HAQ | 0.069907 | 0.796979 | 0.889389 |
|  |  | DAS28. | 0.156723 | 0.562157 | 0.889389 |
|  |  | IS | 0.212194 | 0.466436 | 0.889389 |
|  | Lymphoid | ESR | -0.14761 | 0.333212 | 0.793726 |
|  |  | CRP | -0.25135 | 0.099796 | 0.793726 |
|  |  | CCP | 0.123711 | 0.418145 | 0.793726 |
|  |  | RF | -0.10894 | 0.476235 | 0.793726 |
|  |  | VAS | -0.05304 | 0.729338 | 0.810375 |
|  |  | TJC | -0.19549 | 0.198124 | 0.793726 |
|  |  | SJC | -0.16306 | 0.284525 | 0.793726 |
|  |  | HAQ | -0.00063 | 0.996733 | 0.996733 |
|  |  | DAS28. | -0.06784 | 0.657919 | 0.810375 |
|  |  | IS | -0.07811 | 0.610021 | 0.810375 |
|  | Myeloid | ESR | 0.247343 | 0.293086 | 0.526251 |
|  |  | CRP | 0.432752 | 0.056676 | 0.283379 |
|  |  | CCP | 0.364521 | 0.114072 | 0.28518 |
|  |  | RF | 0.201093 | 0.395245 | 0.526251 |
|  |  | VAS | 0.156866 | 0.508956 | 0.544751 |
|  |  | TJC | 0.213792 | 0.365428 | 0.526251 |
|  |  | SJC | 0.613148 | 0.004043 | 0.040432 |
|  |  | HAQ | -0.19612 | 0.421001 | 0.526251 |
|  |  | DAS28. | 0.388975 | 0.090067 | 0.28518 |
|  |  | IS | -0.15288 | 0.544751 | 0.544751 |
| NUPR1+ Mp | All | ESR | -0.04683 | 0.678042 | 0.850647 |
|  |  | CRP | -0.1352 | 0.23181 | 0.646129 |
|  |  | CCP | 0.180737 | 0.10638 | 0.531898 |
|  |  | RF | 0.006684 | 0.952778 | 0.97731 |
|  |  | VAS | 0.046448 | 0.680518 | 0.850647 |
|  |  | TJC | -0.12703 | 0.258452 | 0.646129 |
|  |  | SJC | -0.05598 | 0.619646 | 0.850647 |
|  |  | HAQ | 0.054214 | 0.632919 | 0.850647 |
|  |  | DAS28. | 0.00321 | 0.97731 | 0.97731 |
|  |  | IS | 0.261351 | 0.021682 | 0.21682 |
|  | Fibroid | ESR | -0.20661 | 0.442631 | 0.926766 |
|  |  | CRP | -0.18072 | 0.503 | 0.926766 |
|  |  | CCP | 0.025004 | 0.926766 | 0.926766 |
|  |  | RF | -0.28079 | 0.292145 | 0.926766 |
|  |  | VAS | -0.061 | 0.822428 | 0.926766 |
|  |  | TJC | 0.048962 | 0.857099 | 0.926766 |
|  |  | SJC | -0.10191 | 0.707241 | 0.926766 |
|  |  | HAQ | 0.048911 | 0.857245 | 0.926766 |
|  |  | DAS28. | -0.09602 | 0.72352 | 0.926766 |
|  |  | IS | 0.06569 | 0.823448 | 0.926766 |
|  | Lymphoid | ESR | -0.11226 | 0.462829 | 0.764849 |
|  |  | CRP | -0.17241 | 0.263084 | 0.657711 |
|  |  | CCP | 0.204707 | 0.177367 | 0.603633 |
|  |  | RF | -0.07104 | 0.64284 | 0.764849 |
|  |  | VAS | -0.06146 | 0.688364 | 0.764849 |
|  |  | TJC | -0.22492 | 0.137417 | 0.603633 |
|  |  | SJC | -0.11218 | 0.463165 | 0.764849 |
|  |  | HAQ | 0.013601 | 0.929341 | 0.929341 |
|  |  | DAS28. | -0.07377 | 0.630095 | 0.764849 |
|  |  | IS | 0.202998 | 0.18109 | 0.603633 |
|  | Myeloid | ESR | -0.14119 | 0.552669 | 0.912389 |
|  |  | CRP | -0.21551 | 0.361486 | 0.903042 |
|  |  | CCP | 0.233418 | 0.321962 | 0.903042 |
|  |  | RF | 0.178483 | 0.451521 | 0.903042 |
|  |  | VAS | 0.352944 | 0.126912 | 0.903042 |
|  |  | TJC | -0.0459 | 0.847625 | 0.912389 |
|  |  | SJC | -0.07794 | 0.743962 | 0.912389 |
|  |  | HAQ | 0.218333 | 0.369194 | 0.903042 |
|  |  | DAS28. | 0.026291 | 0.912389 | 0.912389 |
|  |  | IS | -0.07279 | 0.774075 | 0.912389 |
| SPP1+ Mo-Mp | All | ESR | 0.078488 | 0.486123 | 0.810206 |
|  |  | CRP | -0.0508 | 0.654539 | 0.823667 |
|  |  | CCP | 0.134298 | 0.231964 | 0.579909 |
|  |  | RF | 0.030959 | 0.783802 | 0.870891 |
|  |  | VAS | 0.170977 | 0.126976 | 0.579909 |
|  |  | TJC | -0.04979 | 0.658933 | 0.823667 |
|  |  | SJC | 0.014385 | 0.898574 | 0.898574 |
|  |  | HAQ | 0.092039 | 0.416798 | 0.810206 |
|  |  | DAS28. | 0.137489 | 0.220956 | 0.579909 |
|  |  | IS | 0.217205 | 0.05776 | 0.577603 |
|  | Fibroid | ESR | -0.01115 | 0.967324 | 0.997311 |
|  |  | CRP | -0.04439 | 0.870332 | 0.997311 |
|  |  | CCP | 0.191574 | 0.477231 | 0.796334 |
|  |  | RF | 0.000917 | 0.997311 | 0.997311 |
|  |  | VAS | 0.20589 | 0.444268 | 0.796334 |
|  |  | TJC | 0.217116 | 0.419252 | 0.796334 |
|  |  | SJC | 0.191331 | 0.477801 | 0.796334 |
|  |  | HAQ | 0.196787 | 0.465092 | 0.796334 |
|  |  | DAS28. | 0.198719 | 0.460632 | 0.796334 |
|  |  | IS | 0.003797 | 0.98972 | 0.997311 |
|  | Lymphoid | ESR | 0.047298 | 0.757679 | 0.934781 |
|  |  | CRP | -0.04028 | 0.795192 | 0.934781 |
|  |  | CCP | 0.166 | 0.275797 | 0.853075 |
|  |  | RF | 0.012551 | 0.934781 | 0.934781 |
|  |  | VAS | 0.130648 | 0.392316 | 0.853075 |
|  |  | TJC | -0.2339 | 0.121994 | 0.853075 |
|  |  | SJC | -0.11877 | 0.437111 | 0.853075 |
|  |  | HAQ | 0.100363 | 0.511845 | 0.853075 |
|  |  | DAS28. | 0.028036 | 0.854942 | 0.934781 |
|  |  | IS | 0.106014 | 0.48824 | 0.853075 |
|  | Myeloid | ESR | -0.05427 | 0.820234 | 0.925988 |
|  |  | CRP | -0.19743 | 0.4041 | 0.8082 |
|  |  | CCP | 0.012429 | 0.958522 | 0.958522 |
|  |  | RF | -0.05859 | 0.806177 | 0.925988 |
|  |  | VAS | 0.2024 | 0.392117 | 0.8082 |
|  |  | TJC | 0.356167 | 0.123239 | 0.8082 |
|  |  | SJC | 0.24067 | 0.30672 | 0.8082 |
|  |  | HAQ | -0.05174 | 0.83339 | 0.925988 |
|  |  | DAS28. | 0.295581 | 0.205776 | 0.8082 |
|  |  | IS | -0.12756 | 0.613984 | 0.925988 |

ESR: Erythrocyte Sedimentation Rate

CRP: C-reactive Protein

CCP: Cyclic Citrullinated Peptide

RF: Rheumatoid Factor

VAS: Pain on Visual Analog Scale

TJC: Tender Joint Count

SJC: Swollen Joint Count

HAQ: Health Assessment Questionnaire

DAS28: Disease Activity Score (28 joints)

IS: Inflammatory Score

FDR: False Detection Rate
