# Supplementary material for: Deconvolution of synovial myeloid cell subsets across pathotypes and role of COL3A1+ macrophages in rheumatoid arthritis remission
Source: Front Immunol. 2024 Mar 26;15:1307748. doi: 10.3389/fimmu.2024.1307748 (PMC11005452; doi:10.3389/fimmu.2024.1307748)
Supplement: Supplementary file 14 [file DataSheet_1.docx]

**Supplementary Text 1.** Description and integration of individual scRNA-seq datasets

1. E-MTAB-8322-Disc: Alivernini, S., et al. Distinct synovial tissue macrophage subsets regulate inflammation and remission in rheumatoid arthritis. *Nat Med* (2020): Discovery Cohort: synovial tissues of 12 RA patients. Mp were sorted (CD11b+, CD64+, CD3-, CD19-, CD56, CD49-, CD117-, CD15-) using FACS. The single cells were sequenced on the Illumina HiSeq 4000 platform. The dataset was downloaded from EMBL-EBI with accession number E-MTAB-8322.

2. E-MTAB-8322-Vali: Alivernini, S., et al. Distinct synovial tissue macrophage subsets regulate inflammation and remission in rheumatoid arthritis. *Nat Med* (2020): Validation Cohort: synovial tissues of 5 RA patients. Myeloid cells were distinguished computationally (CD64+, CD11b+, CD14+, MARCO+, CD1c+ and LYZ+). The single cells were sequenced on the Illumina HiSeq 4000 platform. The dataset was downloaded from EMBL-EBI with accession number E-MTAB-8322. The dataset was downloaded from EMBL-EBI with accession number E-MTAB-8322.

3. SDY998: Zhang, F. et al. Defining inflammatory cell states in rheumatoid arthritis joint synovial tissues by integrating single-cell transcriptomics and mass cytometry. *Nat Immunol* (2019): synovial tissues of 17 RA patients. Mo were sorted (CD45+, CD14+) using FACS and sequenced on the Illumina HiSeq 2500 platform. The dataset was retrieved from Immport with study accession code SDY998.

4. SDY1599_SCP469: Wei, K., et al. Notch signaling drives synovial fibroblast identity and arthritis pathology. *Nature* (2020): synovial tissues of 12 RA patients. Leucocytes (CD45+) and endothelial cells (CD31+, CD146+) were sorted using FACS and sequenced on the Illumina Nextseq 500 platform. T The dataset with the accession code SDY1599-SCP469 was downloaded from ImmPort.

5. In-house data: synovial tissues of 15 RA patients. In brief, single Mp were sorted (CD68+) using FACS and sequenced on Illumina NovaSeq 6000 platform (see details in Materials and Methods).

We used the datasets processed and aligned as described in each method section of the original studies. Gene ID was converted using R package BioMart. Then, we performed a standardized Seurat workflow for each dataset.

Regarding the quality control for each dataset, we removed cells with high expression of mitochondrial and ribosomal genes, low or abnormally high gene count. The thresholds for each parameter in different datasets were determined individually and listed below.

Table. The parameters of quality control for Seurat workflow in the 5 datasets

| Dataset | Cell Filtering Thresholds (No. Features) | Mitochondrial Genes Expression Thresholds (%) | Hemoglobin Genes Expression Thresholds (%) |
| --- | --- | --- | --- |
| E-MTAB-8322-Disc | > 200 & < 4000 | < 25 | < 3 |
| E-MTAB-8322-Vali | > 200 & < 4000 | < 25 | < 3 |
| SDY998 | > 200 & < 5000 | < 25 | < 3 |
| SDY1599_SCP469 | > 500 & < 7000 | < 25 | < 3 |
| In-house data | > 200 & < 4000 | < 25 | < 3 |

Subsequently, we selected target cells (macrophages, monocytes and endothelial cells) from each dataset according to the metadata provided in the original studies. Leucocytes in SDY1599_SCP469 were clustered *de novo* and annotated using R package singleR computationally, which indicating their identity as Mp/Mo. We integrated myeloid cells of each dataset using the standardized Seurat protocol. Seurat Wrappers package was used for integration by Harmony and Liger. We applied Atom Sketch, Harmony, and Liger integration procedure after normalization, variable feature finding and scaling of the data.
